# Supplementary material for: Genomic and functional analyses of fungal and bacterial consortia that enable lignocellulose breakdown in goat gut microbiomes
Source: Nat Microbiol. 2021 Feb 1;6(4):499–511. doi: 10.1038/s41564-020-00861-0 (PMC8007473; doi:10.1038/s41564-020-00861-0)
Supplement: Supplementary file 1 — Supplementary Figs. 1–9, Tables 1–12 and references. [file 41564_2020_861_MOESM1_ESM.pdf]

natureresearch

https://doi.org/10.1038/s41564-020-00861-0

Supplementary information

Genomic and functional analyses of fungal and bacterial consortia that enable lignocellulose breakdown in goat gut microbiomes

In the format provided by the authors and unedited

Supplementary Material for

Genomic and functional analyses of fungal and bacterial consortia that enable lignocellulose breakdown in goat gut microbiomes

Xuefeng Peng[1,2], St. Elmo Wilken[1], Thomas S. Lankiewicz[1,3], Sean P. Gilmore[1], Jennifer L. Brown[1], John K. Henske[1], Candice L. Swift[1], Asaf Salamov[4], Kerrie Barry[4], Igor V. Grigoriev[4], Michael K. Theodorou[5], David L. Valentine[6], Michelle A. O'Malley*[1,3]

[1]Department of Chemical Engineering, University of California, Santa Barbara, CA, USA
[2]Marine Science Institute, University of California, Santa Barbara, CA, USA
[3]Joint BioEnergy Institute, Lawrence Berkeley National Laboratory, Berkeley, CA, USA
[4]Department of Energy Joint Genome Institute, Lawrence Berkeley National Laboratory, Berkeley, CA 94598 USA
[5]Department of Animal Production, Welfare and Veterinary Sciences, Harper Adams University, Newport, Shropshire, UK
[6]Department of Earth Science, University of California, Santa Barbara, CA, USA

*Corresponding author. Email: momalley@ucsb.edu

Contents

Supplementary Figures 1 - 9

Supplementary Tables 1 - 12

Supplementary References

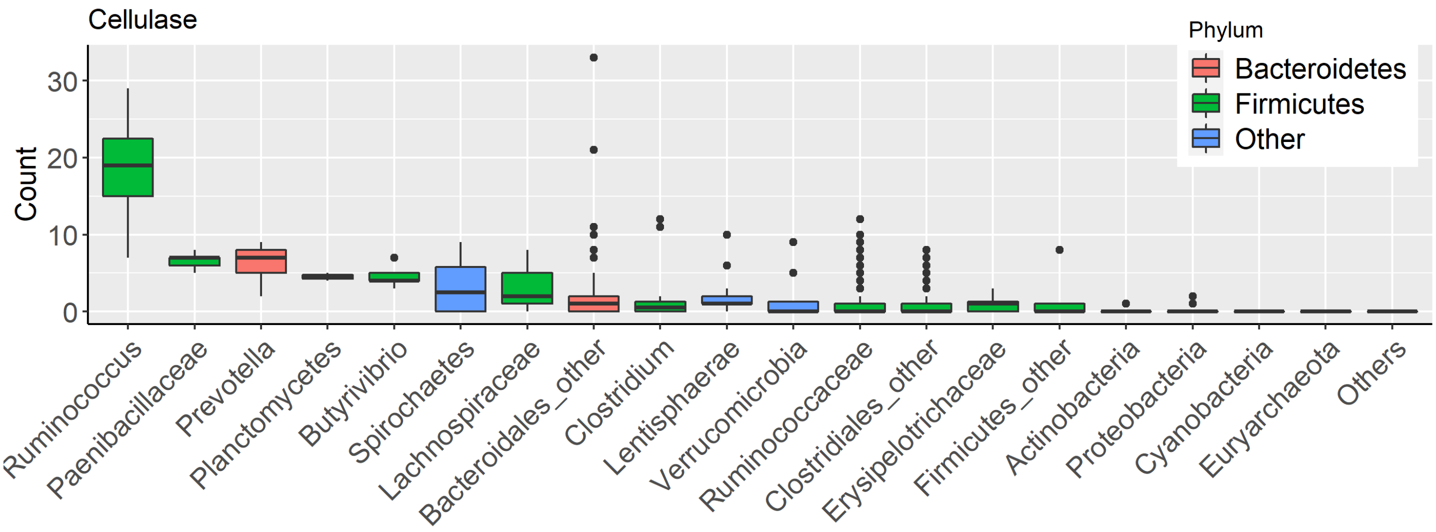

Supplementary Figure 1a. Box plot of the count of cellulases found in the collection of 719 metagenome-assembled genomes (MAGs) grouped by their taxonomy. The upper limit of the boxes corresponds to the third quartile, the lower limit of the boxes corresponds to the first quartile, and the line between them corresponds to the median. The end of the upper whisker marks the smaller value of the maximum count and the third quartile plus 1.5 times the inter-quartile range (IQR). The end of the lower whisker marks the greater value of the minimum count and the first quartile minus 1.5 times the IQR. Groups under "Other" are at the phylum level. *Bacteroidetes* were grouped into the genus *Prevotella* and the order *Bacteroidales* (excluding *Prevotella*). *Firmicutes* were grouped into the genera *Ruminococcus*, *Butyrivibrio*, and *Clostridium*, the families *Ruminococcaceae* (excluding *Ruminococcus*), *Lachnospiraceae* (excluding *Butyrivibrio), Paenibacillaceae*, and *Erysipelotrichaceae*, the order *Clostridiales* (excluding the groups at the genus and family levels), and other unclassified *Firmicutes*. The number of MAGs in each group is 19 for *Ruminococcus*, 8 for *Prevotella*, 9 for *Butyrivibrio*, 12 for *Clostridium*, 5 for *Paenibacillaceae*, 21 for *Lentisphaerae*, 279 for *Ruminococcaceae*, 65 for *Lachnospiraceae*, 20 for *Erysipelotrichaceae*, 77 for *Bacteroidales*_other, 86 for *Clostridiales*_other, 36 for *Firmicutes*_other, 8 for *Verrucomicrobia*, 2 for *Planctomyctes*, 10 for *Actinobacteria*, 23 for *Proteobacteria*, 6 for *Spirochaetes*, 7 for *Cyanobacteria*, 25 for *Euryarchaeota*, and 1 for Others.

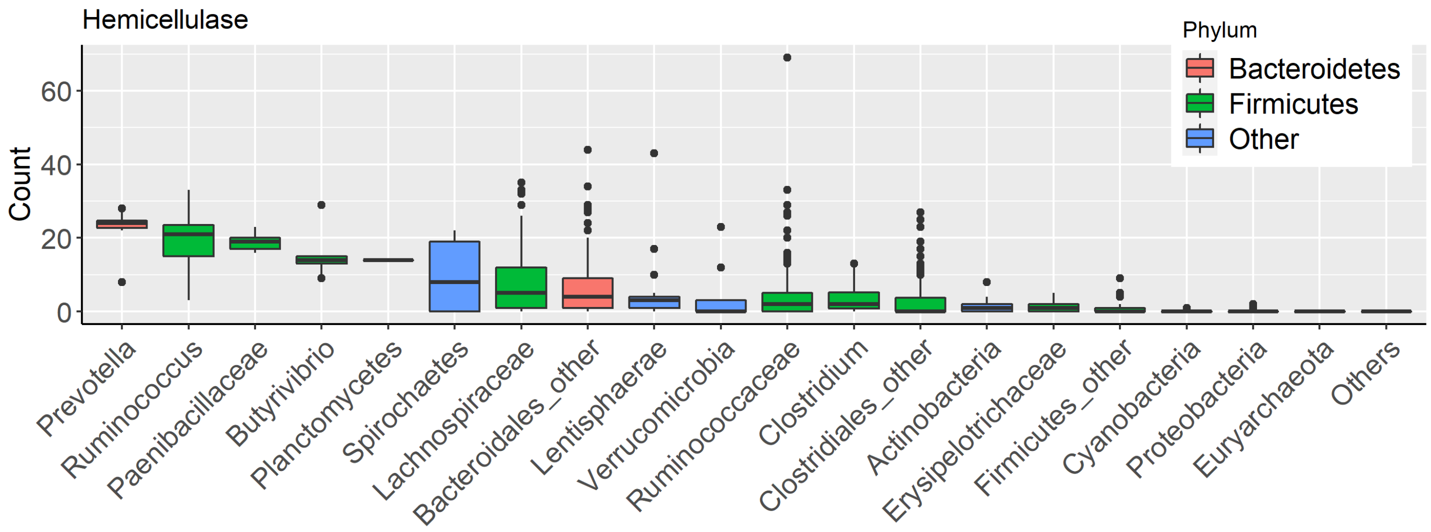

Supplementary Figure 1b. Box plot of the count of hemicellulases found in the collection of 719 metagenome-assembled genomes (MAGs) grouped by their taxonomy. The upper limit of the boxes corresponds to the third quartile, the lower limit of the boxes corresponds to the first quartile, and the line between them corresponds to the median. The end of the upper whisker marks the smaller value of the maximum count and the third quartile plus 1.5 times the inter-quartile range (IQR). The end of the lower whisker marks the greater value of the minimum count and the first quartile minus 1.5 times the IQR. Groups under "Other" are at the phylum level. *Bacteroidetes* were grouped into the genus *Prevotella* and the order *Bacteroidales* (excluding *Prevotella*). *Firmicutes* were grouped into the genera *Ruminococcus*, *Butyrivibrio*, and *Clostridium*, the families *Ruminococcaceae* (excluding *Ruminococcus*)*, Lachnospiraceae* (excluding *Butyrivibrio*)*, Paenibacillaceae*, and *Erysipelotrichaceae*, the order *Clostridiales* (excluding the groups at the genus and family levels), and other unclassified *Firmicutes*. The number of MAGs in each group is 19 for *Ruminococcus*, 8 for *Prevotella*, 9 for *Butyrivibrio*, 12 for *Clostridium*, 5 for *Paenibacillaceae*, 21 for *Lentisphaerae*, 279 for *Ruminococcaceae*, 65 for *Lachnospiraceae*, 20 for *Erysipelotrichaceae*, 77 for *Bacteroidales*_other, 86 for *Clostridiales*_other, 36 for *Firmicutes*_other, 8 for *Verrucomicrobia*, 2 for *Planctomyctes*, 10 for *Actinobacteria*, 23 for *Proteobacteria*, 6 for *Spirochaetes*, 7 for *Cyanobacteria*, 25 for *Euryarchaeota*, and 1 for Others.

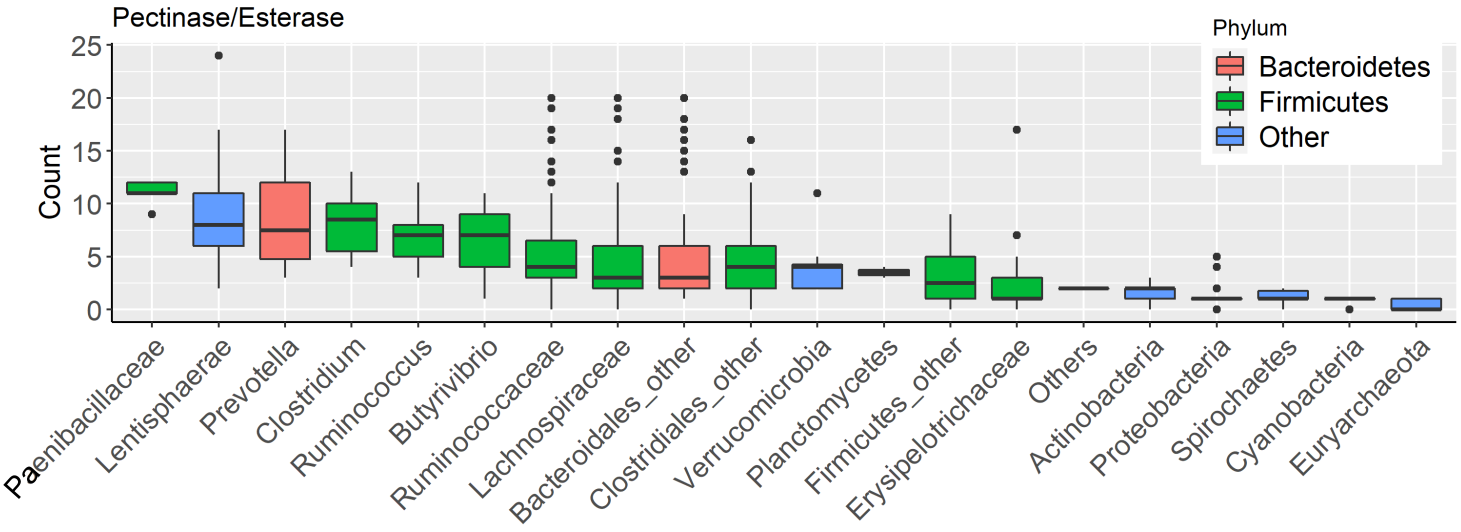

Supplementary Figure 1c. Box plot of the count of pectinases/esterases found in the collection of 719 metagenome-assembled genomes (MAGs) grouped by their taxonomy. The upper limit of the boxes corresponds to the third quartile, the lower limit of the boxes corresponds to the first quartile, and the line between them corresponds to the median. The end of the upper whisker marks the smaller value of the maximum count and the third quartile plus 1.5 times the inter-quartile range (IQR). The end of the lower whisker marks the greater value of the minimum count and the first quartile minus 1.5 times the IQR. Groups under "Other" are at the phylum level. *Bacteroidetes* were grouped into the genus *Prevotella* and the order *Bacteroidales* (excluding *Prevotella*). *Firmicutes* were grouped into the genera *Ruminococcus*, *Butyrivibrio*, and *Clostridium*, the families *Ruminococcaceae* (excluding *Ruminococcus*), *Lachnospiraceae* (excluding *Butyrivibrio), Paenibacillaceae*, and *Erysipelotrichaceae*, the order *Clostridiales* (excluding the groups at the genus and family levels), and other unclassified *Firmicutes*. The number of MAGs in each group is 19 for *Ruminococcus*, 8 for *Prevotella*, 9 for *Butyrivibrio*, 12 for *Clostridium*, 5 for *Paenibacillaceae*, 21 for *Lentisphaerae*, 279 for *Ruminococcaceae*, 65 for *Lachnospiraceae*, 20 for *Erysipelotrichaceae*, 77 for *Bacteroidales*_other, 86 for *Clostridiales*_other, 36 for *Firmicutes*_other, 8 for *Verrucomicrobia*, 2 for *Planctomyctes*, 10 for *Actinobacteria*, 23 for *Proteobacteria*, 6 for *Spirochaetes*, 7 for *Cyanobacteria*, 25 for *Euryarchaeota*, and 1 for Others.

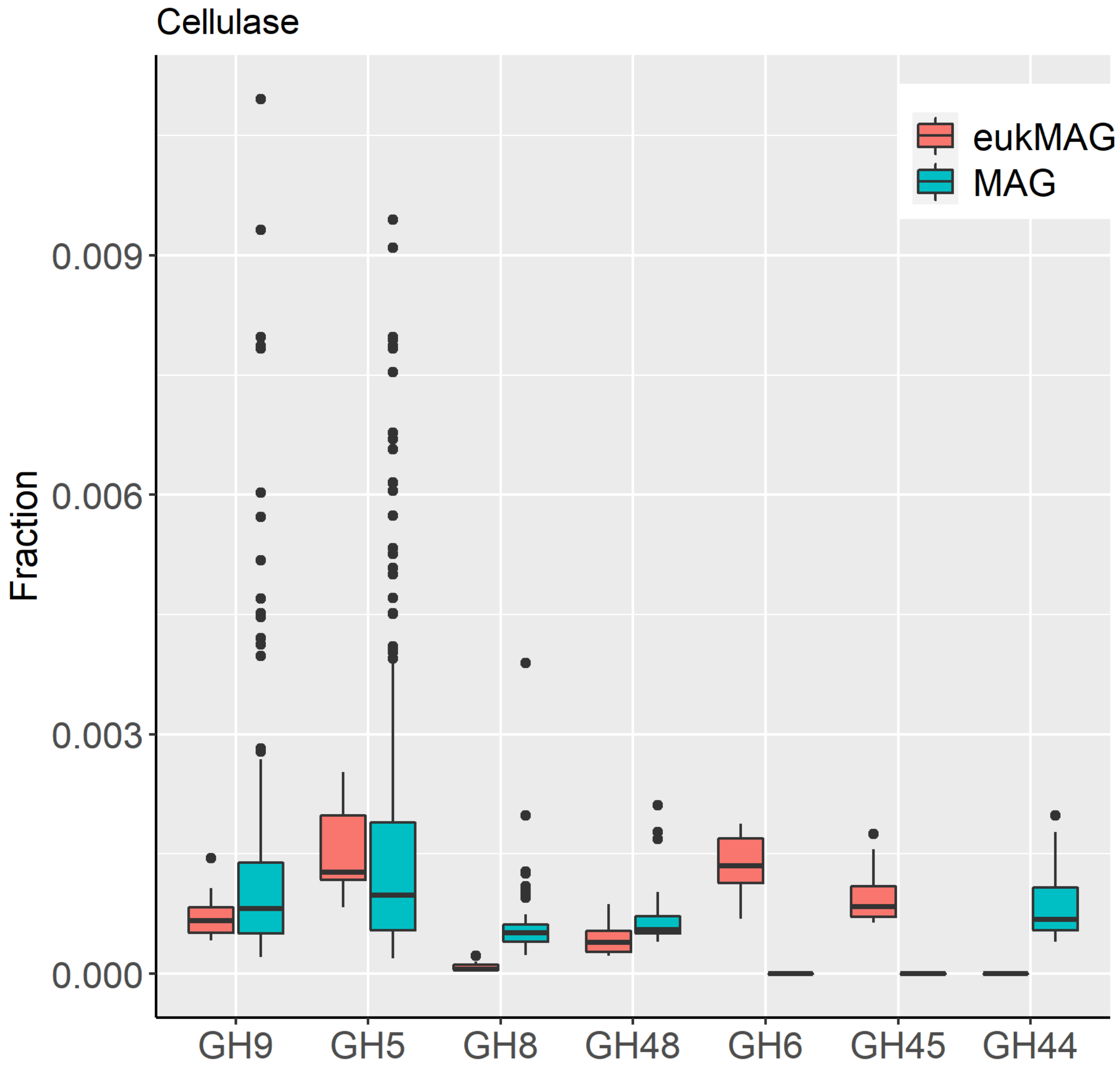

Supplementary Figure 2a. Box plot of the fraction of different carbohydrate-active enzyme (CAZyme) families that are classified as cellulase. The upper limit of the boxes corresponds to the third quartile, the lower limit of the boxes corresponds to the first quartile, and the line between them corresponds to the median. The end of the upper whisker marks the smaller value of the maximum count and the third quartile plus 1.5 times the inter-quartile range (IQR). The end of the lower whisker marks the greater value of the minimum count and the first quartile minus 1.5 times the IQR. Fractions are calculated by dividing the number of CAZyme family from a prokaryotic metagenome-assembled genome (MAG) or a eukaryotic metagenome-assembled genome (eukMAG) by the total number of open reading frames in the corresponding MAG or eukMAG. Glycosyl hydrolase (GH) families 6 and 45 were found only in eukMAGs, and GH44 were found only in prokaryotic MAGs. Within each CAZyme family, only nonzero counts were included in this figure. The number of nonzero counts, for eukMAGs and MAGs, respectively, was 18 and 140 for GH9, 18 and 312 for GH5, 18 and 53 for GH8, 18 and 22 for GH48, 18 and 0 for GH6 and GH45, and 0 and 20 for GH44.

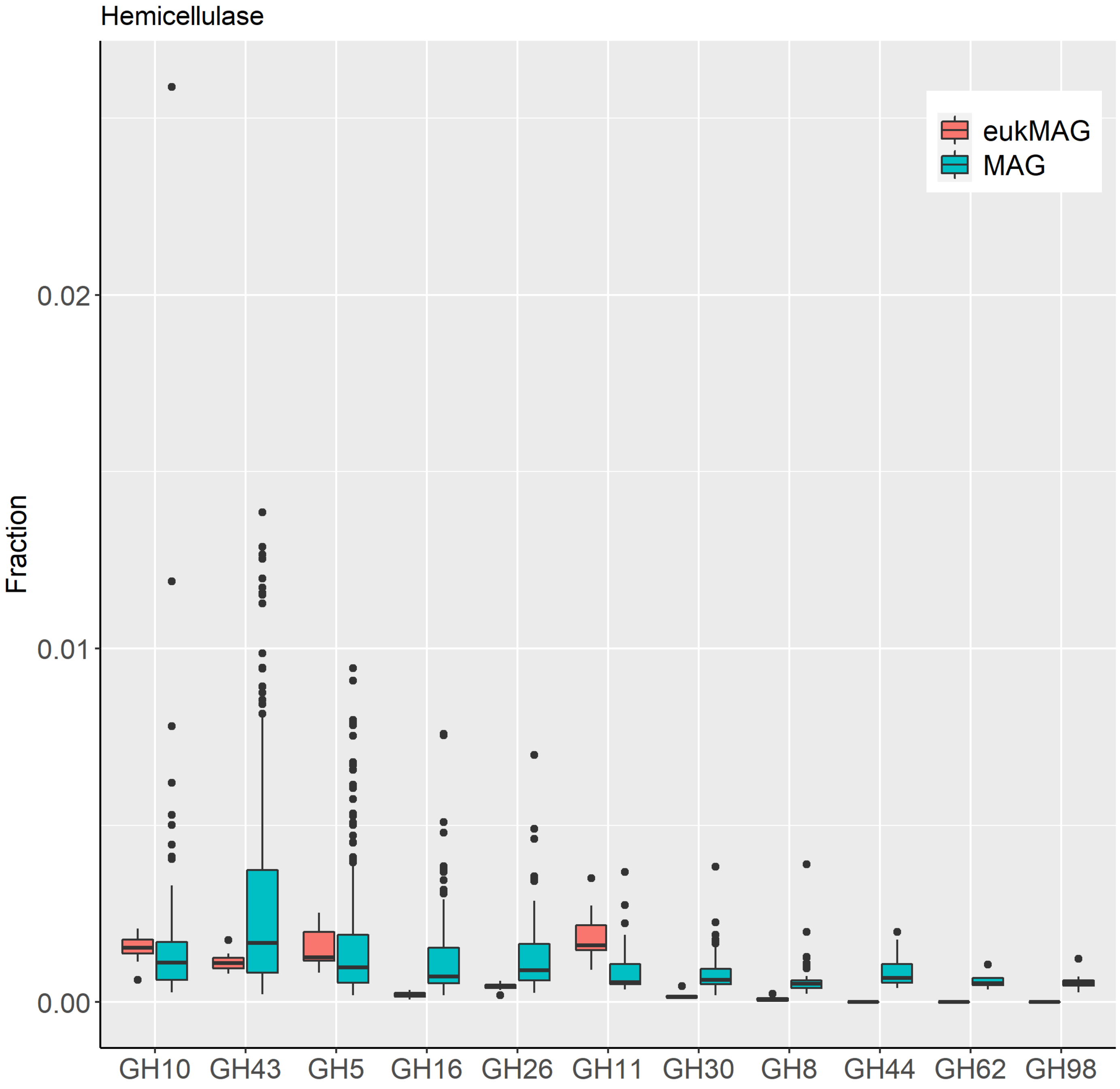

Supplementary Figure 2b. Box plot of the fraction of different carbohydrate-active enzyme (CAZyme) families that are classified as hemicellulase. The upper limit of the boxes corresponds to the third quartile, the lower limit of the boxes corresponds to the first quartile, and the line between them corresponds to the median. The end of the upper whisker marks the smaller value of the maximum count and the third quartile plus 1.5 times the inter-quartile range (IQR). The end of the lower whisker marks the greater value of the minimum count and the first quartile minus 1.5 times the IQR. Fractions are calculated by dividing the number of CAZyme family from a prokaryotic metagenome-assembled genome (MAG) or a eukaryotic metagenome-assembled genome (eukMAG) by the total number of open reading frames in the corresponding MAG or eukMAG. Glycosyl hydrolase (GH) families 44, 62, and 98 were found only in prokaryotic MAGs. Within each CAZyme family, only nonzero counts were included in this figure. The number of nonzero counts, for eukMAGs and MAGs, respectively, was 18 and 167 for GH10, 18 and 360 for GH43, 18 and 312 for GH5, 18 and 178 for GH16, 18 and 115 for GH26, 18 and 30 for GH11, 18 and 151 for GH30, 18 and 53 for GH8, 0 and 20 for GH44, 0 and 4 for GH62, and 0 and 16 for GH98.

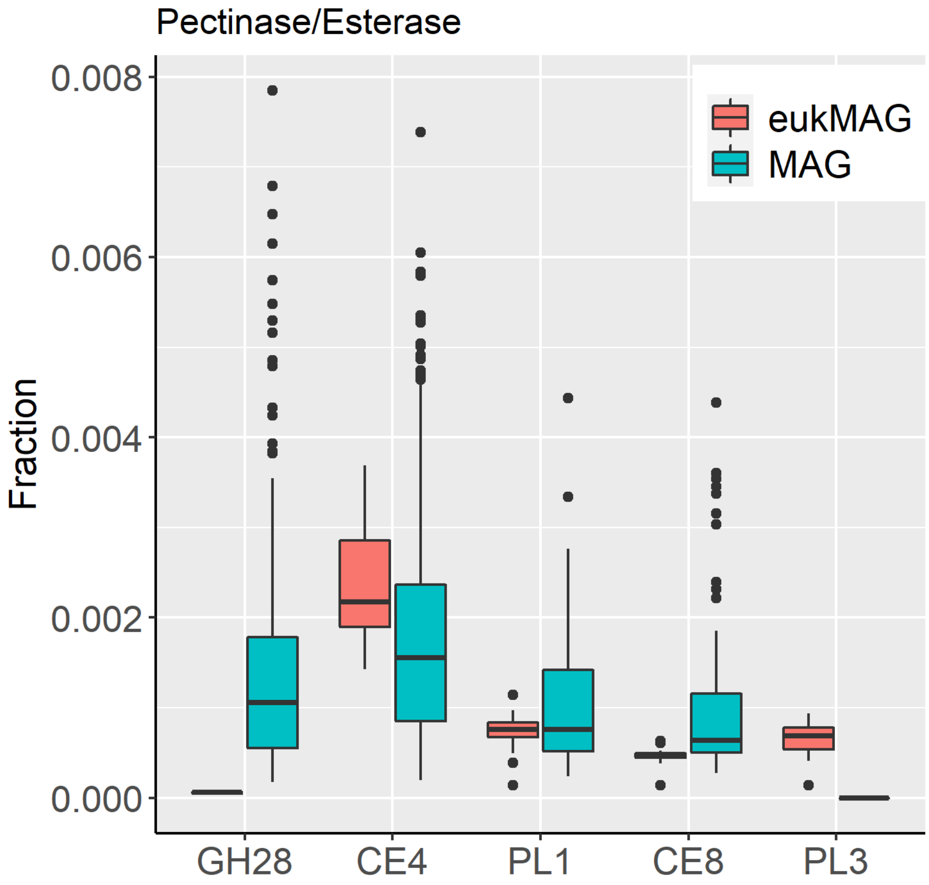

Supplementary Figure 2c. Box plot of the fraction of different carbohydrate-active enzyme (CAZyme) families that are classified as pectinase/esterase. The upper limit of the boxes corresponds to the third quartile, the lower limit of the boxes corresponds to the first quartile, and the line between them corresponds to the median. The end of the upper whisker marks the smaller value of the maximum count and the third quartile plus 1.5 times the inter-quartile range (IQR). The end of the lower whisker marks the greater value of the minimum count and the first quartile minus 1.5 times the IQR. Fractions are calculated by dividing the number of CAZyme family from a prokaryotic metagenome-assembled genome (MAG) or a eukaryotic metagenome-assembled genome (eukMAG) by the total number of open reading frames in the corresponding MAG or eukMAG. Glycosyl hydrolase (GH) family 28 was found only in prokaryotic MAGs, and pectin lyase (PL) family 3 were found only in eukMAGs. "CE" represents carbohydrate esterase. Within each CAZyme family, only nonzero counts were included in this figure. The number of nonzero counts, for eukMAGs and MAGs, respectively, was 5 and 267 for GH28, 18 and 658 for CE4, 18 and 93 for CE8 18 and 54 for PL1, and 18 and 0 for PL3.

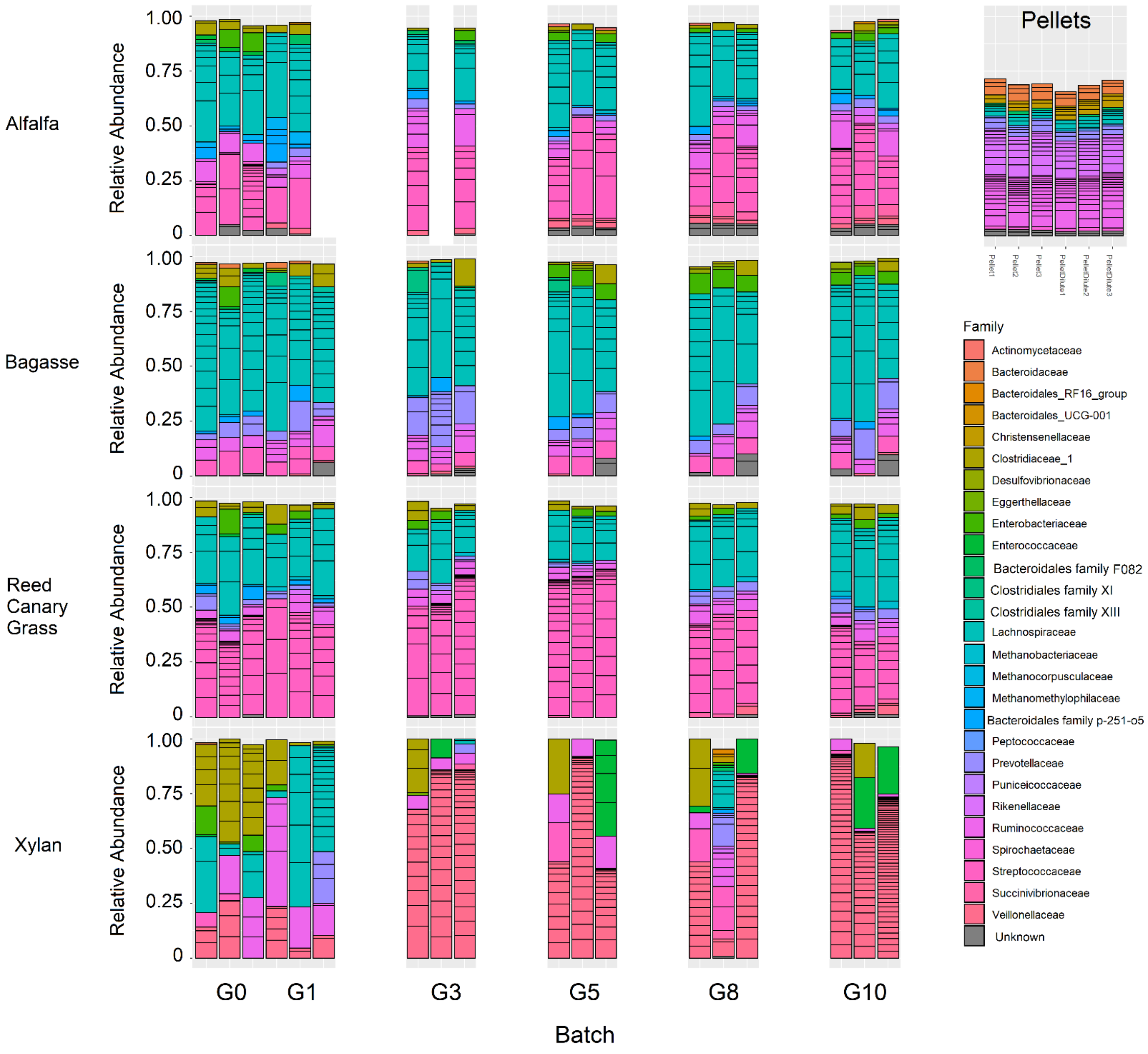

Supplementary Figure 3a. Microbial community composition in antibiotics-free consortia and fecal pellets evaluated by the V4 region of the 16S rRNA gene, as a function of selected batches (G0, G1, G3, G5, G8, and G10) and carbon substrate. The three bars adjacent to each other represent three biological replicates. Because this figure presents the top 1500 most abundant amplicon sequence variants (ASVs), the relative abundance in a sample does not necessarily sum to one. In addition to the three pellet samples, there were also three "pellet_dilute" samples that show the community composition of eight pellets homogenized in culture medium "MC-" (see methods). Amplicon sequencing reads were processed in R using the package DADA2 version 1.8.0 and the figure was generated in R with the package phyloseq version 1.26.1.

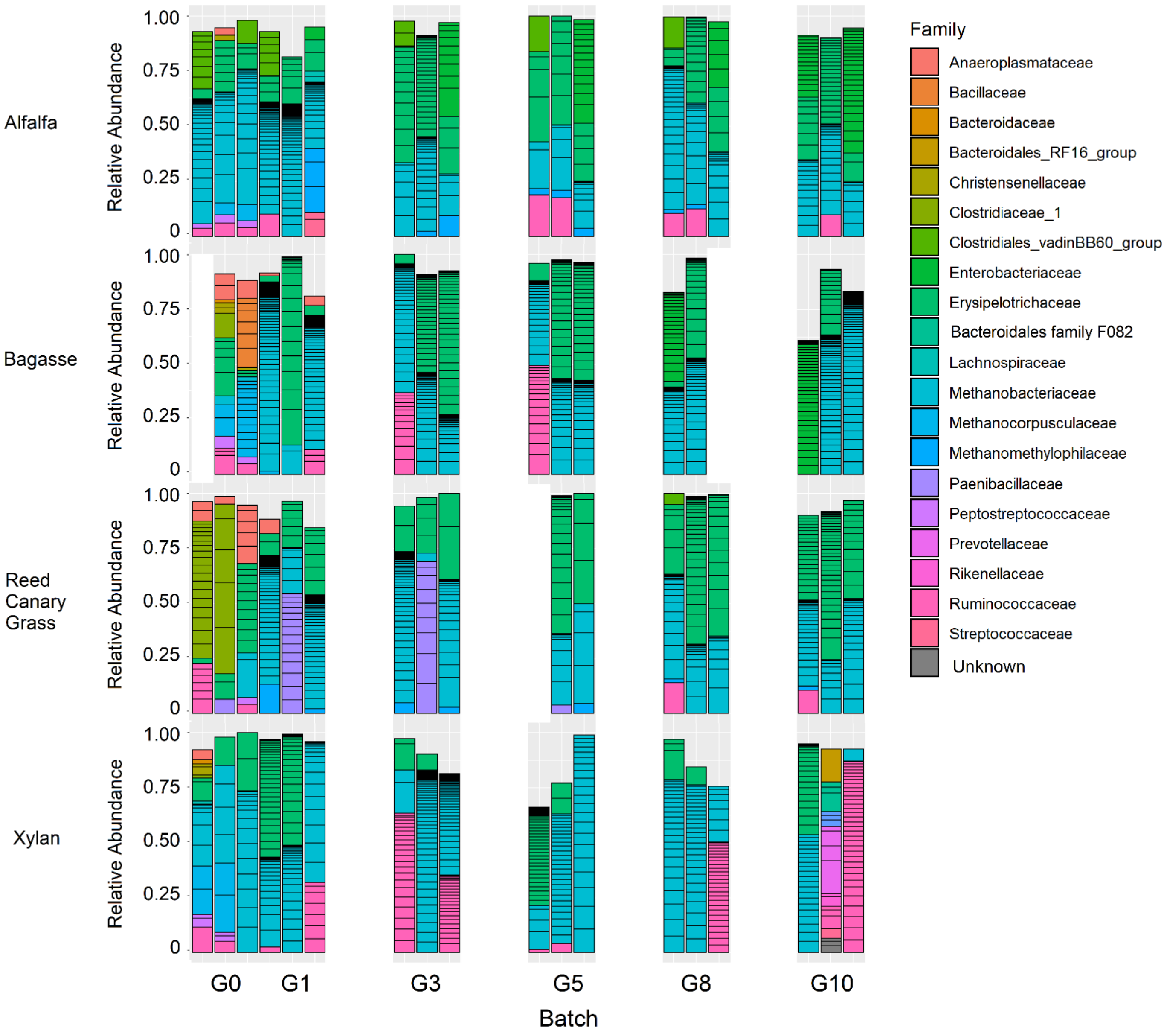

Supplementary Figure 3b. Microbial community composition in consortia treated with penicillin and streptomycin and fecal pellets evaluated by the V4 region of the 16S rRNA gene, as a function of selected batches (G0, G1, G3, G5, G8, and G10) and carbon substrate. The three bars adjacent to each other represent three biological replicates. Because this figure presents the top 1500 most abundant amplicon sequence variants (ASVs), the relative abundance in a sample does not necessarily sum to one. In addition to the three pellet samples, there were also three "pellet_dilute" samples that show the community composition of eight pellets homogenized in culture medium "MC-" (see methods). Amplicon sequencing reads were processed in R using the package DADA2 version 1.8.0 and the figure was generated in R with the package phyloseq version 1.26.1.

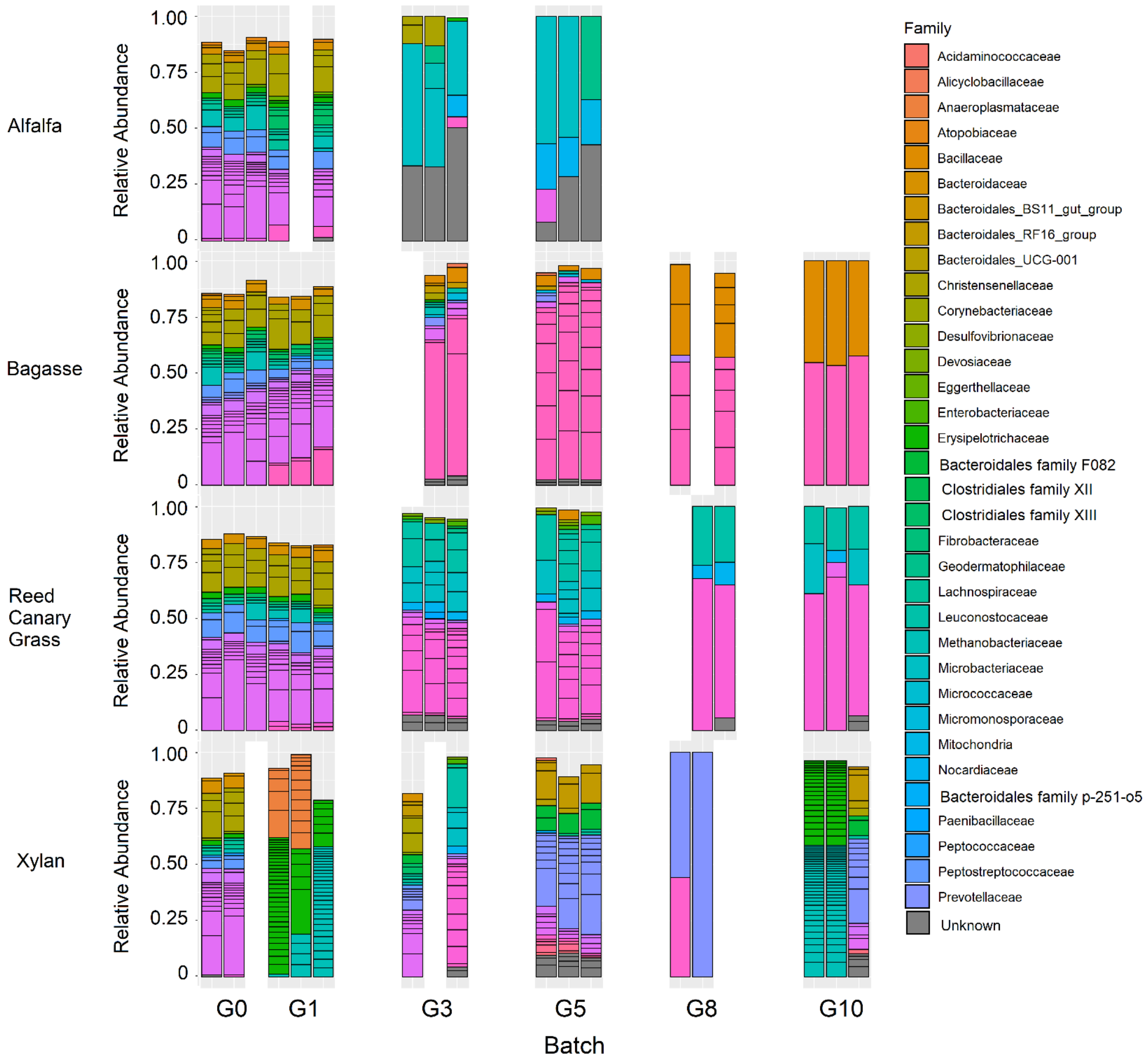

Supplementary Figure 3c. Microbial community composition in consortia treated with chloramphenicol and fecal pellets evaluated by the V4 region of the 16S rRNA gene, as a function of selected batches (G0, G1, G3, G5, G8, and G10) and carbon substrate. The three bars adjacent to each other represent three biological replicates. Because this figure presents the top 1000 most abundant amplicon sequence variants (ASVs), the relative abundance in a sample does not necessarily sum to one. In addition to the three pellet samples, there were also three “pellet_dilute” samples that show the community composition of eight pellets homogenized in culture medium “MC-” (see methods). Amplicon sequencing reads were processed in R using the package DADA2 version 1.8.0 and the figure was generated in R with the package phyloseq version 1.26.1.

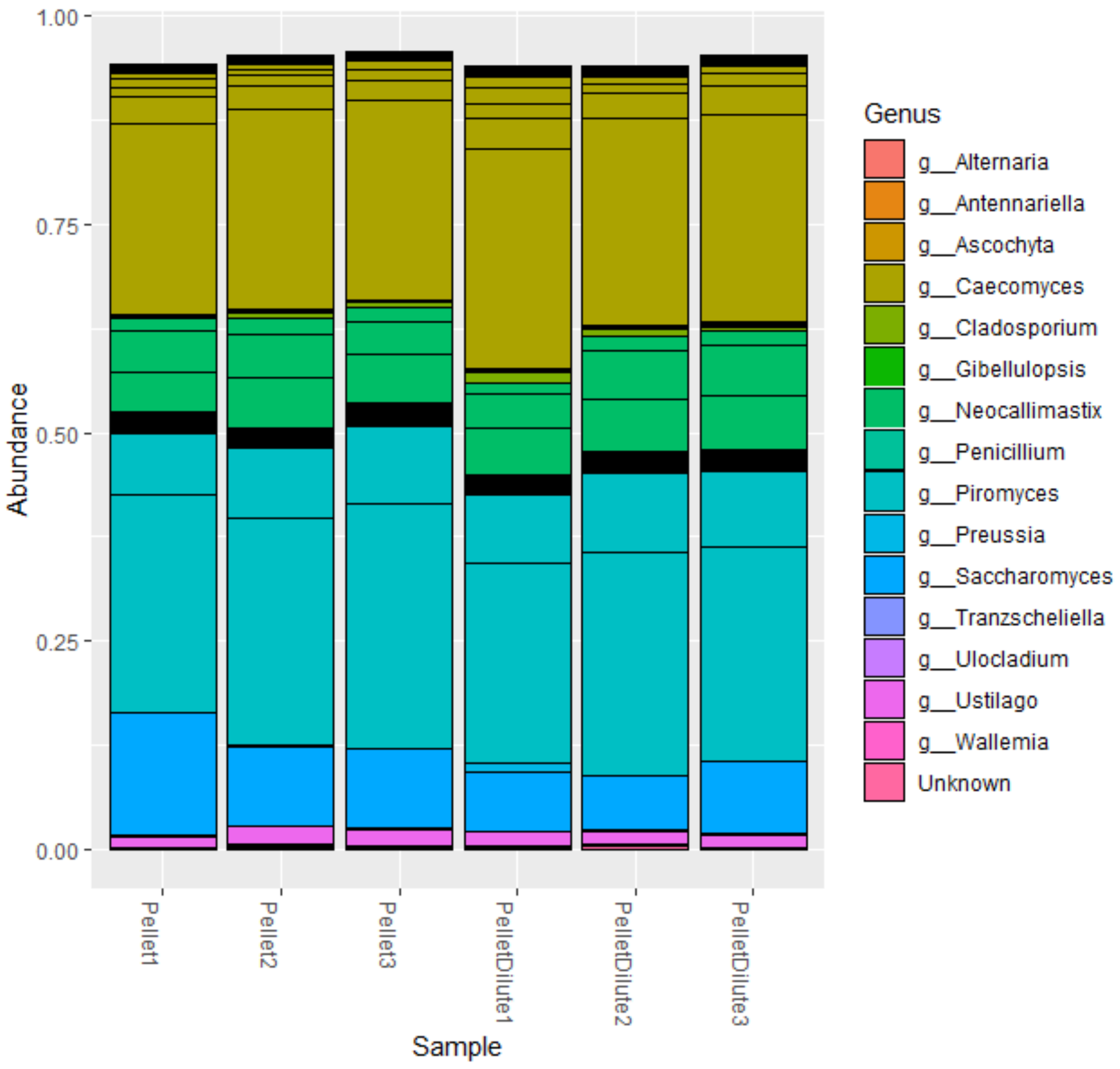

Supplementary Figure 3d. Microbial community composition in fecal pellets evaluated by the internal transcribed spacer region 2 (ITS2). Because this figure presents the top 50 most abundant amplicon sequence variants (ASVs), the relative abundance in a sample does not necessarily sum to one. In addition to the three pellet samples, there were also three "pellet_dilute" samples that show the community composition of eight pellets homogenized in culture medium "MC-" (see methods). The five most abundant genera are *Caecomyces*, *Neocallimastix*, *Piromyces*, *Saccharomyces*, and *Ustilago*. Amplicon sequencing reads were processed in R using the package DADA2 version 1.8.0 and the figure was generated in R with the package phyloseq version 1.26.1.

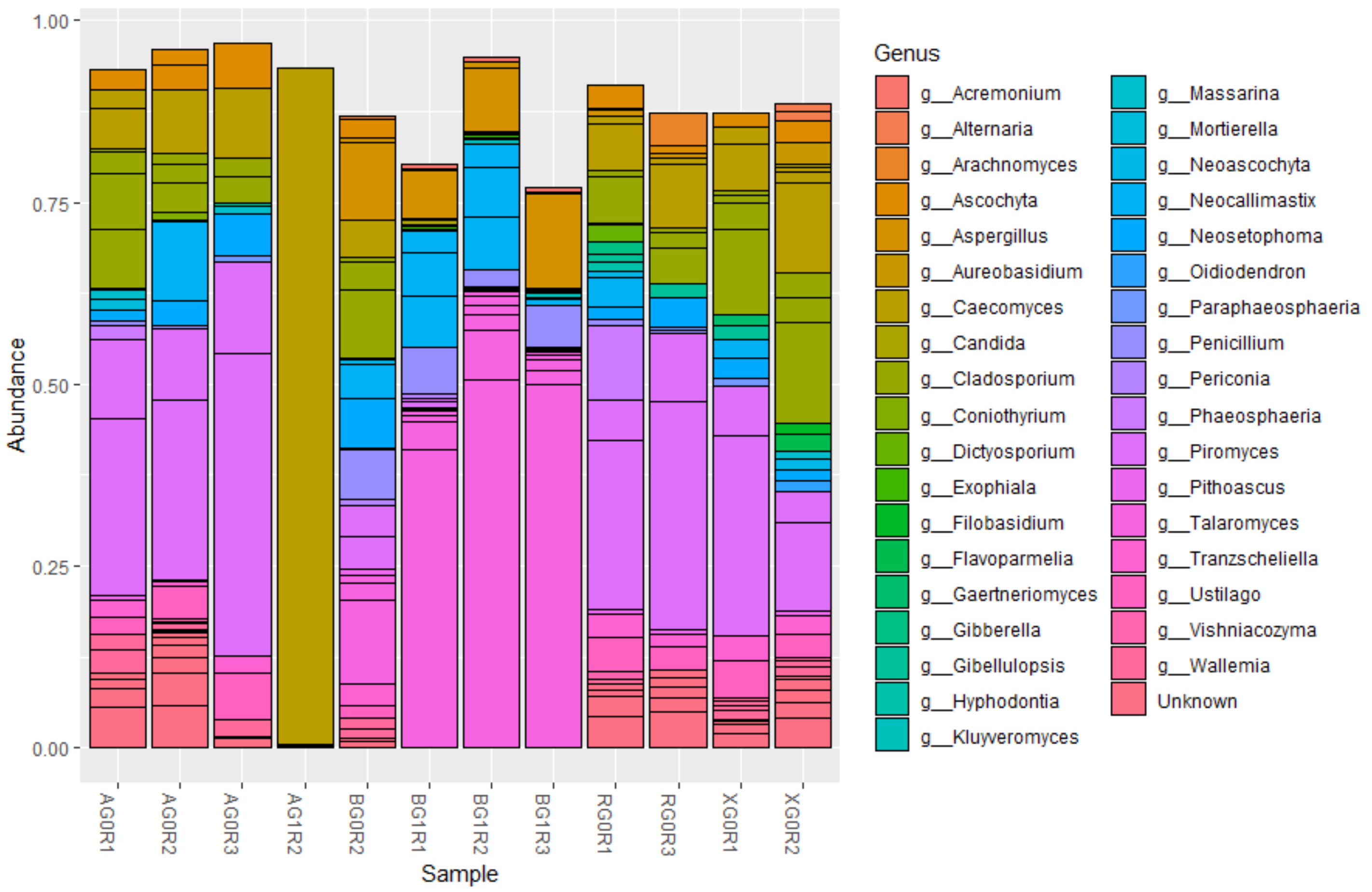

Supplementary Figure 3e. Microbial community composition in antibiotics-free consortia evaluated by the internal transcribed spacer region 2 (ITS2). Because this figure presents amplicon sequence variants (ASVs) with a relative abundance greater than 0.1% when averaged among all samples, the relative abundance in a sample does not necessarily sum to one. Amplicon sequencing reads were processed in R using the package DADA2 version 1.8.0 and the figure was generated in R with the package phyloseq version 1.26.1. For each sample from each batch we have provided a unique identifier with the format "SGxRy", where "S" represent the carbon substrate ("A" for alfalfa stems, "B" for bagasse, "R" for reed canary grass, and "X" for xylan), "x" represents the batch number (0 through 10), and "y" represents the replicate number (1, 2, or 3). Note that there were no ITS2 amplified after G1, and most taxa shown above are likely remainder of the fecal pellet.

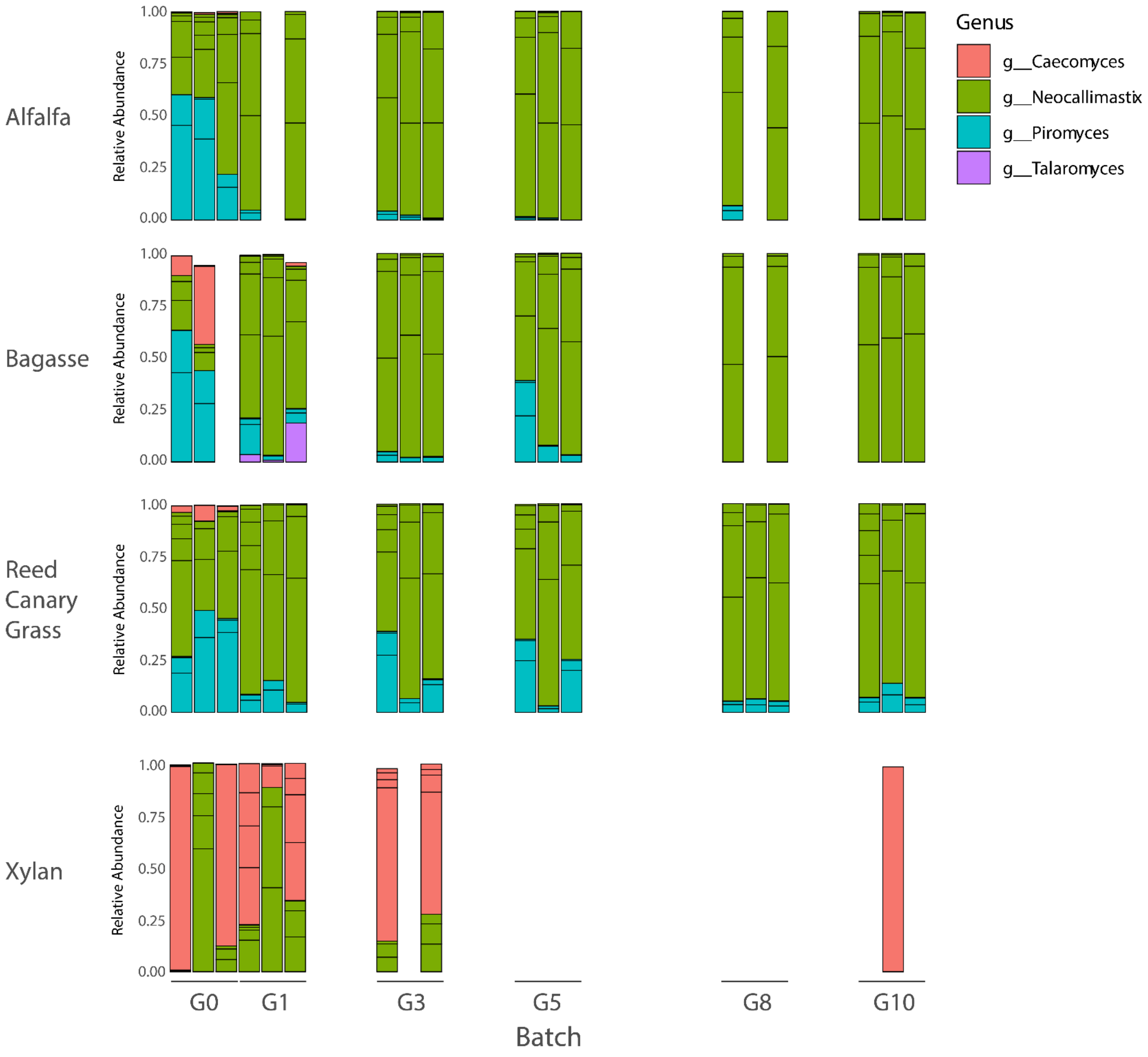

Supplementary Figure 3f. Microbial community composition in consortia treated with penicillin and streptomycin and fecal pellets evaluated by the internal transcribed spacer region 2 (ITS2), as a function of selected batches (G0, G1, G3, G5, G8, and G10) and carbon substrate. The three bars adjacent to each other represent three biological replicates. Because this figure presents the top 20 most abundant amplicon sequence variants (ASVs), the relative abundance in a sample does not necessarily sum to one. Amplicon sequencing reads were processed in R using the package DADA2 version 1.8.0 and the figure was generated in R with the package phyloseq version 1.26.1.

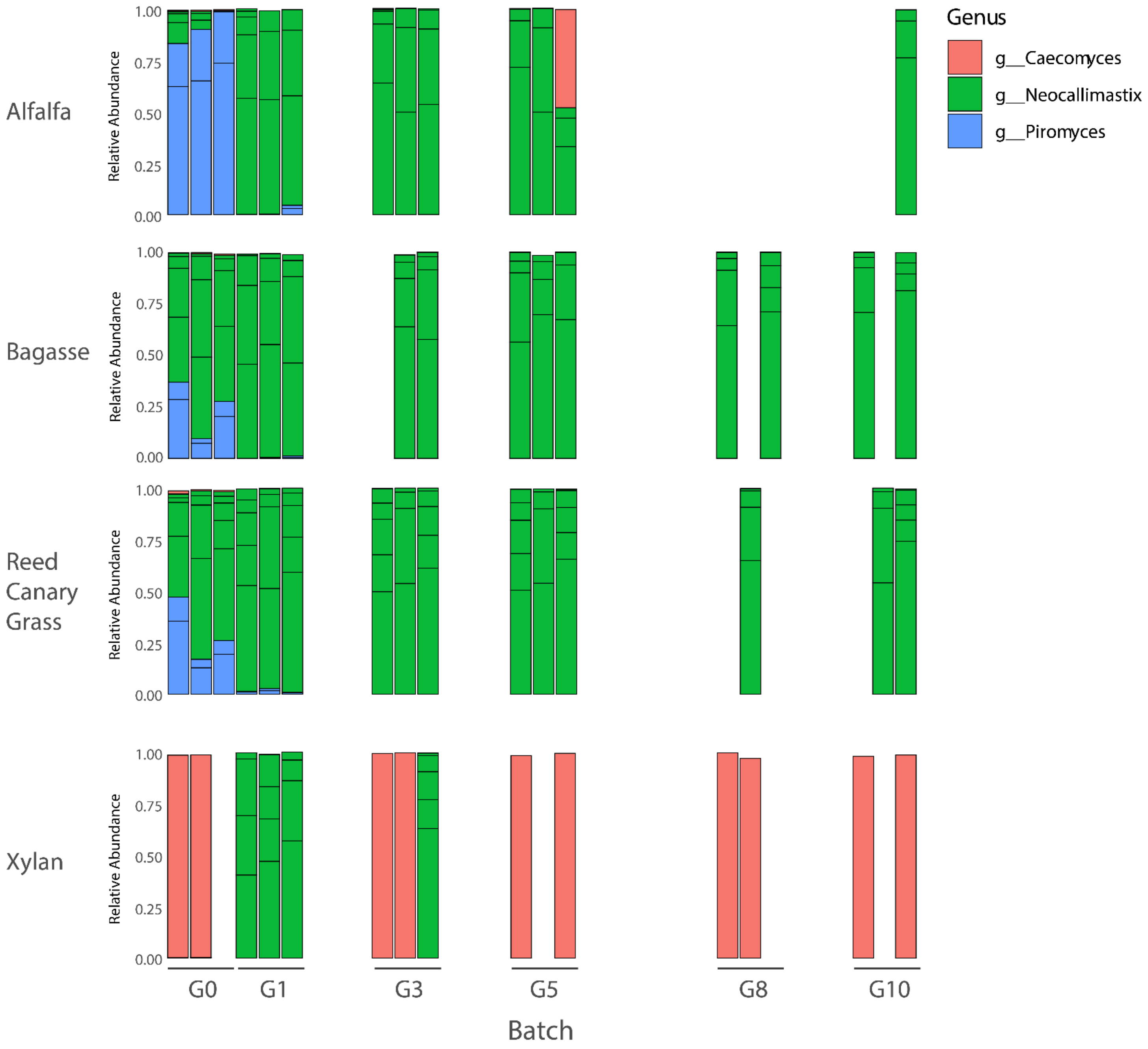

Supplementary Figure 3g. Microbial community composition in consortia treated with chloramphenicol and fecal pellets evaluated by the internal transcribed spacer region 2 (ITS2), as a function of selected batches (G0, G1, G3, G5, G8, and G10) and carbon substrate. The three bars adjacent to each other represent three biological replicates. Because this figure presents the top 20 most abundant amplicon sequence variants (ASVs), the relative abundance in a sample does not necessarily sum to one. Amplicon sequencing reads were processed in R using the package DADA2 version 1.8.0 and the figure was generated in R with the package phyloseq version 1.26.1.

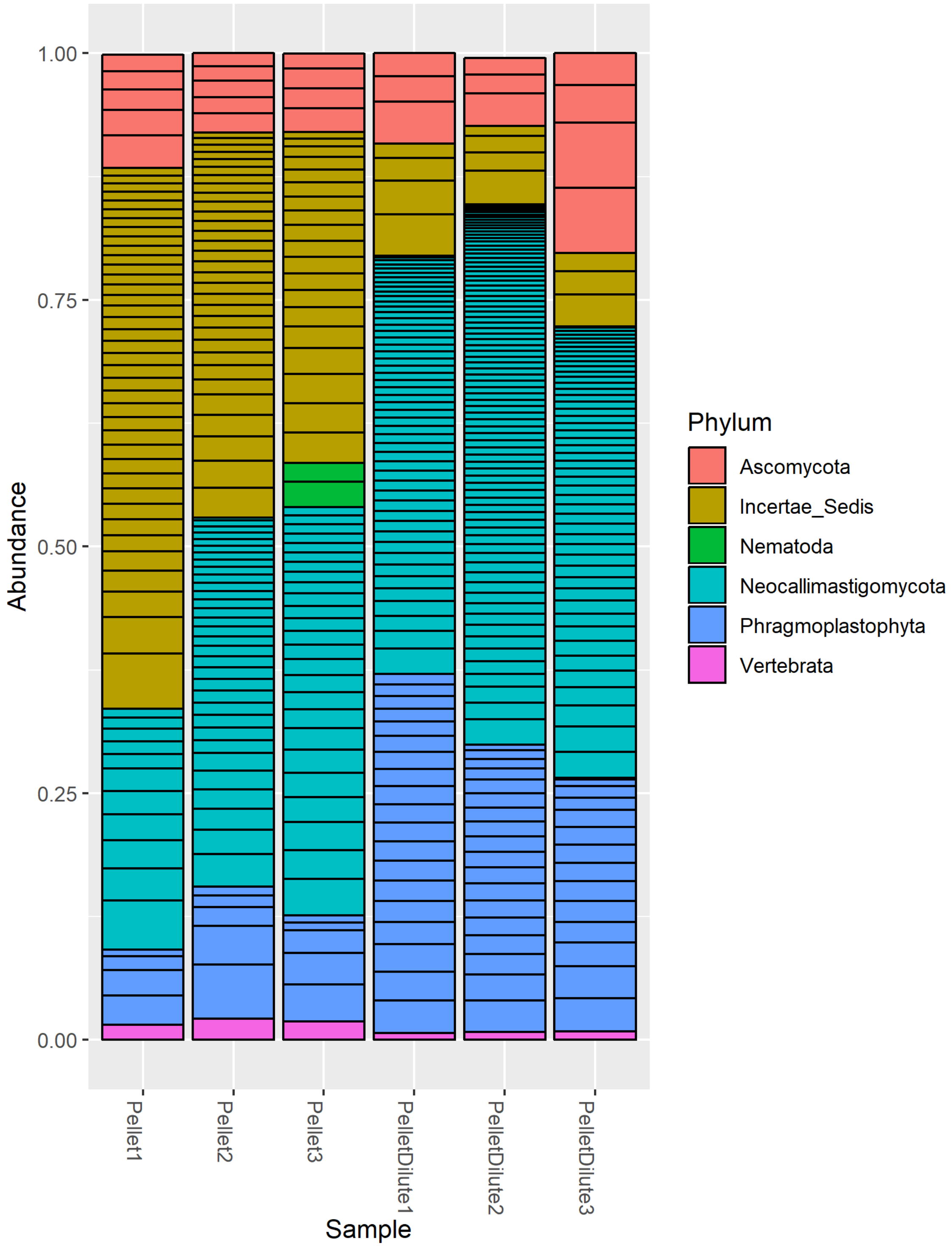

Supplementary Figure 3h. Community composition in fecal pellets at the phylum level evaluated by the V4 region of the 18S rRNA gene. In addition to the three pellet samples, there were also three "pellet_dilute" samples that show the community composition of eight pellets homogenized in culture medium "MC-" (see methods). Amplicon sequencing reads were processed in R using the package DADA2 version 1.8.0 and the figure was generated in R with the package phyloseq version 1.26.1.

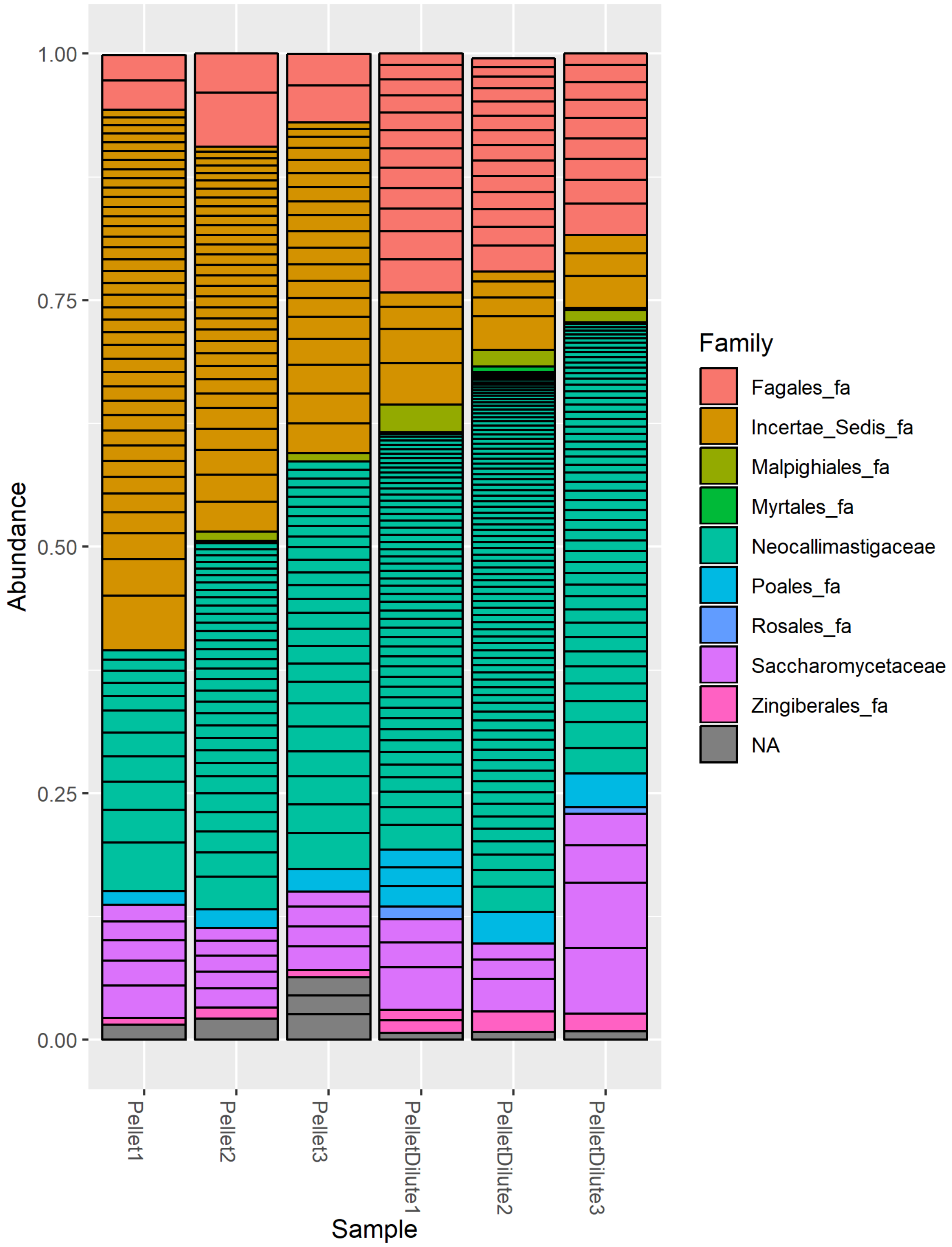

Supplementary Figure 3i. Community composition in fecal pellets at the family level evaluated by the V4 region of the 18S rRNA gene. In addition to the three pellet samples, there were also three "pellet_dilute" samples that show the community composition of eight pellets homogenized in culture medium "MC-" (see methods). The plant taxa were not sequencing artefacts and represented residual plant material in the fecal pellets. Amplicon sequencing reads were processed in R using the package DADA2 version 1.8.0 and the figure was generated in R with the package phyloseq version 1.26.1.

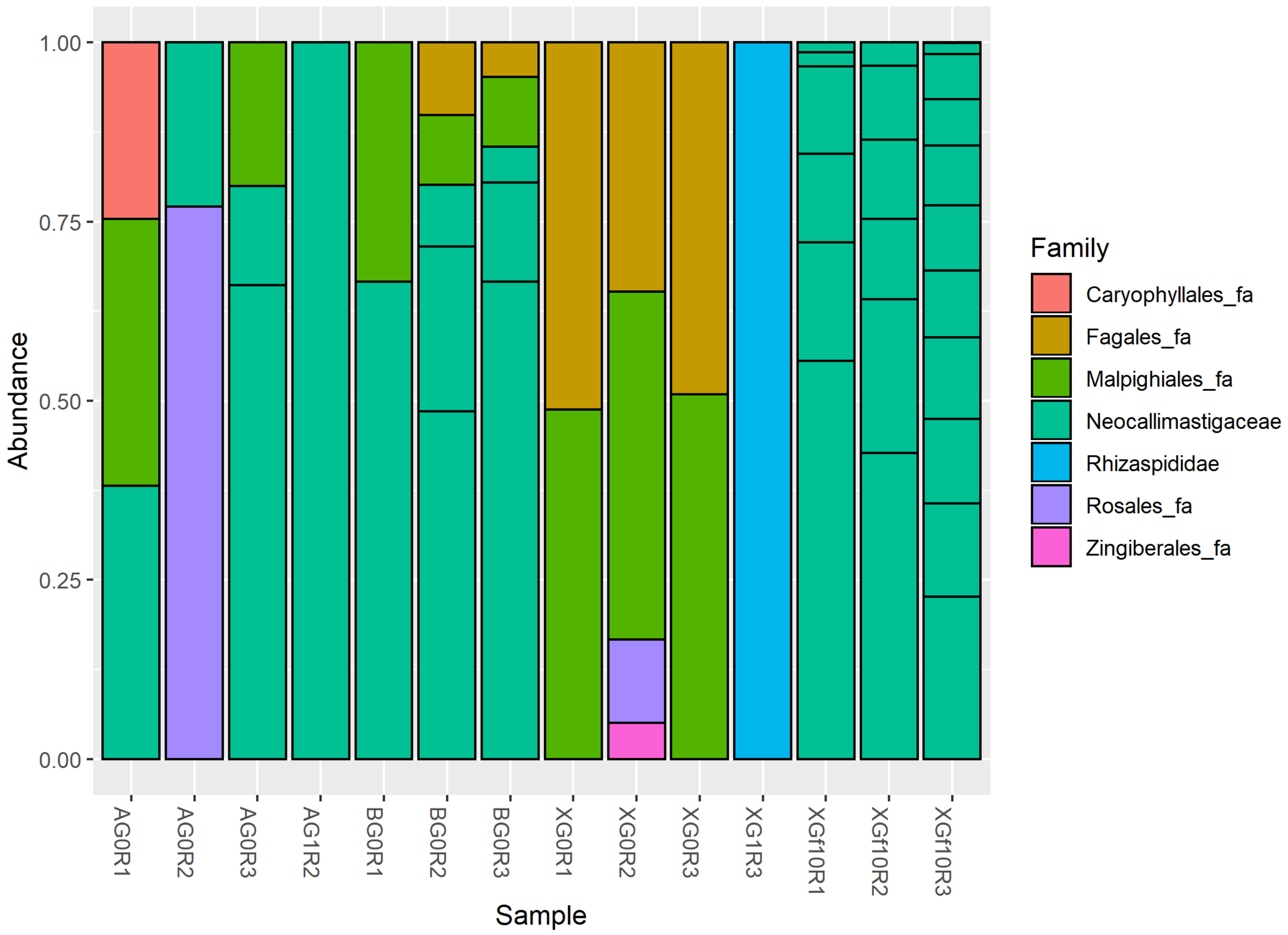

Supplementary Figure 3j. Community composition in antibiotics-free consortia evaluated by the V4 region of the 18S rRNA gene. Amplicon sequencing reads were processed in R using the package DADA2 version 1.8.0 and the figure was generated in R with the package phyloseq version 1.26.1. For each sample from each batch we have provided a unique identifier with the format "SGxRy", where "S" represent the carbon substrate ("A" for alfalfa stems, "B" for bagasse, "R" for reed canary grass, and "X" for xylan), "x" represents the batch number (0 through 10), and "y" represents the replicate number (1, 2, or 3). Note that the only two groups that were not plants were *Neocallimastigaceae* (anaerobic fungi) and *Rhizaspididae* (amoebae). The plant taxa were not sequencing artefacts and represented residual plant material in the fecal pellets.

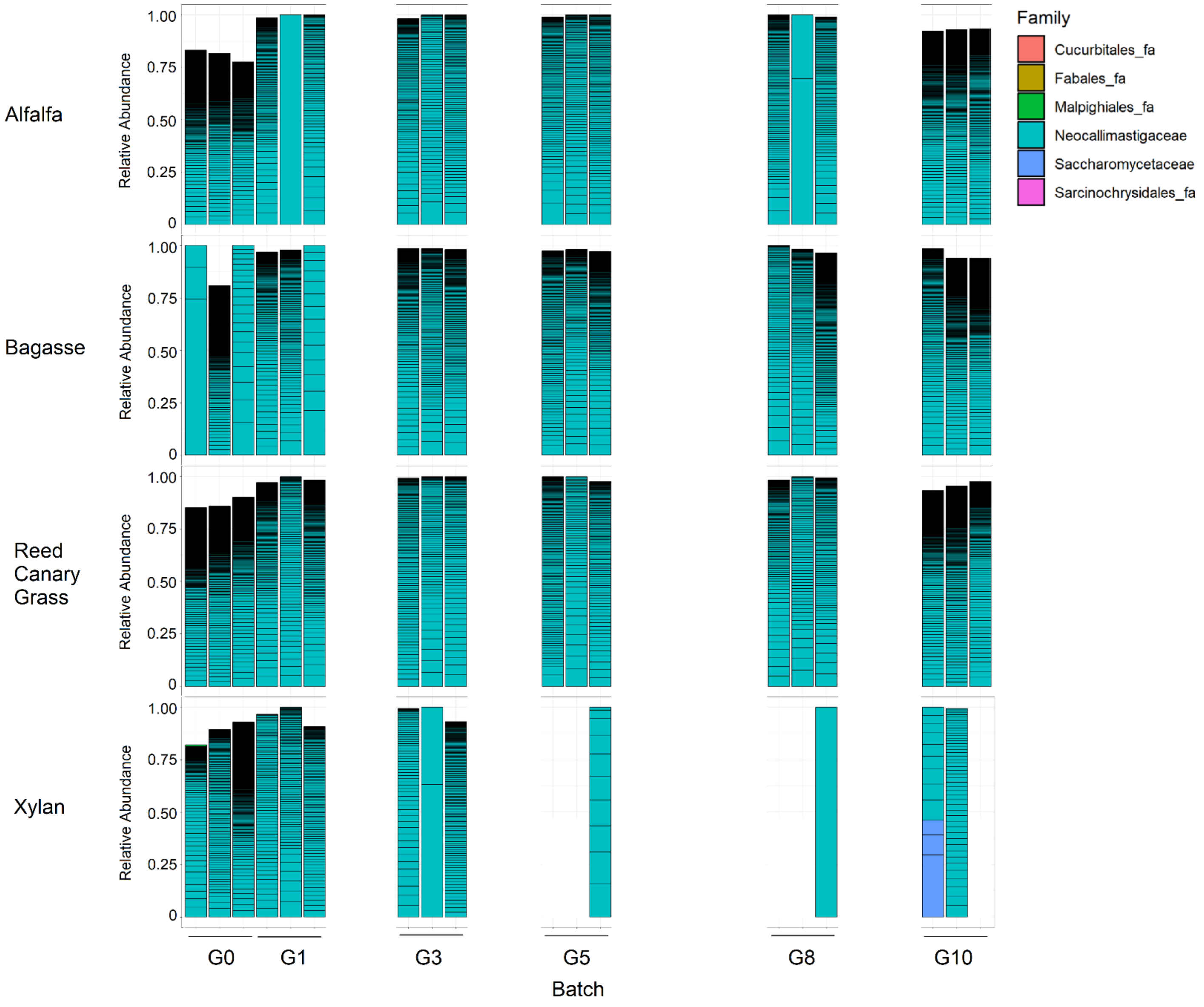

Supplementary Figure 3k. Microbial community composition in consortia treated with penicillin and streptomycin evaluated by the V4 region of the 18S rRNA gene, as a function of selected batches (G0, G1, G3, G5, G8, and G10) and carbon substrate. The three bars adjacent to each other represent three biological replicates. Because this figure presents the top 2000 most abundant amplicon sequence variants (ASVs), the relative abundance in a sample does not necessarily sum to one. Amplicon sequencing reads were processed in R using the package DADA2 version 1.8.0 and the figure was generated in R with the package phyloseq version 1.26.1.

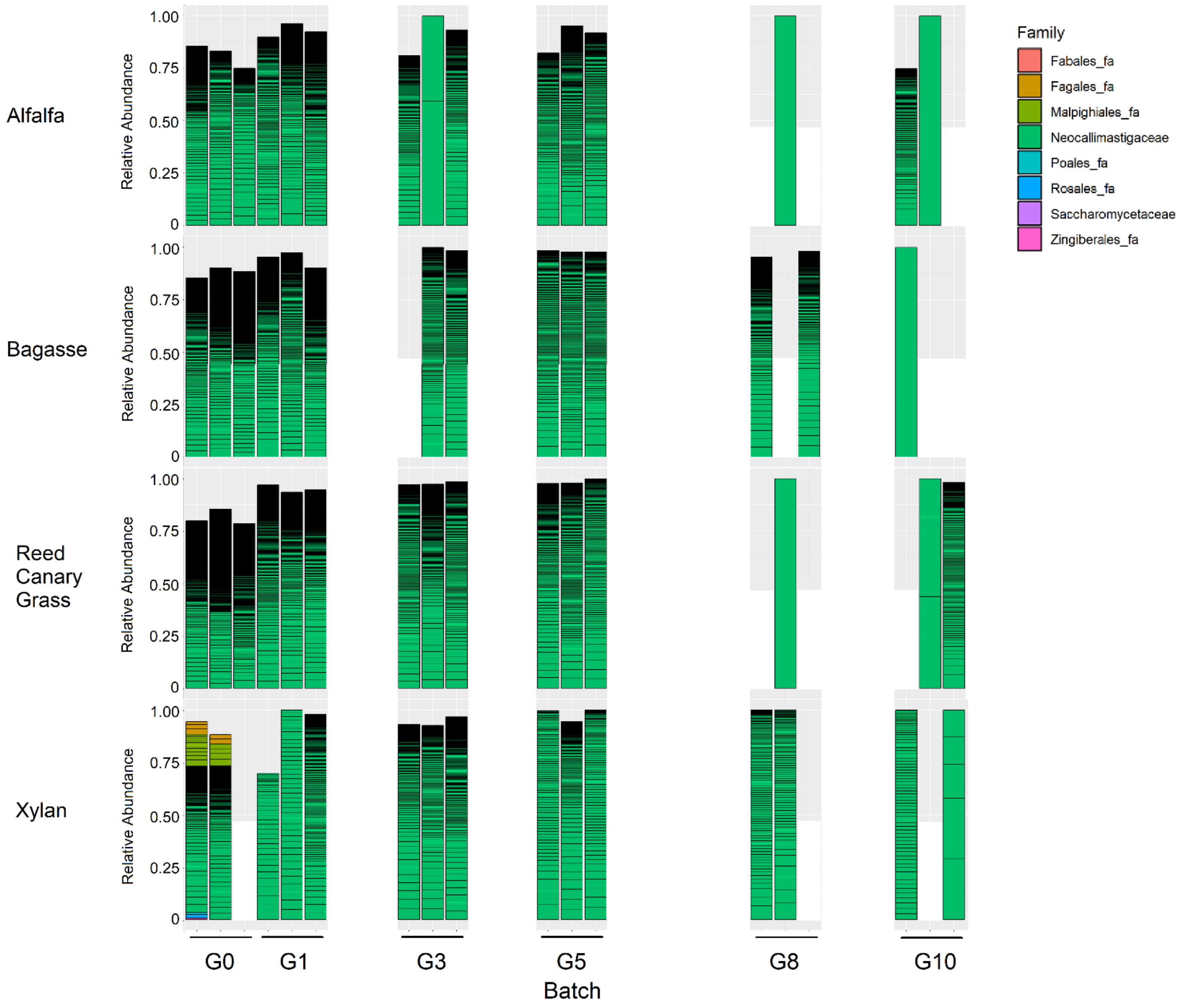

Supplementary Figure 3l. Microbial community composition in consortia treated with chloramphenicol evaluated by the V4 region of the 18S rRNA gene, as a function of selected batches (G0, G1, G3, G5, G8, and G10) and carbon substrate. The three bars adjacent to each other represent three biological replicates. Because this figure presents the top 2000 most abundant amplicon sequence variants (ASVs), the relative abundance in a sample does not necessarily sum to one. Amplicon sequencing reads were processed in R using the package DADA2 version 1.8.0 and the figure was generated in R with the package phyloseq version 1.26.1.

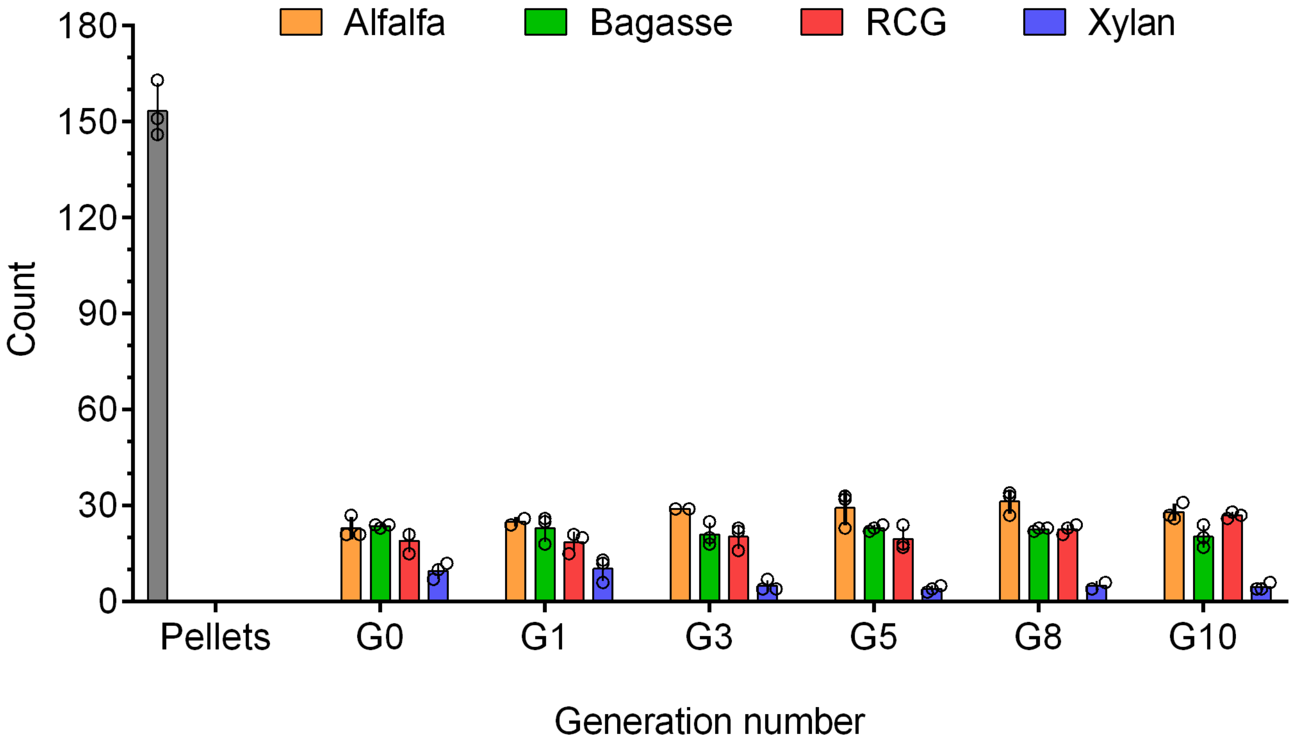

Supplementary Figure 4a. Number of amplicon sequence variant (ASV) clusters evaluated by the V4 region of the 16S rRNA gene (16S-V4) in antibiotics-free consortia, as a function of selected batches (G0, G1, G3, G5, G8, and G10) and carbon substrate. "ASV clusters" were generated by clustering ASVs at the 97% similarity level by the UPARSE algorithm and serve as an operational taxonomic unit at a level between genus and species (see Supplementary Methods for details). The height of the bars represents the mean (n = 3) and the error bars represent standard deviations.

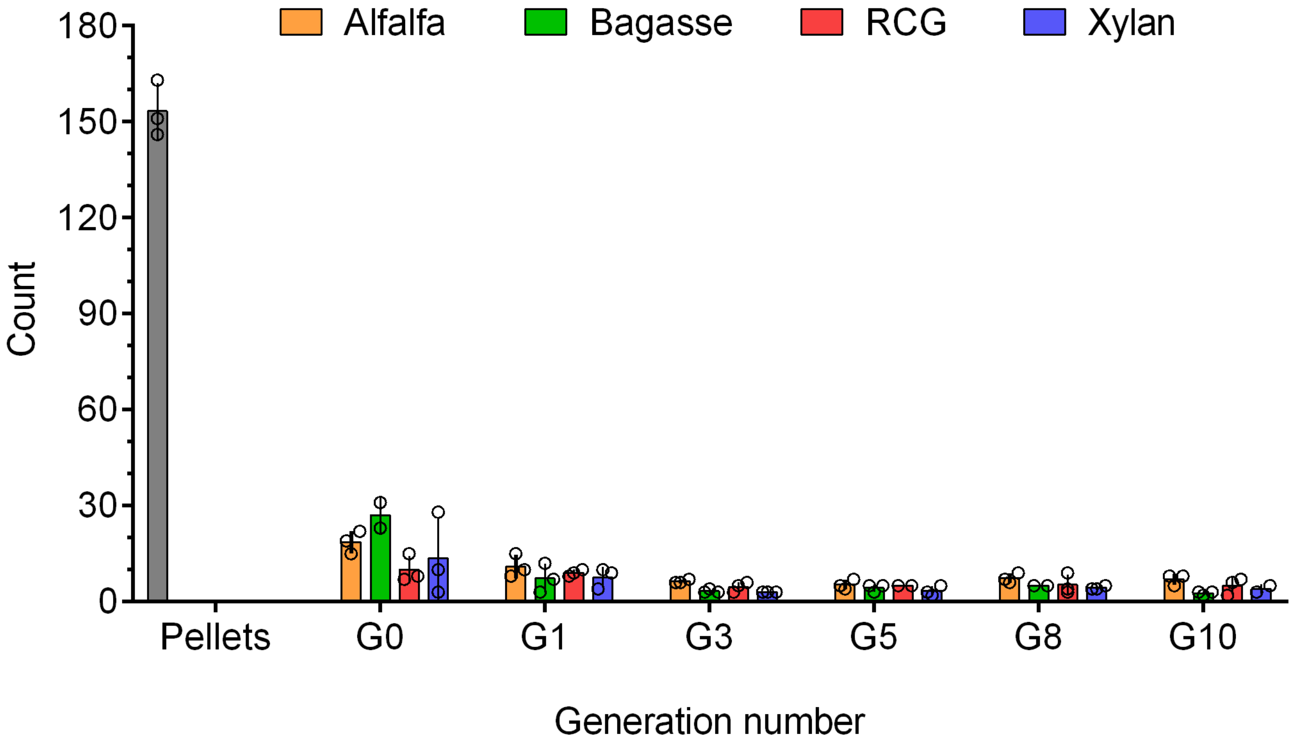

Supplementary Figure 4b. Number of amplicon sequence variant (ASV) clusters evaluated by the V4 region of the 16S rRNA gene (16S-V4) in penicillin and streptomycin-treated (PS) consortia, as a function of selected batches (G0, G1, G3, G5, G8, and G10) and carbon substrate. "ASV clusters" were generated by clustering ASVs at the 97% similarity level by the UPARSE algorithm and serve as an operational taxonomic unit at a level between genus and species (see Supplementary Methods for details). The height of the bars represents the mean (n = 3) and the error bars represent standard deviations.

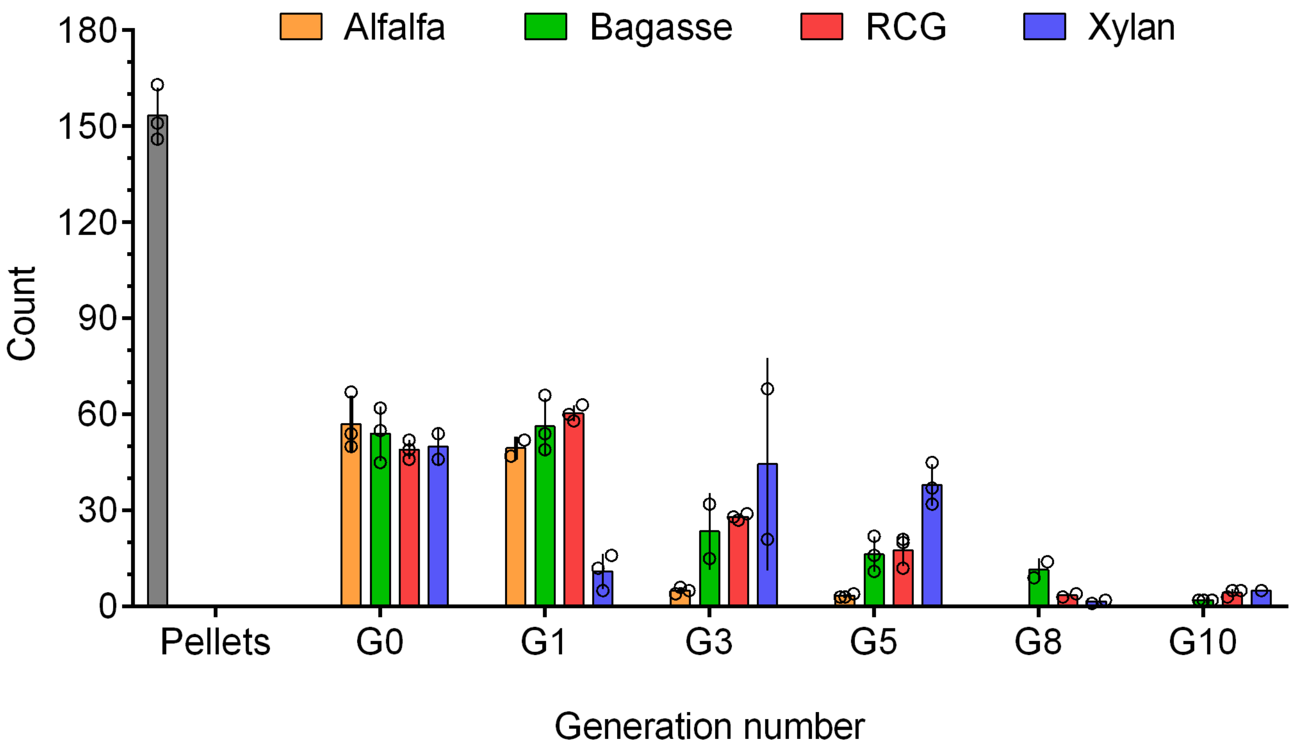

Supplementary Figure 4c. Number of amplicon sequence variant (ASV) clusters evaluated by the V4 region of the 16S rRNA gene (16S-V4) in chloramphenicol-treated (CM) consortia, as a function of selected batches (G0, G1, G3, G5, G8, and G10) and carbon substrate. "ASV clusters" were generated by clustering ASVs at the 97% similarity level by the UPARSE algorithm and serve as an operational taxonomic unit at a level between genus and species (see Supplementary Methods for details). The height of the bars represents the mean (n = 3) and the error bars represent standard deviations.

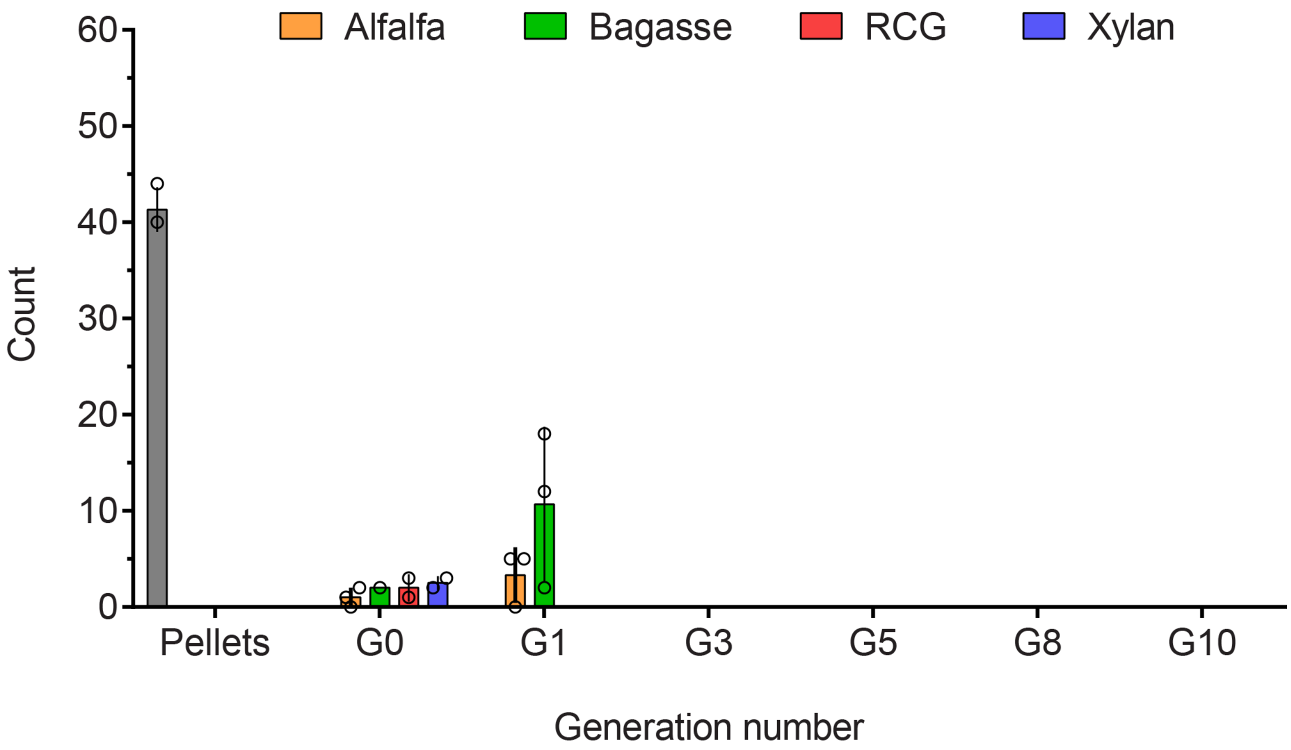

Supplementary Figure 4d. Number of amplicon sequence variant (ASV) clusters evaluated by the internal transcribed spacer region 2 (ITS2) in antibiotics-free consortia, as a function of selected batches (G0, G1, G3, G5, G8, and G10) and carbon substrate. "ASV clusters" were generated by clustering ASVs at the 97% similarity level by the UPARSE algorithm and serve as an operational taxonomic unit at a level between genus and species (see Supplementary Methods for details). The height of the bars represents the mean (n = 3) and the error bars represent standard deviations.

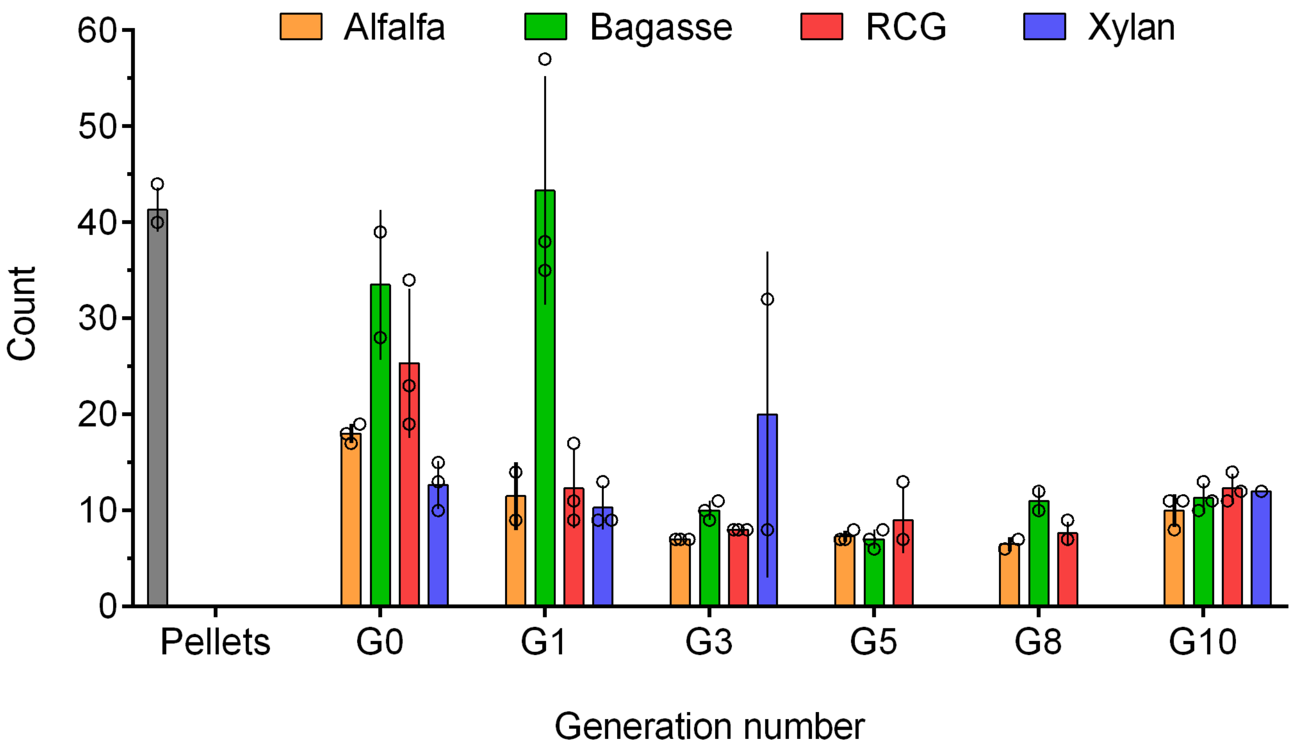

Supplementary Figure 4e. Number of amplicon sequence variant (ASV) clusters evaluated by the internal transcribed spacer region 2 (ITS2) in penicillin and streptomycin-treated (PS) consortia, as a function of selected batches (G0, G1, G3, G5, G8, and G10) and carbon substrate. "ASV clusters" were generated by clustering ASVs at the 97% similarity level by the UPARSE algorithm and serve as an operational taxonomic unit at a level between genus and species (see Supplementary Methods for details). The height of the bars represents the mean (n = 3) and the error bars represent standard deviations.

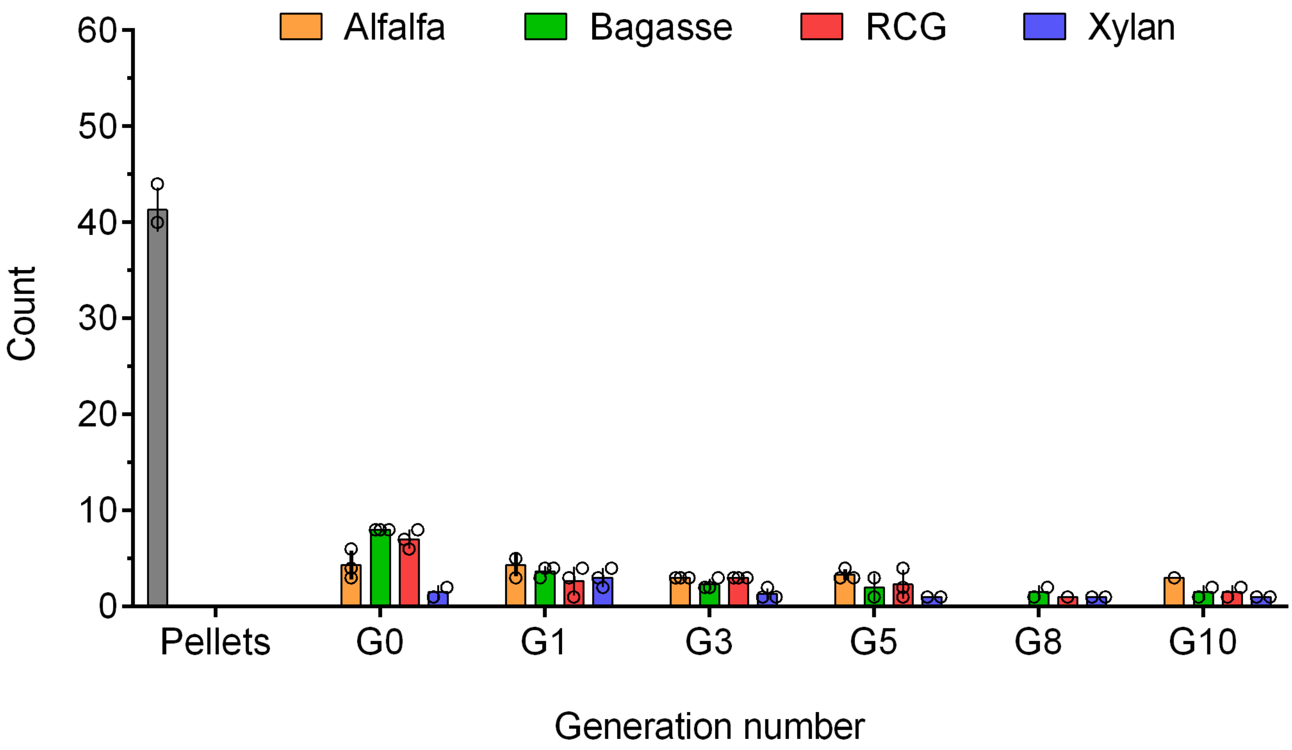

Supplementary Figure 4f. Number of amplicon sequence variant (ASV) clusters evaluated by the internal transcribed spacer region 2 (ITS2) in chloramphenicol-treated (CM) consortia, as a function of selected batches (G0, G1, G3, G5, G8, and G10) and carbon substrate. "ASV clusters" were generated by clustering ASVs at the 97% similarity level by the UPARSE algorithm and serve as an operational taxonomic unit at a level between genus and species (see Supplementary Methods for details). The height of the bars represents the mean (n = 3) and the error bars represent standard deviations.

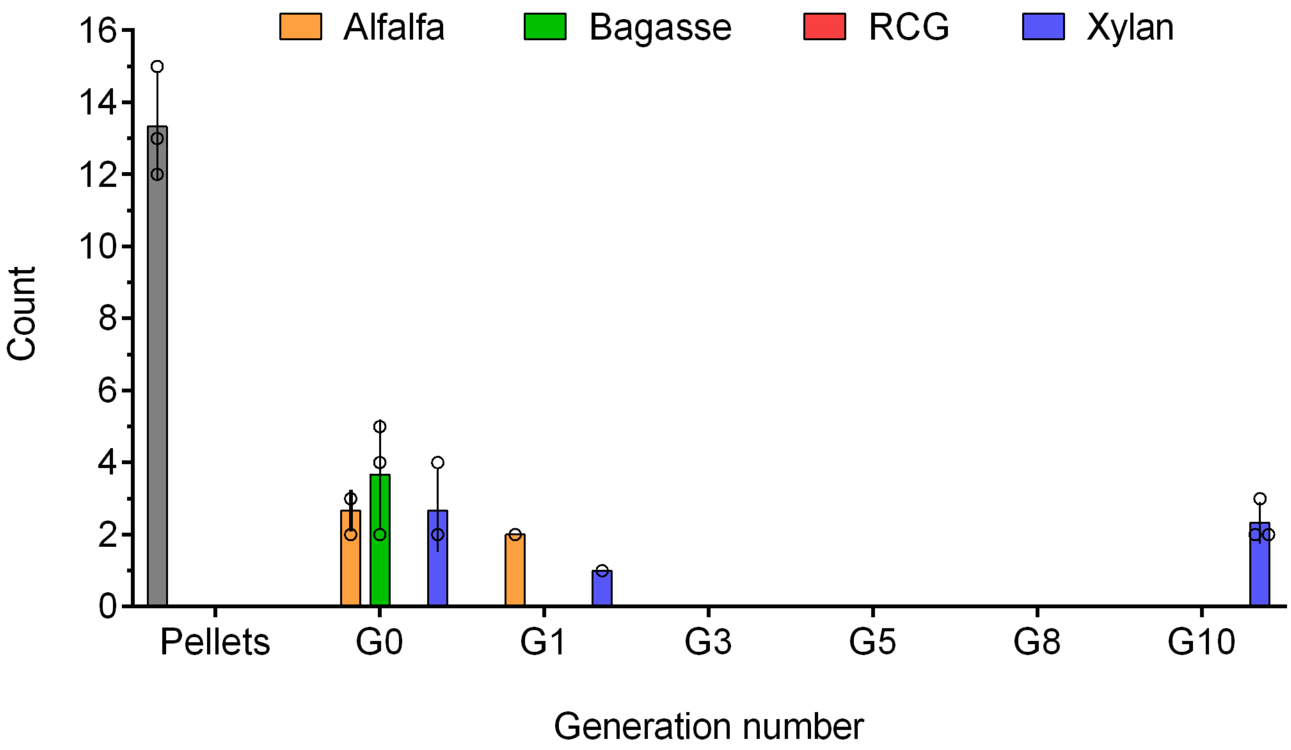

Supplementary Figure 4g. Number of amplicon sequence variant (ASV) clusters evaluated by the V4 region of the 18S rRNA gene (18S-V4) in antibiotics-free consortia, as a function of selected batches (G0, G1, G3, G5, G8, and G10) and carbon substrate. "ASV clusters" were generated by clustering ASVs at the 97% similarity level by the UPARSE algorithm and serve as an operational taxonomic unit at a level between genus and species (see Supplementary Methods for details). The height of the bars represents the mean (n = 3) and the error bars represent standard deviations.

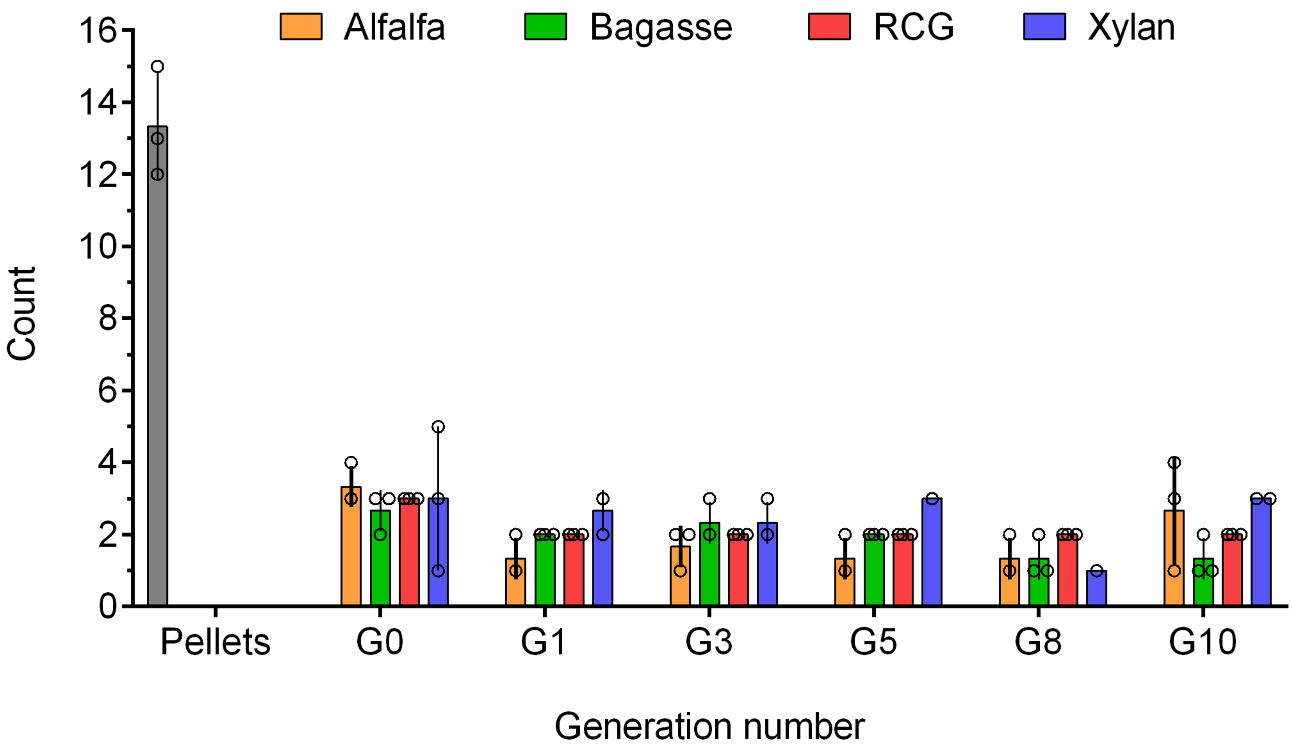

Supplementary Figure 4h. Number of amplicon sequence variant (ASV) clusters evaluated by the V4 region of the 18S rRNA gene (18S-V4) in penicillin and streptomycin-treated (PS) consortia, as a function of selected batches (G0, G1, G3, G5, G8, and G10) and carbon substrate. "ASV clusters" were generated by clustering ASVs at the 97% similarity level by the UPARSE algorithm and serve as an operational taxonomic unit at a level between genus and species (see Supplementary Methods for details). The height of the bars represents the mean (n = 3) and the error bars represent standard deviations.

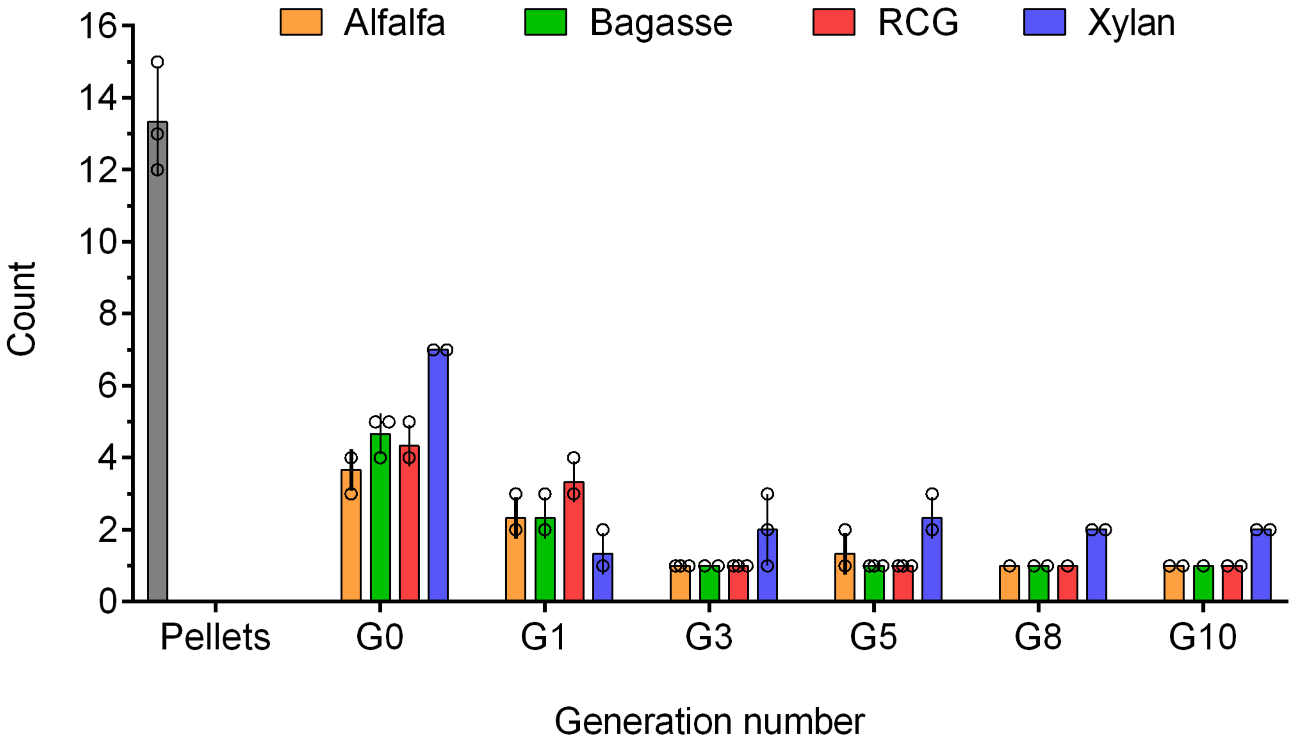

Supplementary Figure 4i. Number of amplicon sequence variant (ASV) clusters evaluated by the V4 region of the 18S rRNA gene (18S-V4) in chloramphenicol-treated (CM) consortia, as a function of selected batches (G0, G1, G3, G5, G8, and G10) and carbon substrate. "ASV clusters" were generated by clustering ASVs at the 97% similarity level by the UPARSE algorithm and serve as an operational taxonomic unit at a level between genus and species (see Supplementary Methods for details). The height of the bars represents the mean (n = 3) and the error bars represent standard deviations.

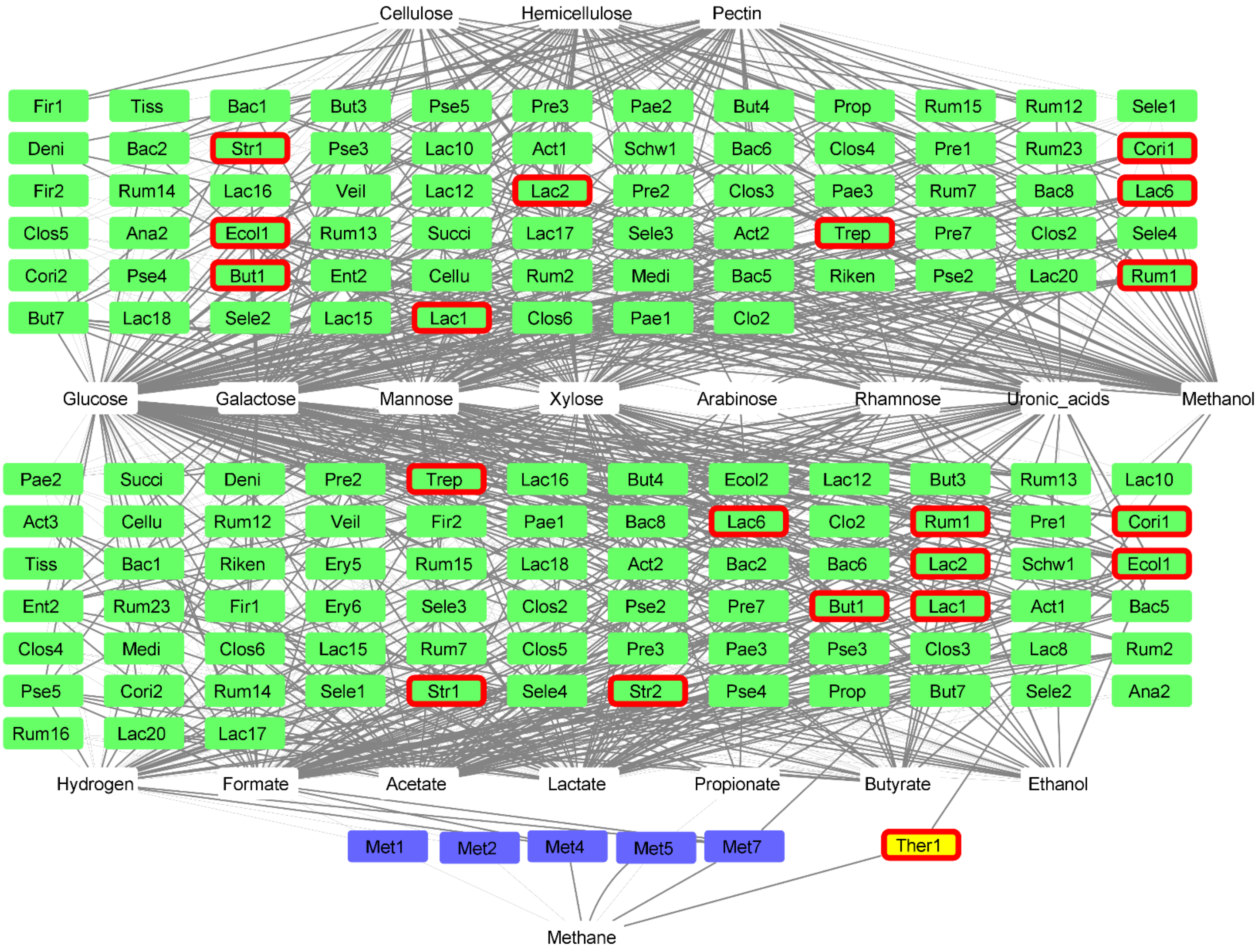

Supplementary Figure 5a. Carbon cross-feeding between microorganisms in the consortium AG10R3 (grown on alfalfa stems, batch 10, replicate 3). Each rectangular shape containing a three-to-five-letter acronym represents a metagenome-assembled genome (MAG, see Supplementary Data 4 for list of acronyms). The thickness of the lines is scaled with the relative abundance of the connected MAG in the corresponding consortium. A line is connected between a MAG and a metabolite if the pathway responsible for the utilization/production of the metabolite is at least 75% complete in the MAG. The MAGs with > 1% relative abundance in the metagenome are highlighted with red boxes and the other MAGs are considered rare. In this consortium the additional metabolic potentials offered by rare MAGs include methanogenesis from hydrogen and formate. None of the rare bacterial MAGs provide any metabolic potentials in addition to the MAGs with 1% relative abundance.

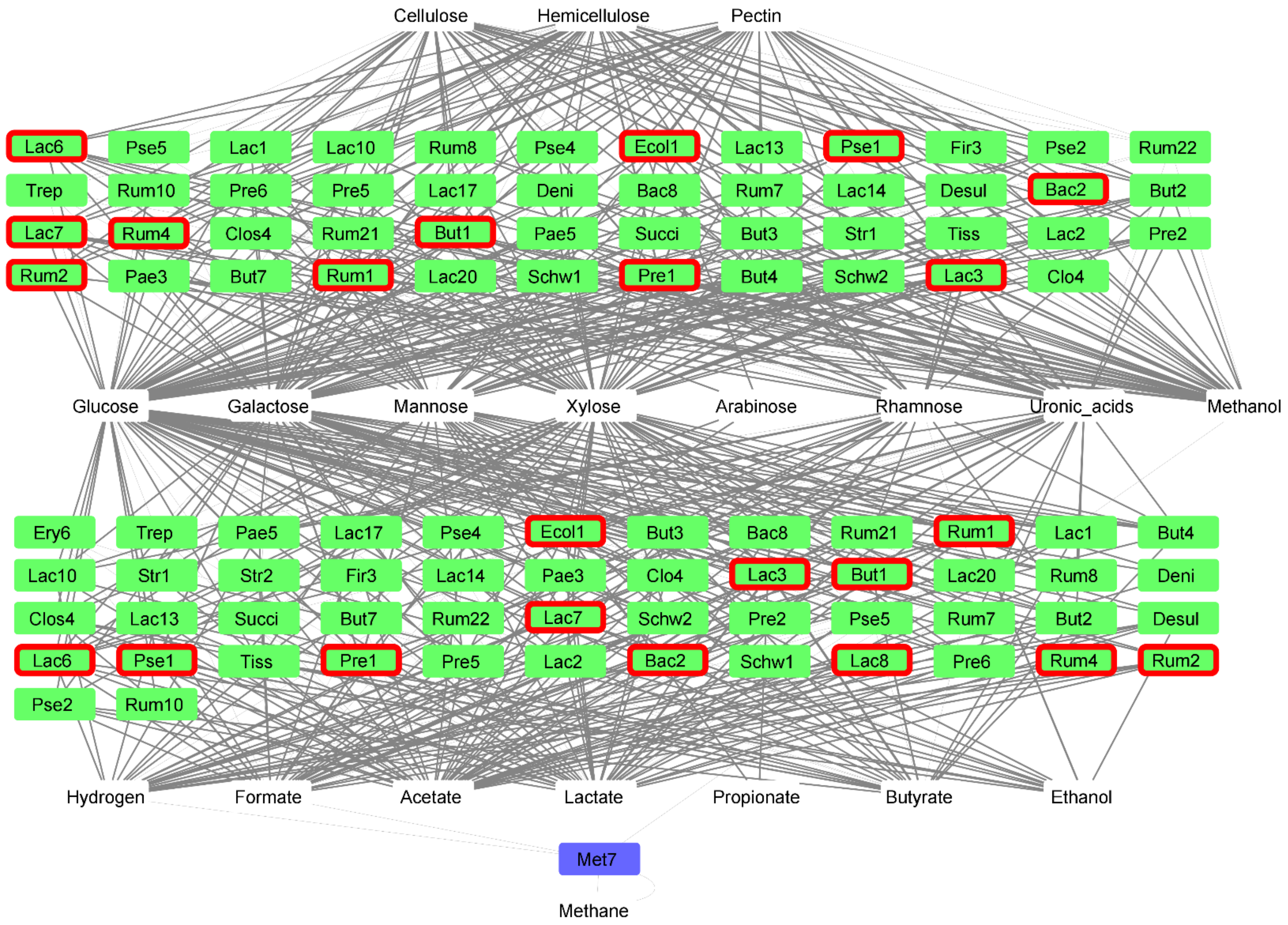

Supplementary Figure 5b. Carbon cross-feeding between microorganisms in the consortium BG10R2 (grown on bagasse, batch 10, replicate 2). Each rectangular shape containing a three-to-five-letter acronym represents a metagenome-assembled genome (MAG, see Supplementary Data 4 for list of acronyms). The thickness of the lines is scaled with the relative abundance of the connected MAG in the corresponding consortium. A line is connected between a MAG and a metabolite if the pathway responsible for the utilization/production of the metabolite is at least 75% complete in the MAG. The MAGs with > 1% relative abundance in the metagenome are highlighted with red boxes and the other MAGs are considered rare. In this consortium the additional metabolic potential provided by a rare MAG (“Met7”) is methanogenesis. None of the rare bacterial MAGs provide any metabolic potentials in addition to the MAGs with 1% relative abundance.

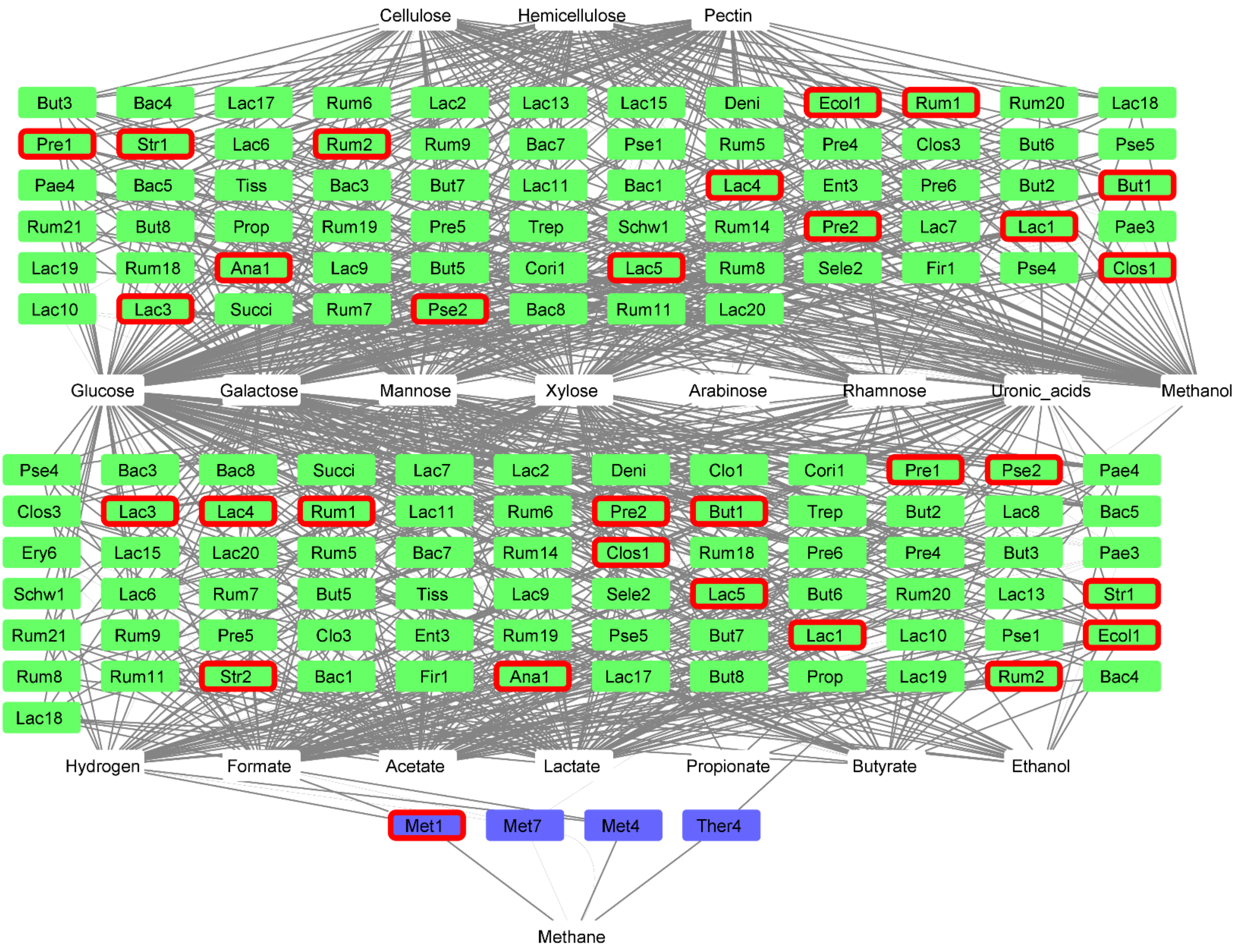

Supplementary Figure 5c. Carbon cross-feeding between microorganisms in the consortium RG10R3 (grown on reed canary grass, batch 10, replicate 3). Each rectangular shape containing a three-to-five-letter acronym represents a metagenome-assembled genome (MAG, see Supplementary Data 4 for list of acronyms). The thickness of the lines is scaled with the relative abundance of the connected MAG in the corresponding consortium. A line is connected between a MAG and a metabolite if the pathway responsible for the utilization/production of the metabolite is at least 75% complete in the MAG. The MAGs with > 1% relative abundance in the metagenome are highlighted with red boxes and the other MAGs are considered rare. In this consortium the additional metabolic potential provided by a rare MAG ("Ther4") is methanogenesis from methanol. None of the rare bacterial MAGs provide any metabolic potentials in addition to the MAGs with 1% relative abundance.

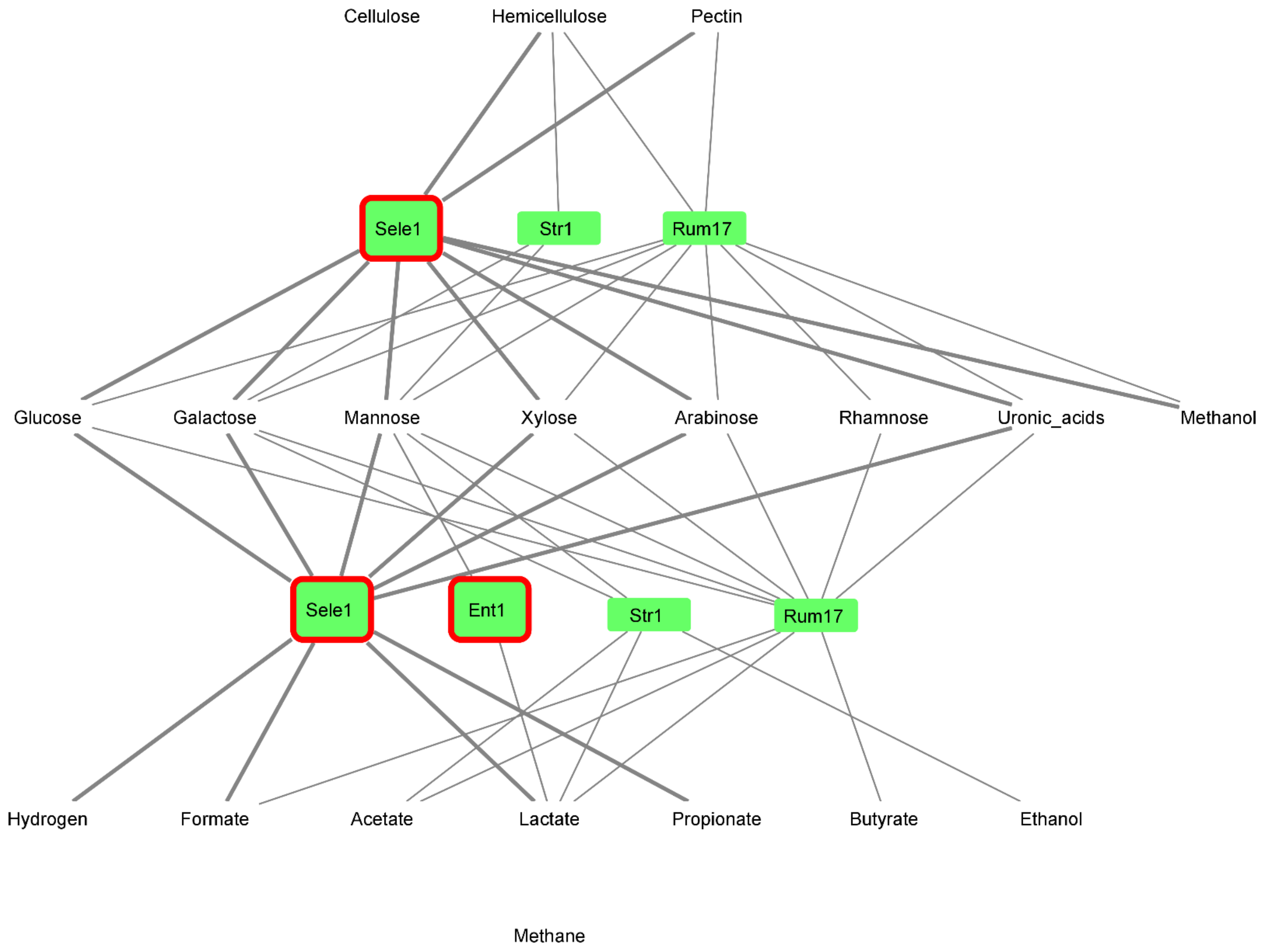

Supplementary Figure 5d. Carbon cross-feeding between microorganisms in the consortium XG10R1 (grown on xylan, batch 10, replicate 1). Each rectangular shape containing a three-to-five-letter acronym represents a metagenome-assembled genome (MAG, see Supplementary Data 4 for list of acronyms). The thickness of the lines is scaled with the relative abundance of the connected MAG in the corresponding consortium. A line is connected between a MAG and a metabolite if the pathway responsible for the utilization/production of the metabolite is at least 75% complete in the MAG. The MAGs with > 1% relative abundance in the metagenome are highlighted with red boxes and the other MAGs are considered rare. In this consortium the additional metabolic potential provided by rare MAGs ("Str1" and "Rum17") is the production of butyrate and ethanol.

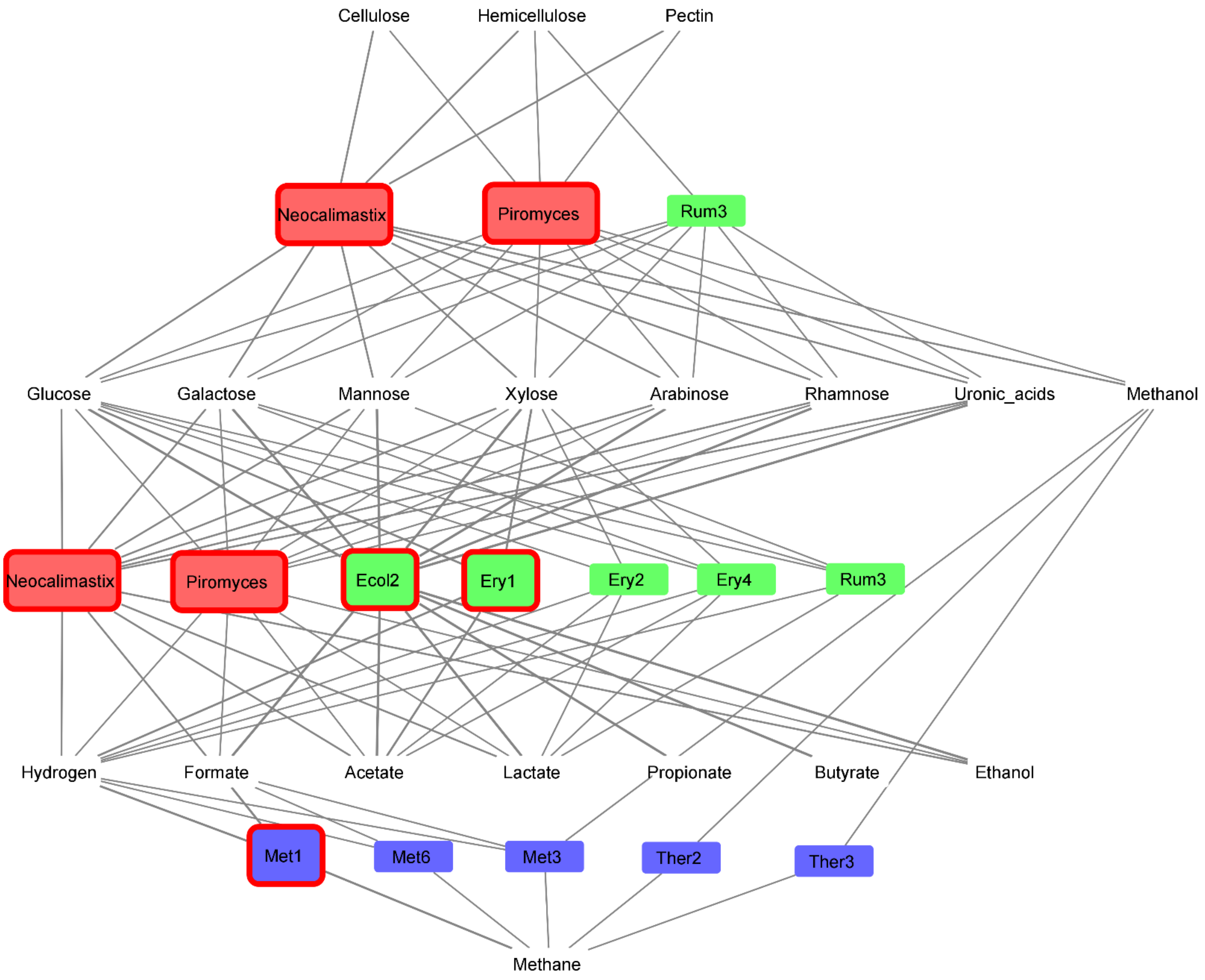

Supplementary Figure 5e. Carbon cross-feeding between microorganisms in the consortium AG10R1-PS (grown on alfalfa stems and treated with penicillin and streptomycin, batch 10, replicate 1). Each rectangular shape containing a three-to-five-letter acronym represents a metagenome-assembled genome (MAG, see Supplementary Data 4 for list of acronyms). The thickness of the lines is scaled with the relative abundance of the connected MAG in the corresponding consortium. A line is connected between a MAG and a metabolite if the pathway responsible for the utilization/production of the metabolite is at least 75% complete in the MAG. The MAGs with > 1% relative abundance in the metagenome are highlighted with red boxes and the other MAGs are considered rare. In this consortium the additional metabolic potential provided by rare MAGs ("Met3", "Ther2", and "Ther3") is methanogenesis from methanol.

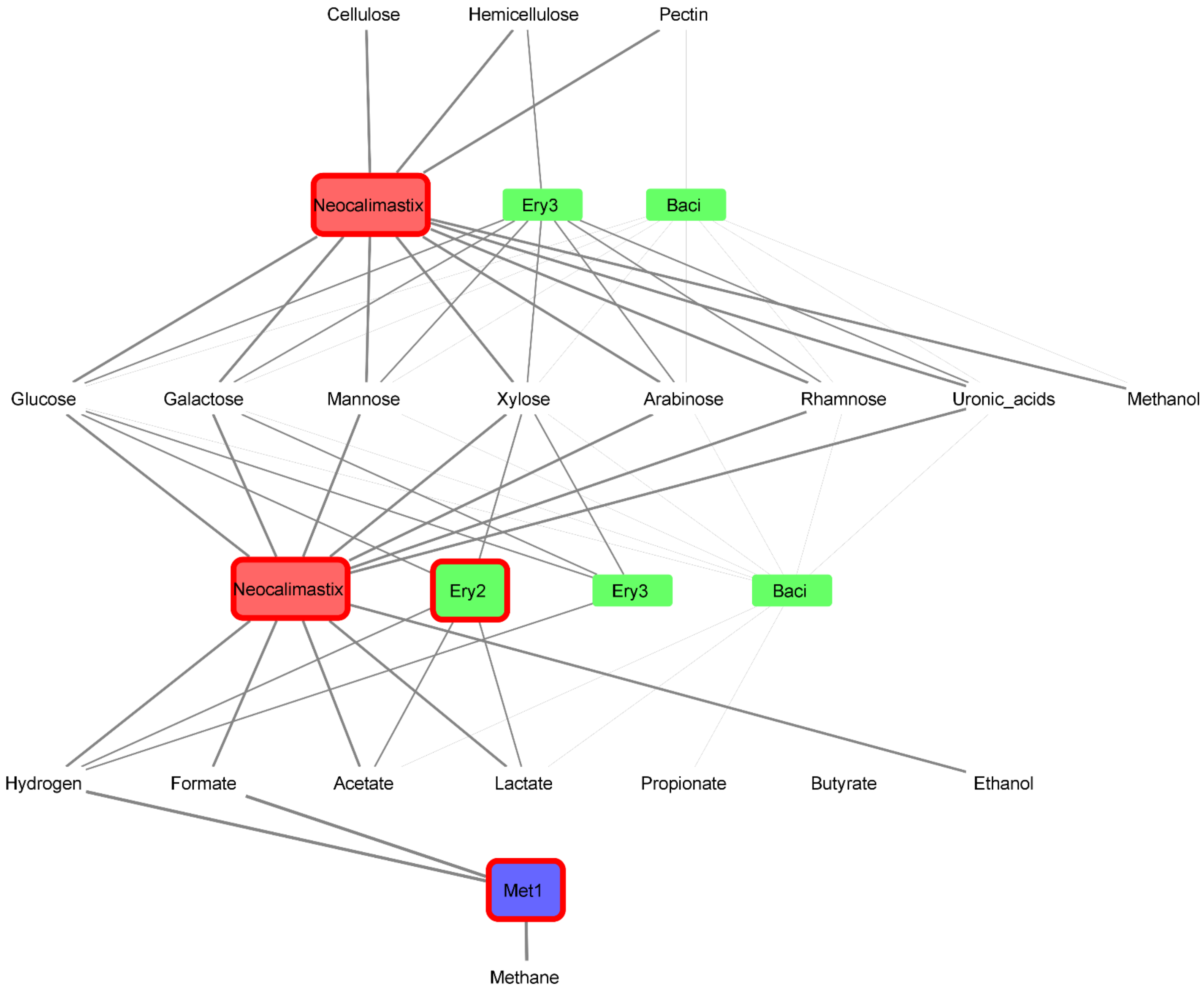

Supplementary Figure 5f. Carbon cross-feeding between microorganisms in the consortium BG10R3-PS (grown on bagasse and treated with penicillin and streptomycin, batch 10, replicate 3). Each rectangular shape containing a three-to-five-letter acronym represents a metagenome-assembled genome (MAG, see Supplementary Data 4 for list of acronyms). The thickness of the lines is scaled with the relative abundance of the connected MAG in the corresponding consortium. A line is connected between a MAG and a metabolite if the pathway responsible for the utilization/production of the metabolite is at least 75% complete in the MAG. The MAGs with > 1% relative abundance in the metagenome are highlighted with red boxes and the other MAGs are considered rare. In this consortium the additional metabolic potential provided by a rare MAG ("Baci") is propionate production.

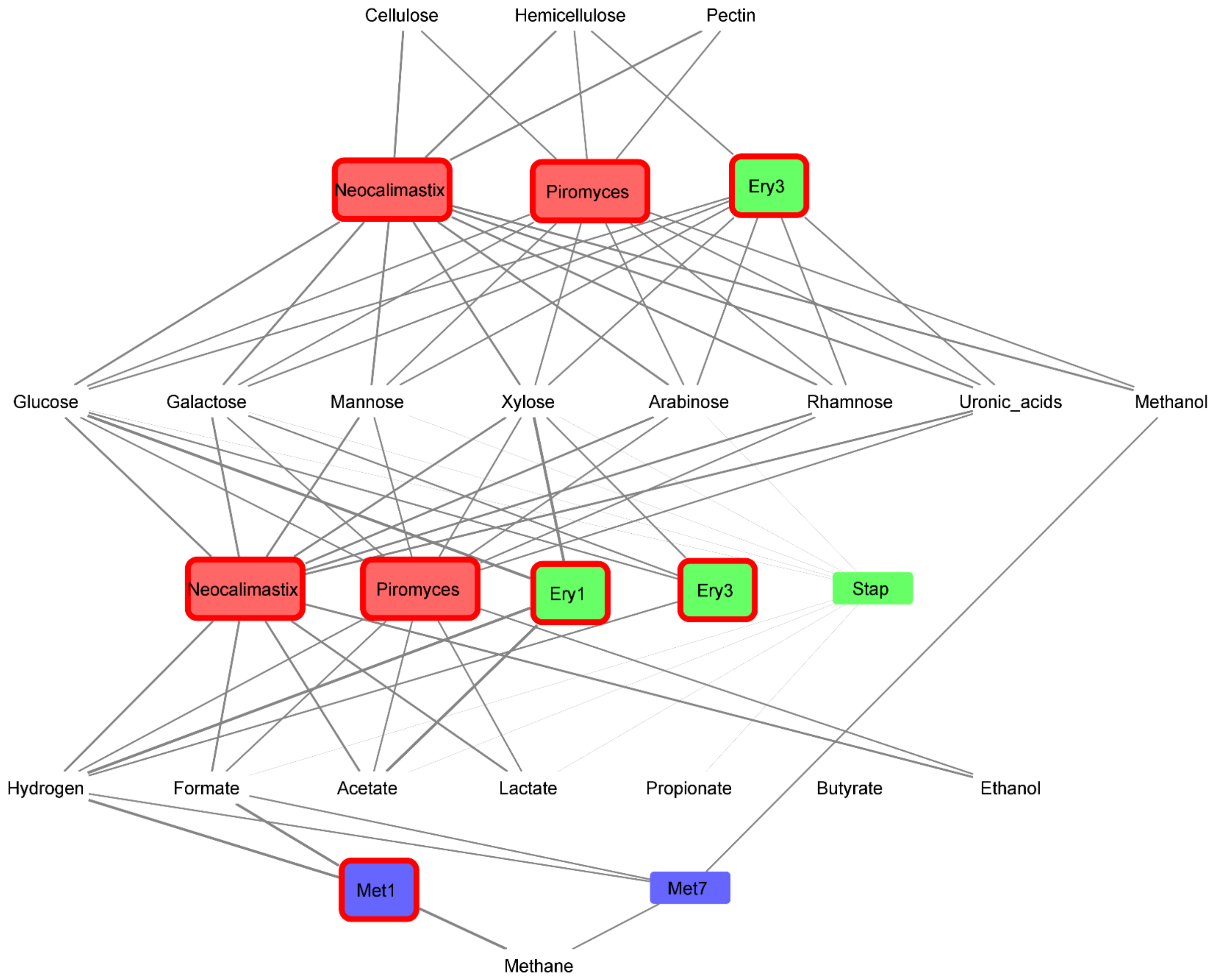

Supplementary Figure 5g. Carbon cross-feeding between microorganisms in the consortium RG10R3-PS (grown on reed canary grass and treated with penicillin and streptomycin, batch 10, replicate 3). Each rectangular shape containing a three-to-five-letter acronym represents a metagenome-assembled genome (MAG, see Supplementary Data 4 for list of acronyms). The thickness of the lines is scaled with the relative abundance of the connected MAG in the corresponding consortium. A line is connected between a MAG and a metabolite if the pathway responsible for the utilization/production of the metabolite is at least 75% complete in the MAG. The MAGs with > 1% relative abundance in the metagenome are highlighted with red boxes and the other MAGs are considered rare. In this consortium the additional metabolic potential provided by rare MAGs (“Stap” and “Met7”) include propionate production and methanogenesis from methanol.

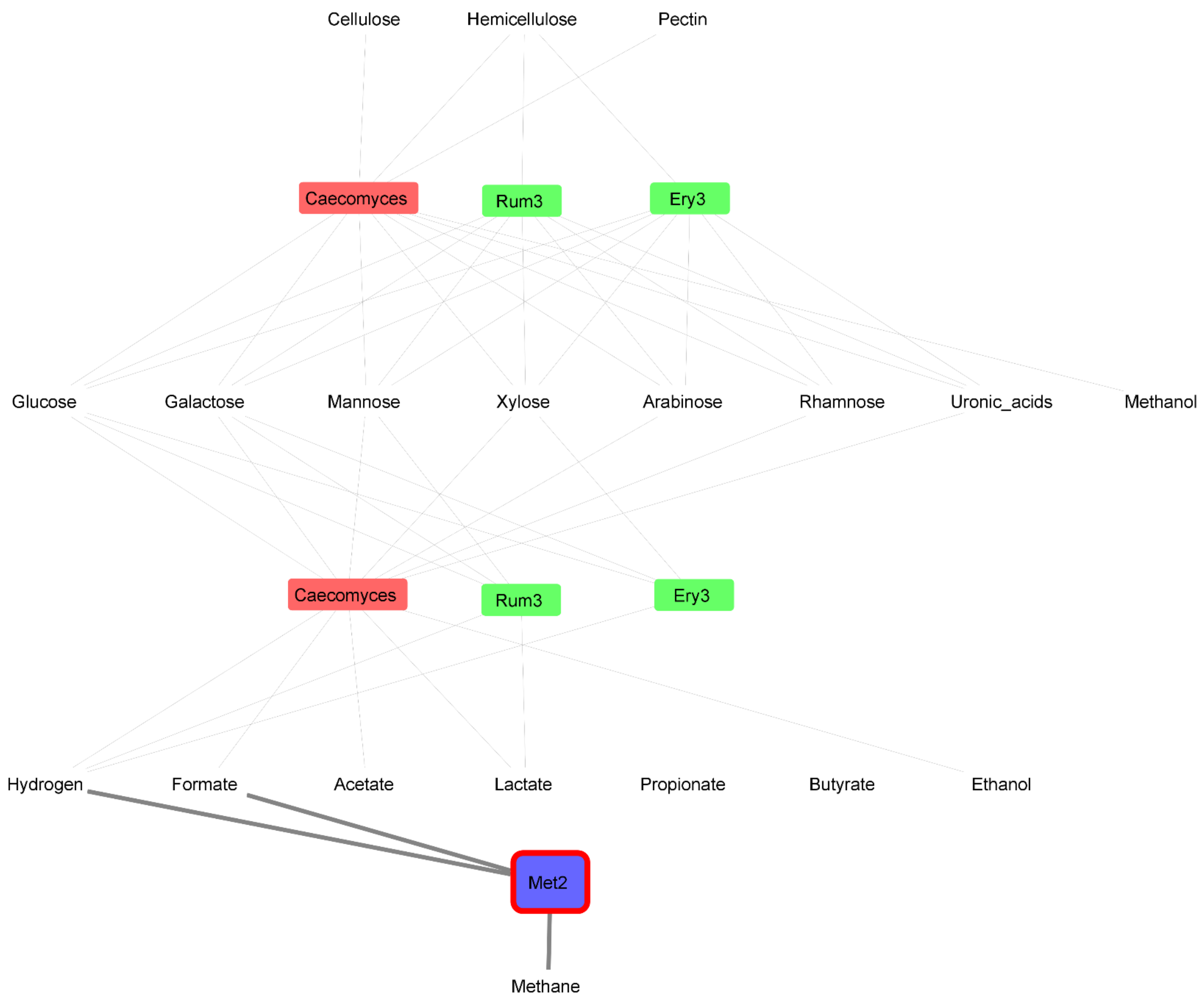

Supplementary Figure 5h. Carbon cross-feeding between microorganisms in the consortium XG10R3-PS (grown on xylan and treated with penicillin and streptomycin, batch 10, replicate 3). Each rectangular shape containing a three-to-five-letter acronym represents a metagenome-assembled genome (MAG, see Supplementary Data 4 for list of acronyms). The thickness of the lines is scaled with the relative abundance of the connected MAG in the corresponding consortium. A line is connected between a MAG and a metabolite if the pathway responsible for the utilization/production of the metabolite is at least 75% complete in the MAG. The MAG with > 1% relative abundance in the metagenome is highlighted with red boxes and the other MAGs are considered rare. In this consortium all hydrolytic and fermentative pathways are found in rare members.

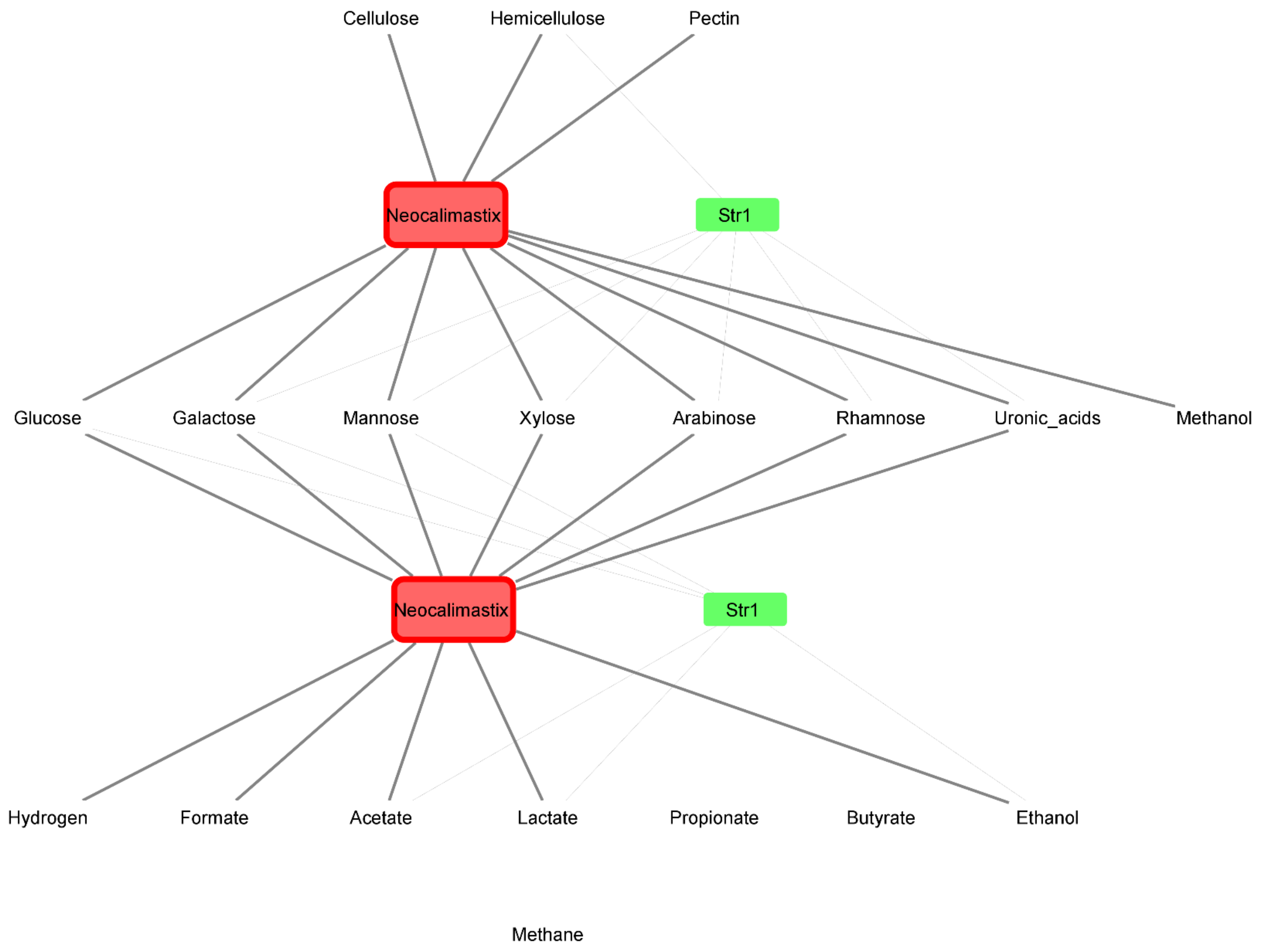

Supplementary Figure 5i. Carbon cross-feeding between microorganisms in the consortium AG10R2-CM (grown on alfalfa stems and treated with chloramphenicol, batch 10, replicate 2). Each rectangular shape containing a three-to-five-letter acronym represents a metagenome-assembled genome (MAG, see Supplementary Data 4 for list of acronyms). The thickness of the lines is scaled with the relative abundance of the connected MAG in the corresponding consortium. A line is connected between a MAG and a metabolite if the pathway responsible for the utilization/production of the metabolite is at least 75% complete in the MAG. The dominant member (> 1% relative abundance) in the metagenome is highlighted with red boxes and the MAG is considered rare. The rare bacterial MAG ("Str1") provides no metabolic potential additional to *Neocallimastix*.

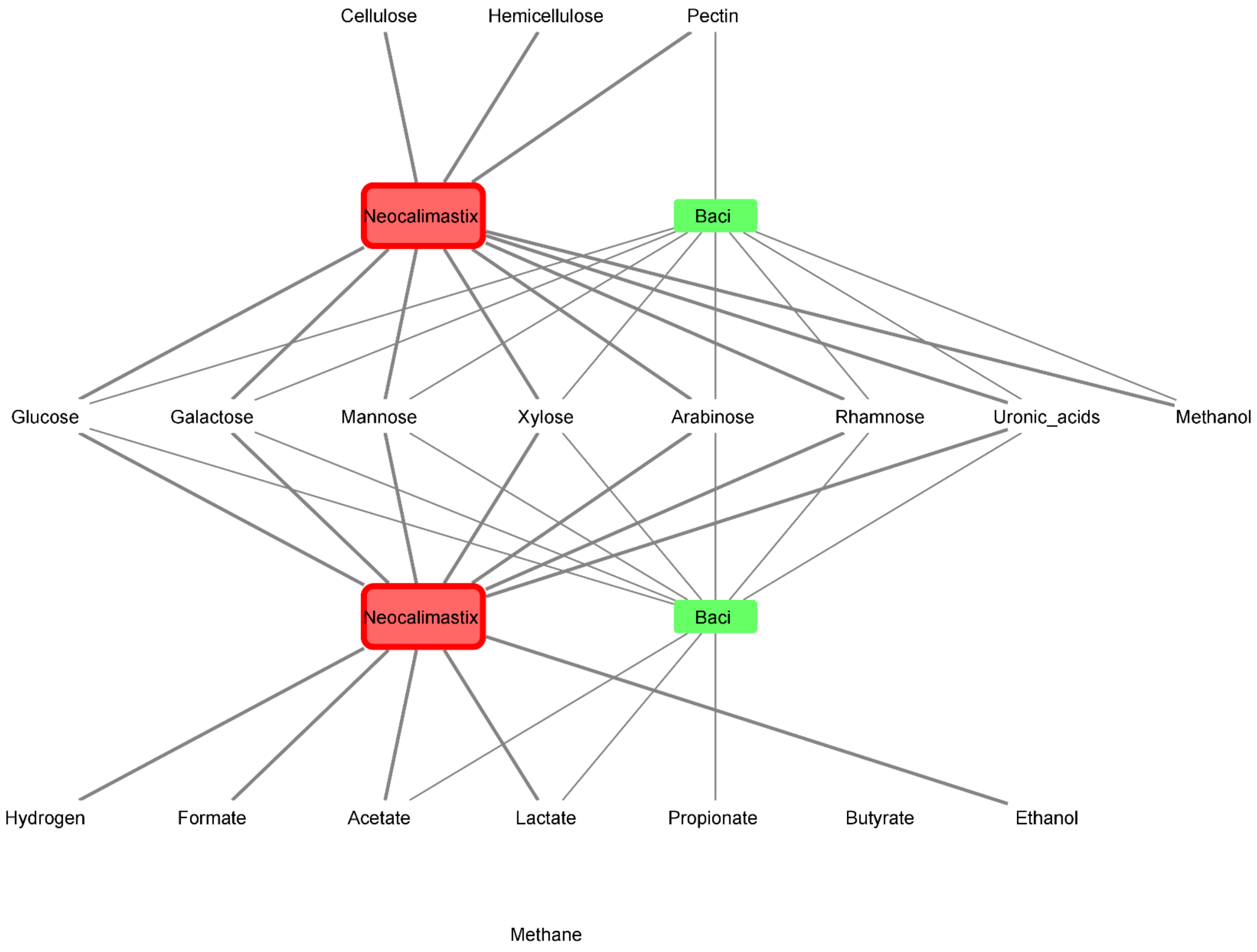

Supplementary Figure 5j. Carbon cross-feeding between microorganisms in the consortium BG10R2-CM (grown on bagasse and treated with chloramphenicol, batch 10, replicate 2). Each rectangular shape containing a three-to-five-letter acronym represents a metagenome-assembled genome (MAG, see Supplementary Data 4 for list of acronyms). The thickness of the lines is scaled with the relative abundance of the connected MAG in the corresponding consortium. A line is connected between a MAG and a metabolite if the pathway responsible for the utilization/production of the metabolite is at least 75% complete in the MAG. The dominant member (> 1% relative abundance) in the metagenome is highlighted with red boxes and the MAG is considered rare. In this consortium the additional metabolic potential provided by the rare bacterial MAG ("Baci") is propionate production.

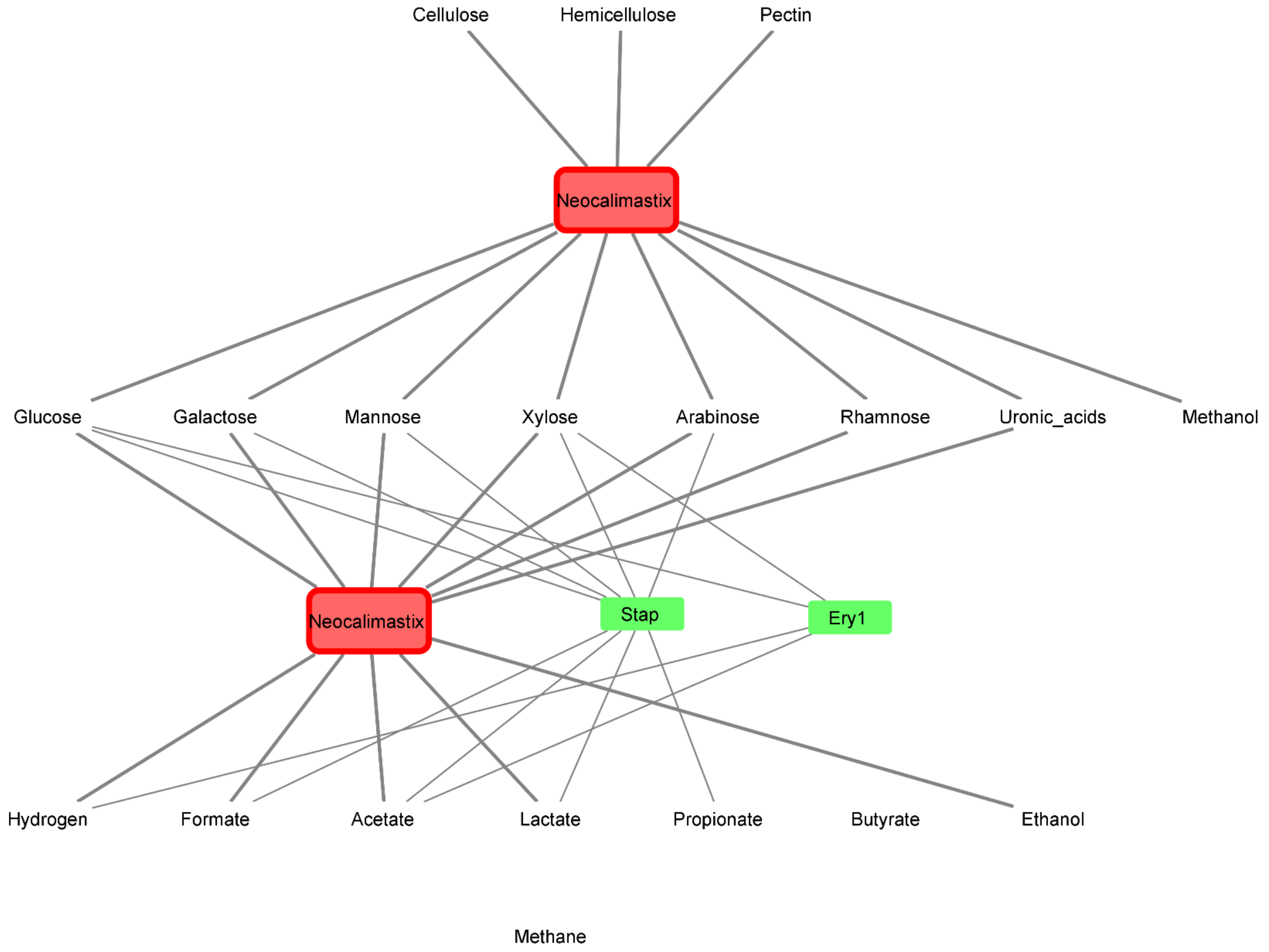

Supplementary Figure 5k. Carbon cross-feeding between microorganisms in the consortium RG10R2-CM (grown on reed canary grass and treated with chloramphenicol, batch 10, replicate 2). Each rectangular shape containing a three-to-five-letter acronym represents a metagenome-assembled genome (MAG, see Supplementary Data 4 for list of acronyms). The thickness of the lines is scaled with the relative abundance of the connected MAG in the corresponding consortium. A line is connected between a MAG and a metabolite if the pathway responsible for the utilization/production of the metabolite is at least 75% complete in the MAG. The dominant member (> 1% relative abundance) in the metagenome is highlighted with red boxes and the MAGs are considered rare. In this consortium the additional metabolic potential provided by a rare bacterial MAG ("Stap") is propionate production.

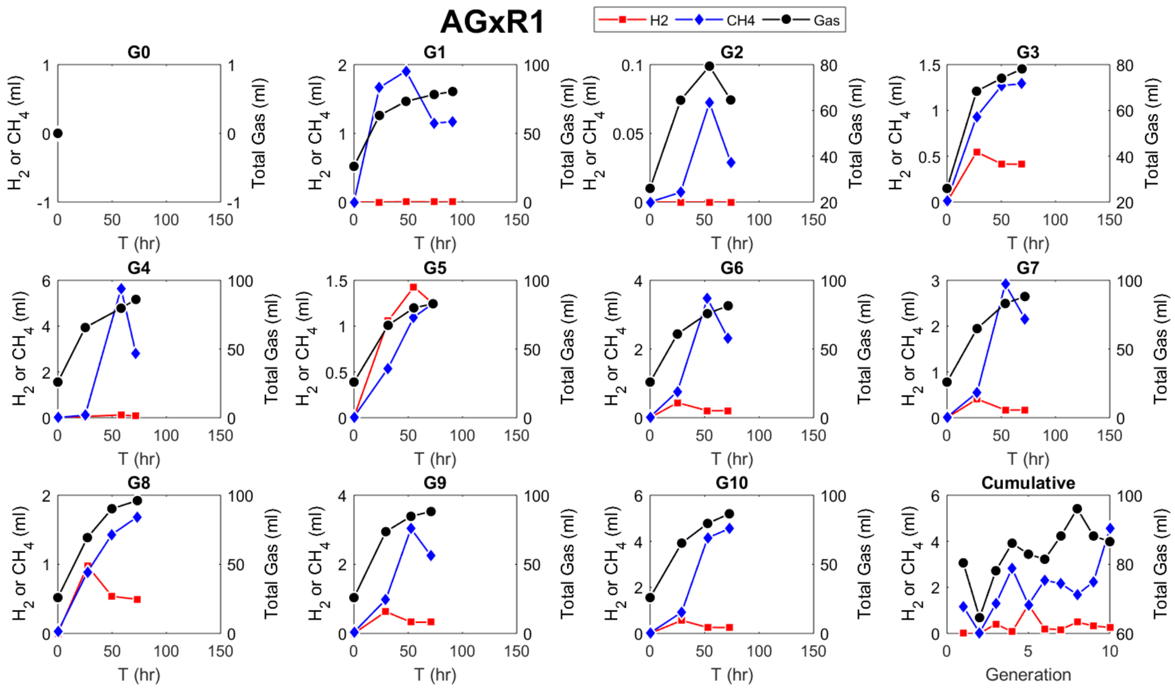

Supplementary Figure 6a. The cumulative production of total gas, hydrogen (H_2), and methane (CH_4) in the first biological replicate of the antibiotics-free consortia grown on alfalfa (AGxR1) over the course of enrichment cultivation in hours (hr). The left y-axis is a scale for the volume of H_2 and CH_4 in milliliters and the right y-axis is a scale for the volume of total gas in milliliters. No measurements were taken for G0. The bottom right sub-Supplementary Figure shows the cumulative production of total gas, H_2 and CH_4 at the end of cultivation of each batch. Red squares represent H_2, blue diamonds represent CH_4, and black circles represent total gas. The cumulative volume of H_2, CH_4, and total gas in ml was calculated with headspace pressure and gas chromatograph concentration measurements as detailed in **Methods**. The total volume of the culturing vessel was 73 ml and the initial volume of the enrichment culture was 50 ml.

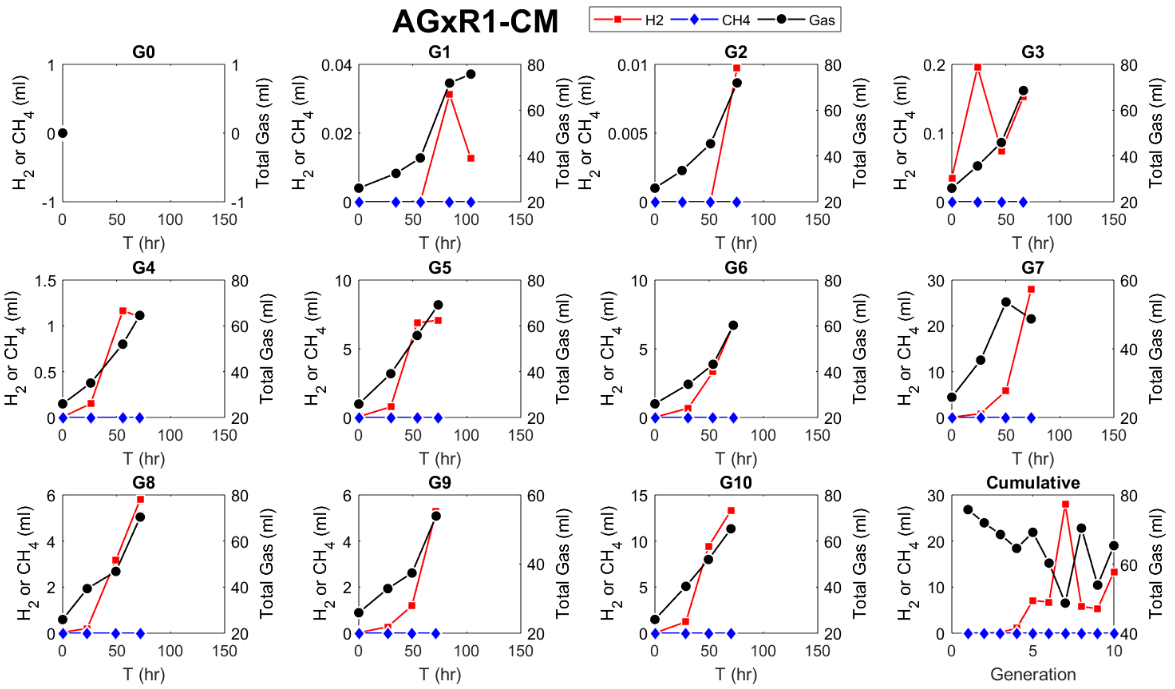

Supplementary Figure 6b. The cumulative production of total gas, hydrogen (H_2), and methane (CH_4) in the first biological replicate of the chloramphenicol-treated consortia grown on alfalfa (AGxR1-CM) over the course of enrichment cultivation in hours (hr). The left y-axis is a scale for the volume of H_2 and CH_4 in milliliters and the right y-axis is a scale for the volume of total gas in milliliters. No measurements were taken for G0. The bottom right sub-Supplementary Figure shows the cumulative production of total gas, H_2 and CH_4 at the end of cultivation of each batch. Red squares represent H_2, blue diamonds represent CH_4, and black circles represent total gas. The cumulative volume of H_2, CH_4, and total gas in ml was calculated with headspace pressure and gas chromatograph concentration measurements as detailed in **Methods**. The total volume of the culturing vessel was 73 ml and the initial volume of the enrichment culture was 50 ml.

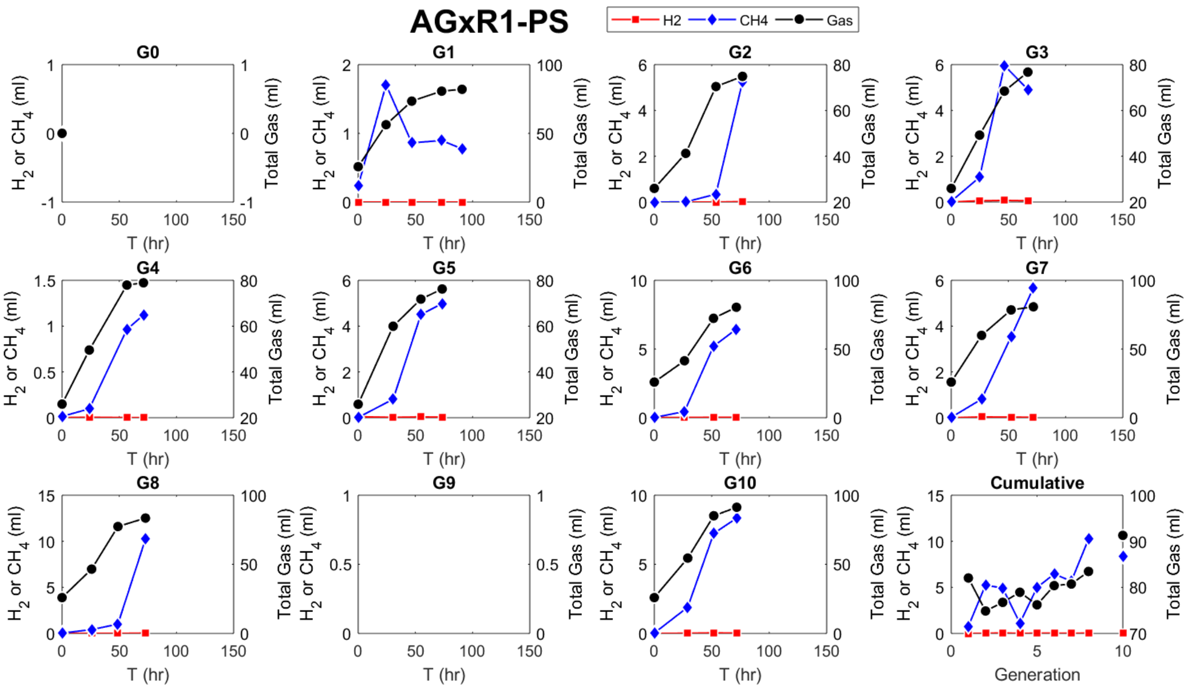

Supplementary Figure 6c. The cumulative production of total gas, hydrogen (H_2), and methane (CH_4) in the first biological replicate of the penicillin and streptomycin-treated consortia grown on alfalfa (AGxR1-PS) over the course of enrichment cultivation in hours (hr). The left y-axis is a scale for the volume of H_2 and CH_4 in milliliters and the right y-axis is a scale for the volume of total gas in milliliters. No measurements were taken for G0. The bottom right sub-Supplementary Figure shows the cumulative production of total gas, H_2 and CH_4 at the end of cultivation of each batch. Red squares represent H_2, blue diamonds represent CH_4, and black circles represent total gas. There was no record for G9 because that sample bottle was accidentally broken during the experiment. The cumulative volume of H_2, CH_4, and total gas in ml was calculated with headspace pressure and gas chromatograph concentration measurements as detailed in **Methods**. The total volume of the culturing vessel was 73 ml and the initial volume of the enrichment culture was 50 ml.

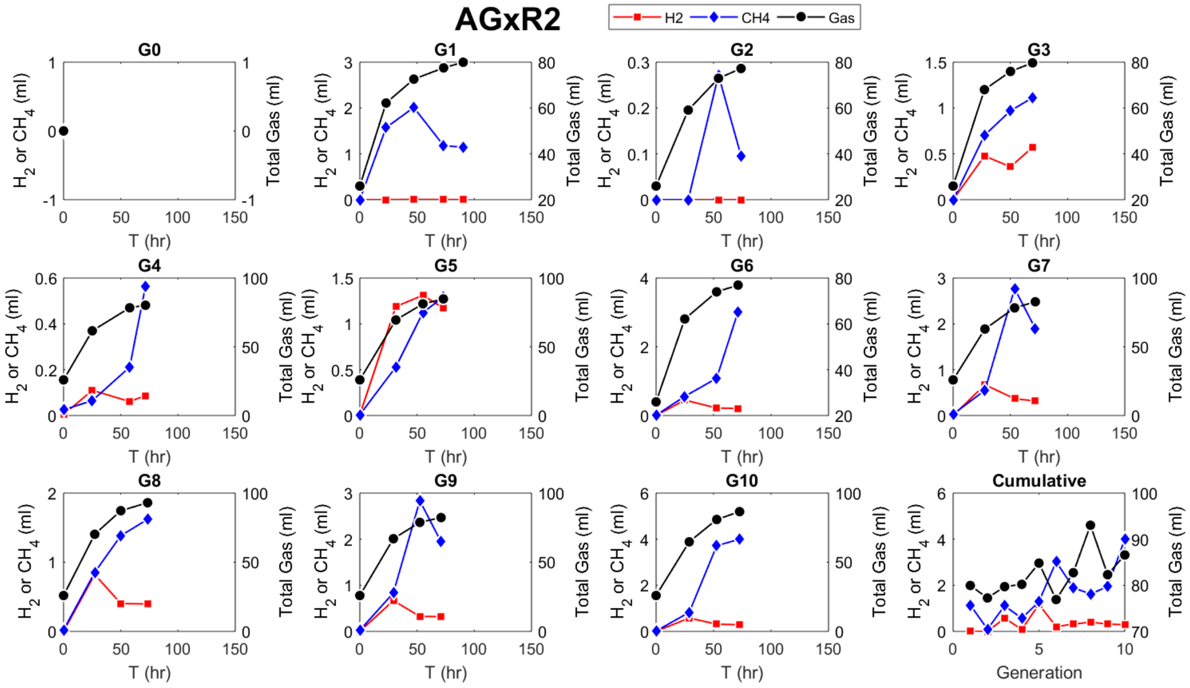

Supplementary Figure 6d. The cumulative production of total gas, hydrogen (H_2), and methane (CH_4) in the second biological replicate of the antibiotics-free consortia grown on alfalfa (AGxR2) over the course of enrichment cultivation in hours (hr). The left y-axis is a scale for the volume of H_2 and CH_4 in milliliters and the right y-axis is a scale for the volume of total gas in milliliters. No measurements were taken for G0. The bottom right sub-Supplementary Figure shows the cumulative production of total gas, H_2 and CH_4 at the end of cultivation of each batch. Red squares represent H_2, blue diamonds represent CH_4, and black circles represent total gas. The cumulative volume of H_2, CH_4, and total gas in ml was calculated with headspace pressure and gas chromatograph concentration measurements as detailed in **Methods**. The total volume of the culturing vessel was 73 ml and the initial volume of the enrichment culture was 50 ml.

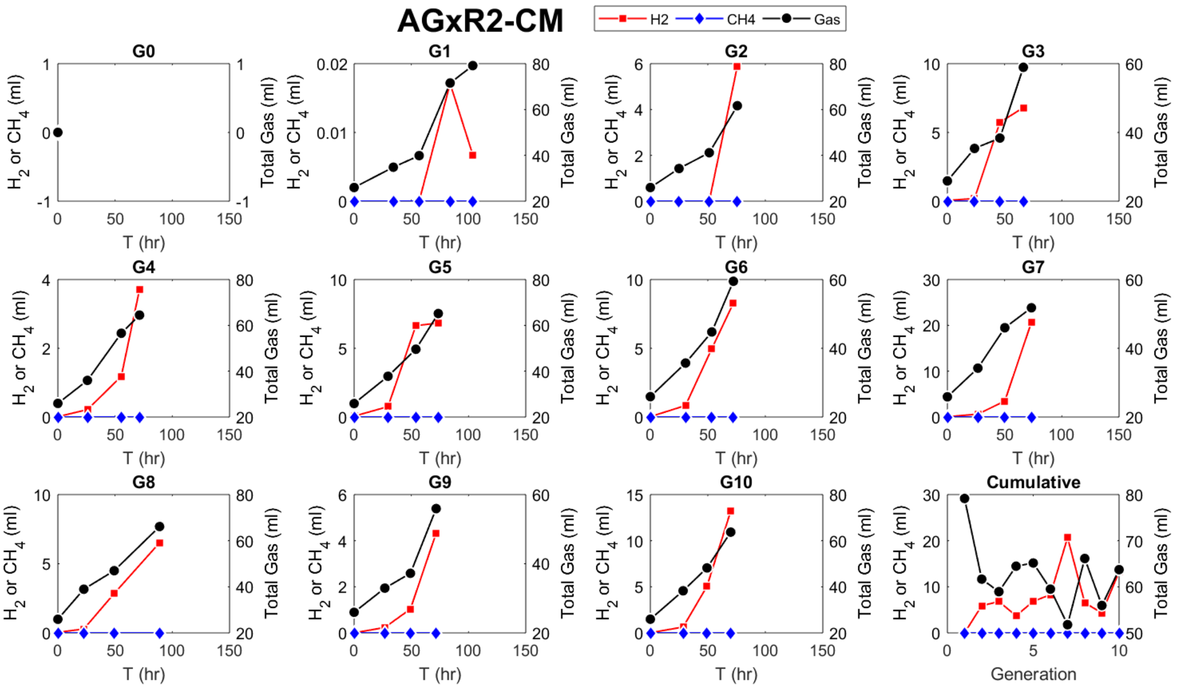

Supplementary Figure 6e. The cumulative production of total gas, hydrogen (H_2), and methane (CH_4) in the second biological replicate of the chloramphenicol-treated consortia grown on alfalfa (AGxR2-CM) over the course of enrichment cultivation in hours (hr). The left y-axis is a scale for the volume of H_2 and CH_4 in milliliters and the right y-axis is a scale for the volume of total gas in milliliters. No measurements were taken for G0. The bottom right sub-Supplementary Figure shows the cumulative production of total gas, H_2 and CH_4 at the end of cultivation of each batch. Red squares represent H_2, blue diamonds represent CH_4, and black circles represent total gas. The cumulative volume of H_2, CH_4, and total gas in ml was calculated with headspace pressure and gas chromatograph concentration measurements as detailed in **Methods**. The total volume of the culturing vessel was 73 ml and the initial volume of the enrichment culture was 50 ml.

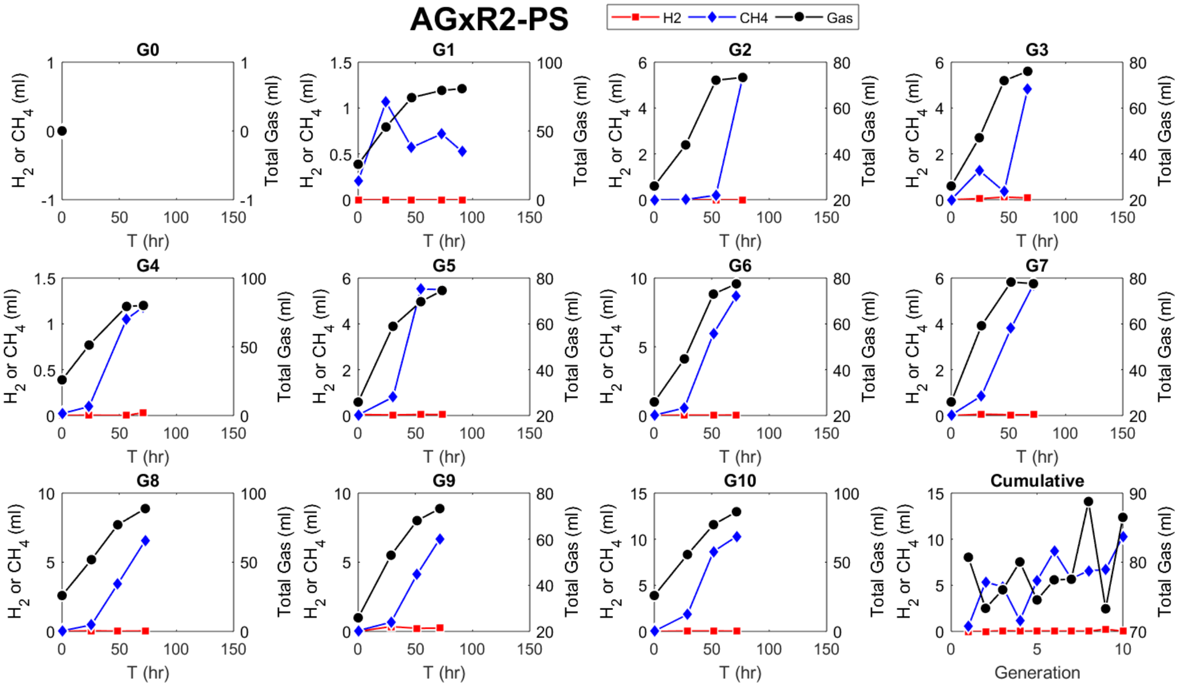

Supplementary Figure 6f. The cumulative production of total gas, hydrogen (H_2), and methane (CH_4) in the second biological replicate of the penicillin and streptomycin-treated consortia grown on alfalfa (AGxR2-PS) over the course of enrichment cultivation in hours (hr). The left y-axis is a scale for the volume of H_2 and CH_4 in milliliters and the right y-axis is a scale for the volume of total gas in milliliters. No measurements were taken for G0. The bottom right sub-Supplementary Figure shows the cumulative production of total gas, H_2 and CH_4 at the end of cultivation of each batch. Red squares represent H_2, blue diamonds represent CH_4, and black circles represent total gas. The cumulative volume of H_2, CH_4, and total gas in ml was calculated with headspace pressure and gas chromatograph concentration measurements as detailed in **Methods**. The total volume of the culturing vessel was 73 ml and the initial volume of the enrichment culture was 50 ml.

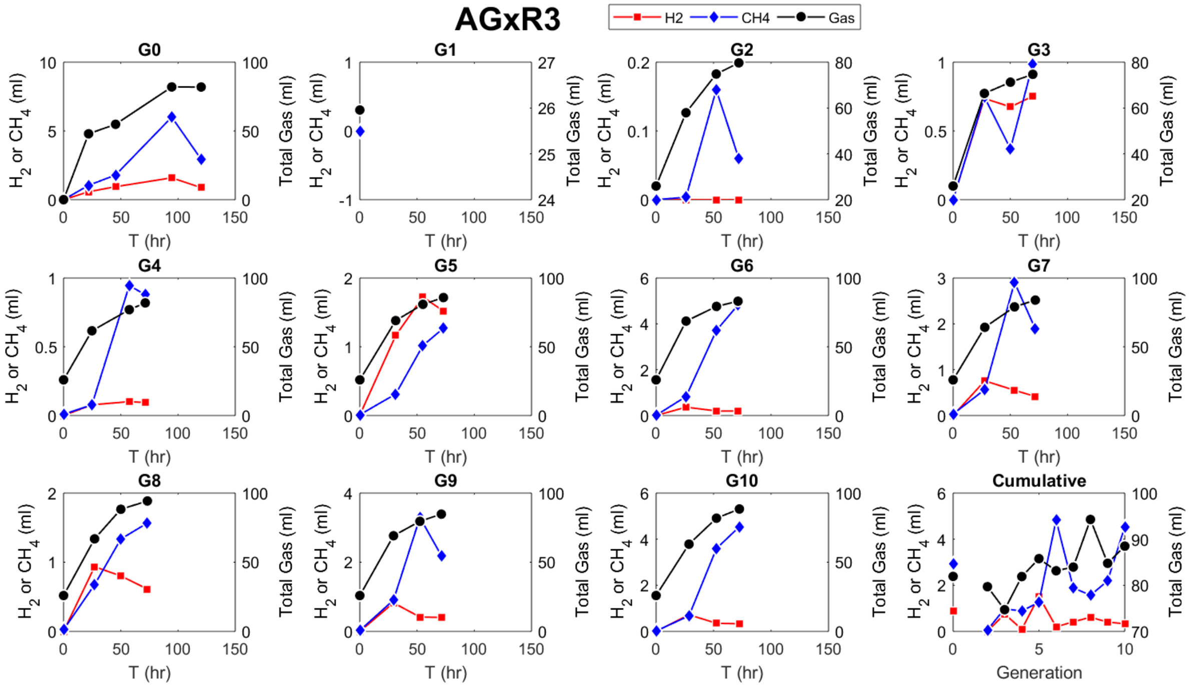

Supplementary Figure 6g. The cumulative production of total gas, hydrogen (H_2), and methane (CH_4) in the third biological replicate of the antibiotics-free consortia grown on alfalfa (AGxR3) over the course of enrichment cultivation in hours (hr). The left y-axis is a scale for the volume of H_2 and CH_4 in milliliters and the right y-axis is a scale for the volume of total gas in milliliters. The bottom right sub-Supplementary Figure shows the cumulative production of total gas, H_2 and CH_4 at the end of cultivation of each batch. Red squares represent H_2, blue diamonds represent CH_4, and black circles represent total gas. There was no record for G1 because that sample bottle was accidentally broken during the experiment. The cumulative volume of H_2, CH_4, and total gas in ml was calculated with headspace pressure and gas chromatograph concentration measurements as detailed in **Methods**. The total volume of the culturing vessel was 73 ml and the initial volume of the enrichment culture was 50 ml.

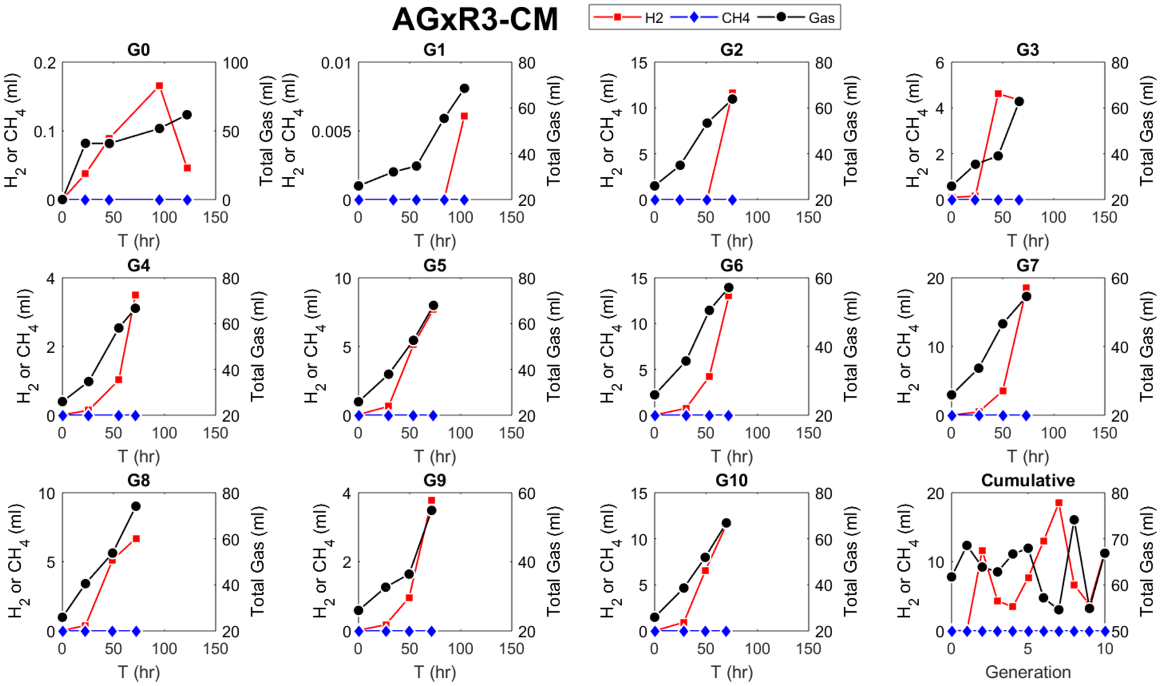

Supplementary Figure 6h. The cumulative production of total gas, hydrogen (H_2), and methane (CH_4) in the third biological replicate of the chloramphenicol-treated consortia grown on alfalfa (AGxR3-CM) over the course of enrichment cultivation in hours (hr). The left y-axis is a scale for the volume of H_2 and CH_4 in milliliters and the right y-axis is a scale for the volume of total gas in milliliters. The bottom right sub-Supplementary Figure shows the cumulative production of total gas, H_2 and CH_4 at the end of cultivation of each batch. Red squares represent H_2, blue diamonds represent CH_4, and black circles represent total gas. The cumulative volume of H_2, CH_4, and total gas in ml was calculated with headspace pressure and gas chromatograph concentration measurements as detailed in **Methods**. The total volume of the culturing vessel was 73 ml and the initial volume of the enrichment culture was 50 ml.

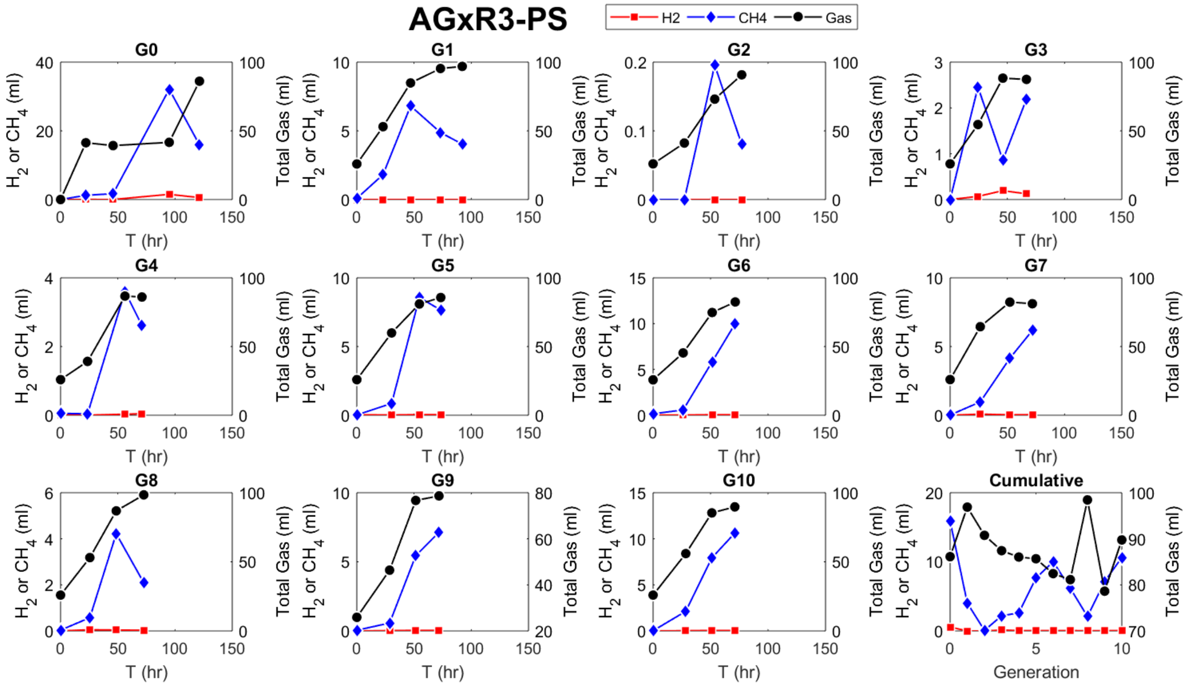

Supplementary Figure 6i. The cumulative production of total gas, hydrogen (H_2), and methane (CH_4) in the third biological replicate of the penicillin and streptomycin-treated consortia grown on alfalfa (AGxR3-PS) over the course of enrichment cultivation in hours (hr). The left y-axis is a scale for the volume of H_2 and CH_4 in milliliters and the right y-axis is a scale for the volume of total gas in milliliters. The bottom right sub-Supplementary Figure shows the cumulative production of total gas, H_2 and CH_4 at the end of cultivation of each batch. Red squares represent H_2, blue diamonds represent CH_4, and black circles represent total gas. The cumulative volume of H_2, CH_4, and total gas in ml was calculated with headspace pressure and gas chromatograph concentration measurements as detailed in **Methods**. The total volume of the culturing vessel was 73 ml and the initial volume of the enrichment culture was 50 ml.

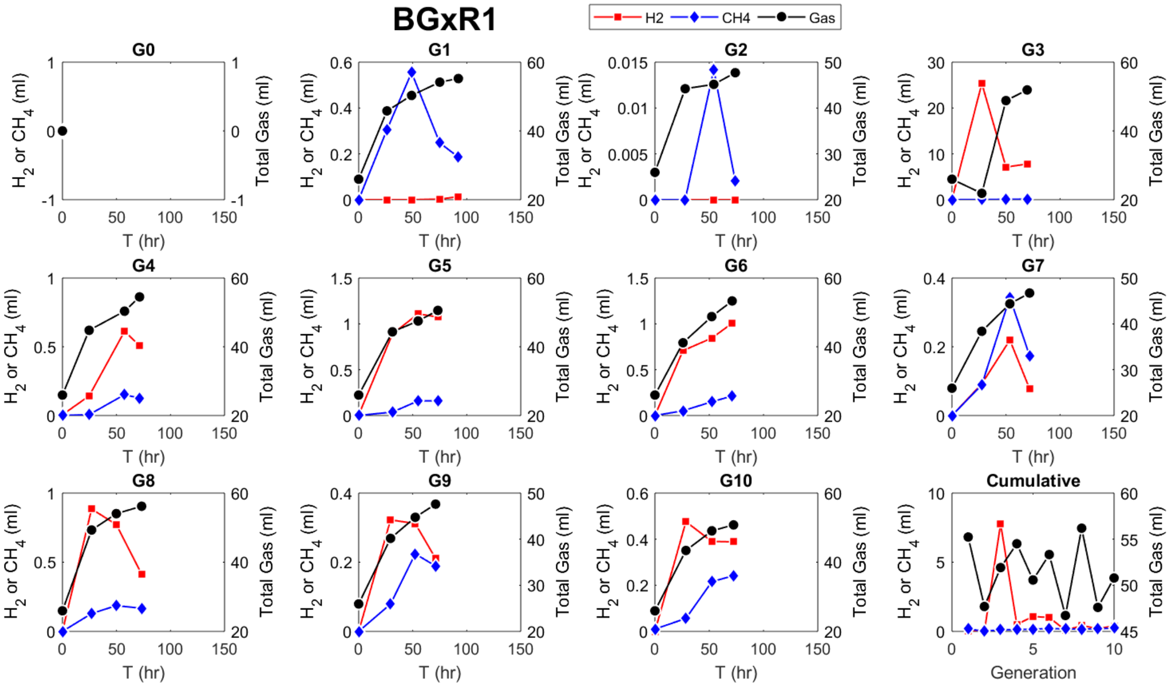

Supplementary Figure 6j. The cumulative production of total gas, hydrogen (H_2), and methane (CH_4) in the first biological replicate of the antibiotics-free consortia grown on bagasse (BGxR1) over the course of enrichment cultivation in hours (hr). The left y-axis is a scale for the volume of H_2 and CH_4 in milliliters and the right y-axis is a scale for the volume of total gas in milliliters. No measurements were taken for G0. The bottom right sub-Supplementary Figure shows the cumulative production of total gas, H_2 and CH_4 at the end of cultivation of each batch. Red squares represent H_2, blue diamonds represent CH_4, and black circles represent total gas. The cumulative volume of H_2, CH_4, and total gas in ml was calculated with headspace pressure and gas chromatograph concentration measurements as detailed in **Methods**. The total volume of the culturing vessel was 73 ml and the initial volume of the enrichment culture was 50 ml.

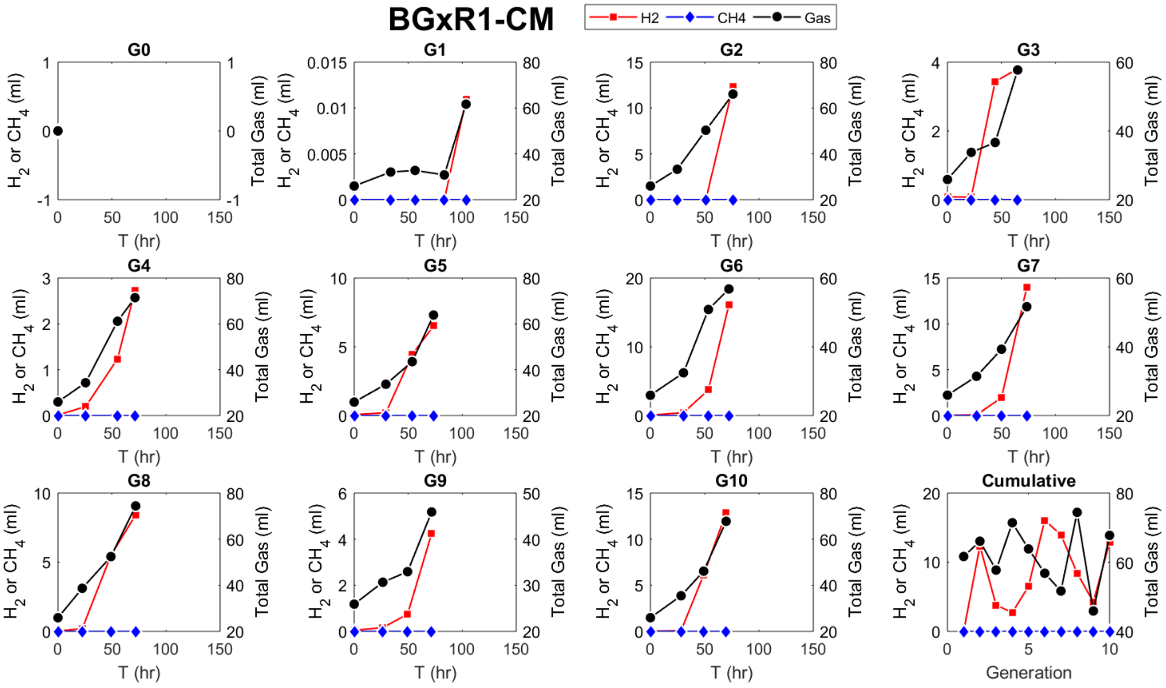

Supplementary Figure 6k. The cumulative production of total gas, hydrogen (H_2), and methane (CH_4) in the first biological replicate of the chloramphenicol-treated consortia grown on bagasse (BGxR1-CM) over the course of enrichment cultivation in hours (hr). The left y-axis is a scale for the volume of H_2 and CH_4 in milliliters and the right y-axis is a scale for the volume of total gas in milliliters. No measurements were taken for G0. The bottom right sub-Supplementary Figure shows the cumulative production of total gas, H_2 and CH_4 at the end of cultivation of each batch. Red squares represent H_2, blue diamonds represent CH_4, and black circles represent total gas. The cumulative volume of H_2, CH_4, and total gas in ml was calculated with headspace pressure and gas chromatograph concentration measurements as detailed in **Methods**. The total volume of the culturing vessel was 73 ml and the initial volume of the enrichment culture was 50 ml.

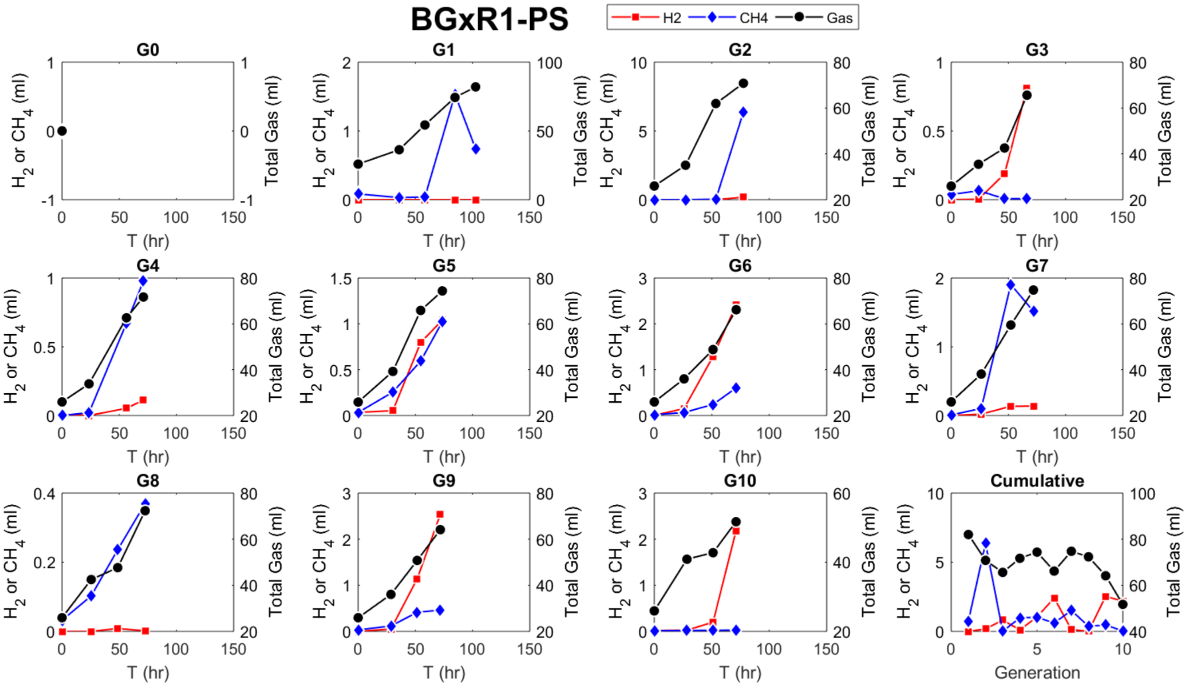

Supplementary Figure 6l. The cumulative production of total gas, hydrogen (H_2), and methane (CH_4) in the first biological replicate of the penicillin and streptomycin-treated consortia grown on bagasse (BGxR1-PS) over the course of enrichment cultivation in hours (hr). The left y-axis is a scale for the volume of H_2 and CH_4 in milliliters and the right y-axis is a scale for the volume of total gas in milliliters. No measurements were taken for G0. The bottom right sub-Supplementary Figure shows the cumulative production of total gas, H_2 and CH_4 at the end of cultivation of each batch. Red squares represent H_2, blue diamonds represent CH_4, and black circles represent total gas. The cumulative volume of H_2, CH_4, and total gas in ml was calculated with headspace pressure and gas chromatograph concentration measurements as detailed in **Methods**. The total volume of the culturing vessel was 73 ml and the initial volume of the enrichment culture was 50 ml.

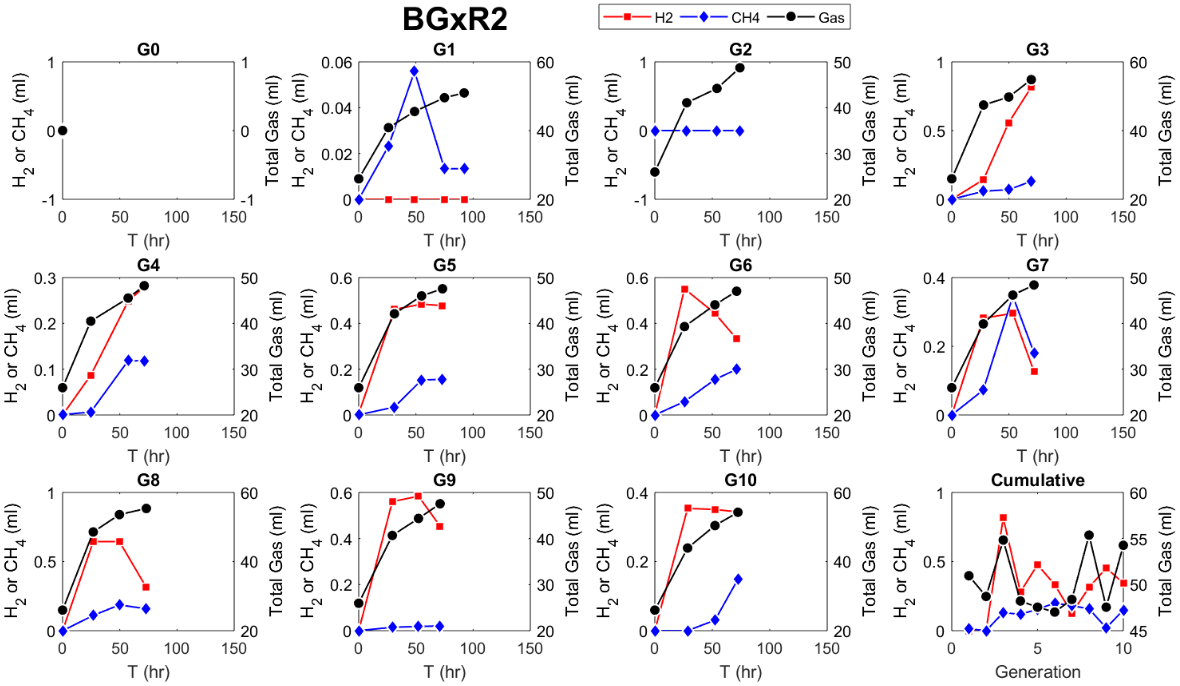

Supplementary Figure 6m. The cumulative production of total gas, hydrogen (H_2), and methane (CH_4) in the second biological replicate of the antibiotics-free consortia grown on bagasse (BGxR2) over the course of enrichment cultivation in hours (hr). The left y-axis is a scale for the volume of H_2 and CH_4 in milliliters and the right y-axis is a scale for the volume of total gas in milliliters. No measurements were taken for G0. The bottom right sub-Supplementary Figure shows the cumulative production of total gas, H_2 and CH_4 at the end of cultivation of each batch. Red squares represent H_2, blue diamonds represent CH_4, and black circles represent total gas. The cumulative volume of H_2, CH_4, and total gas in ml was calculated with headspace pressure and gas chromatograph concentration measurements as detailed in **Methods**. The total volume of the culturing vessel was 73 ml and the initial volume of the enrichment culture was 50 ml.

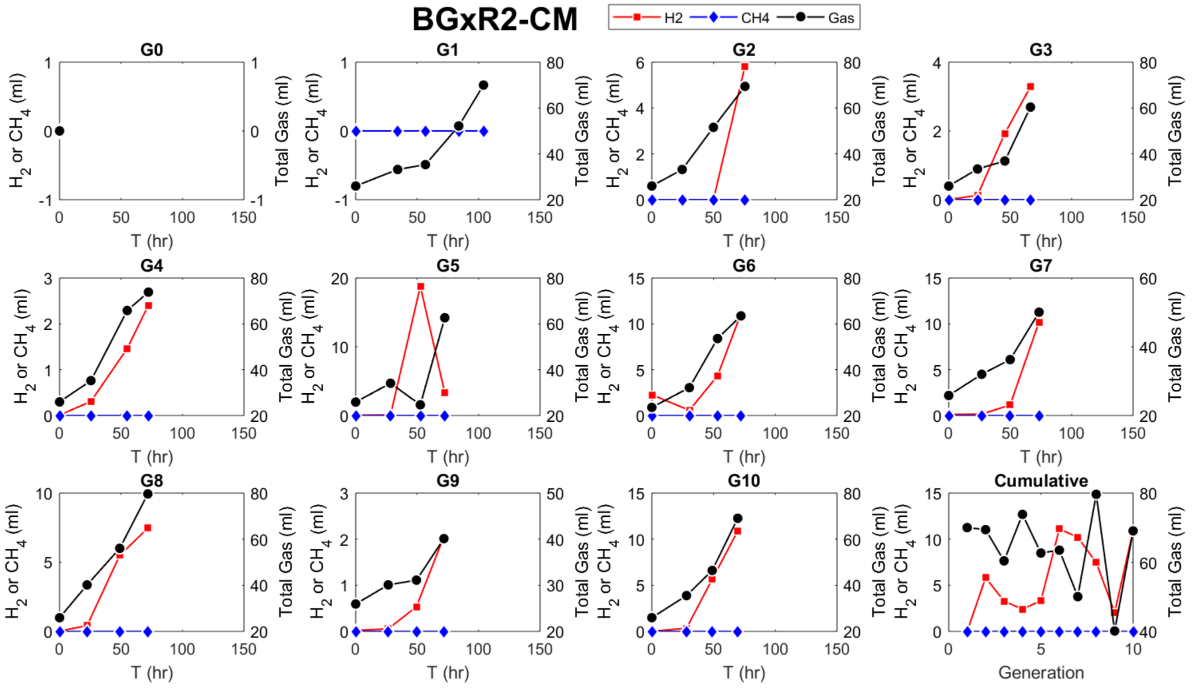

Supplementary Figure 6n. The cumulative production of total gas, hydrogen (H_2), and methane (CH_4) in the second biological replicate of the chloramphenicol-treated consortia grown on bagasse (BGxR2-CM) over the course of enrichment cultivation in hours (hr). The left y-axis is a scale for the volume of H_2 and CH_4 in milliliters and the right y-axis is a scale for the volume of total gas in milliliters. No measurements were taken for G0. The bottom right sub-Supplementary Figure shows the cumulative production of total gas, H_2 and CH_4 at the end of cultivation of each batch. Red squares represent H_2, blue diamonds represent CH_4, and black circles represent total gas. The cumulative volume of H_2, CH_4, and total gas in ml was calculated with headspace pressure and gas chromatograph concentration measurements as detailed in **Methods**. The total volume of the culturing vessel was 73 ml and the initial volume of the enrichment culture was 50 ml.

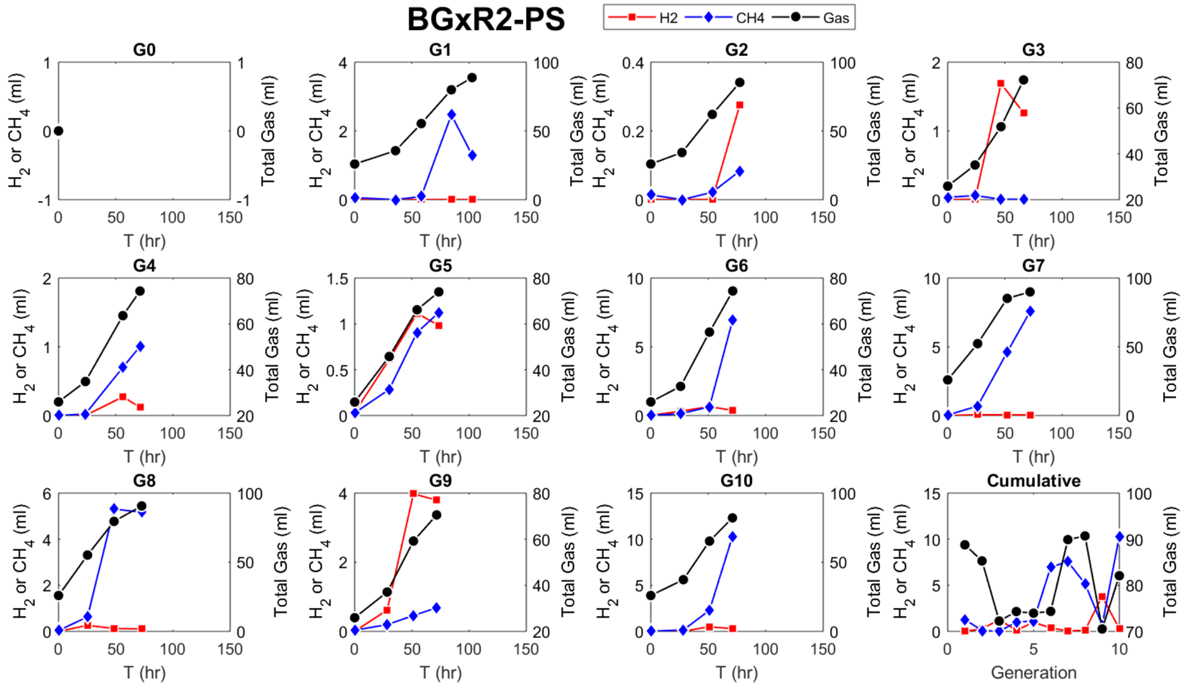

Supplementary Figure 6o. The cumulative production of total gas, hydrogen (H_2), and methane (CH_4) in the second biological replicate of the penicillin and streptomycin-treated consortia grown on bagasse (BGxR2-PS) over the course of enrichment cultivation in hours (hr). The left y-axis is a scale for the volume of H_2 and CH_4 in milliliters and the right y-axis is a scale for the volume of total gas in milliliters. No measurements were taken for G0. The bottom right sub-Supplementary Figure shows the cumulative production of total gas, H_2 and CH_4 at the end of cultivation of each batch. Red squares represent H_2, blue diamonds represent CH_4, and black circles represent total gas. The cumulative volume of H_2, CH_4, and total gas in ml was calculated with headspace pressure and gas chromatograph concentration measurements as detailed in **Methods**. The total volume of the culturing vessel was 73 ml and the initial volume of the enrichment culture was 50 ml.

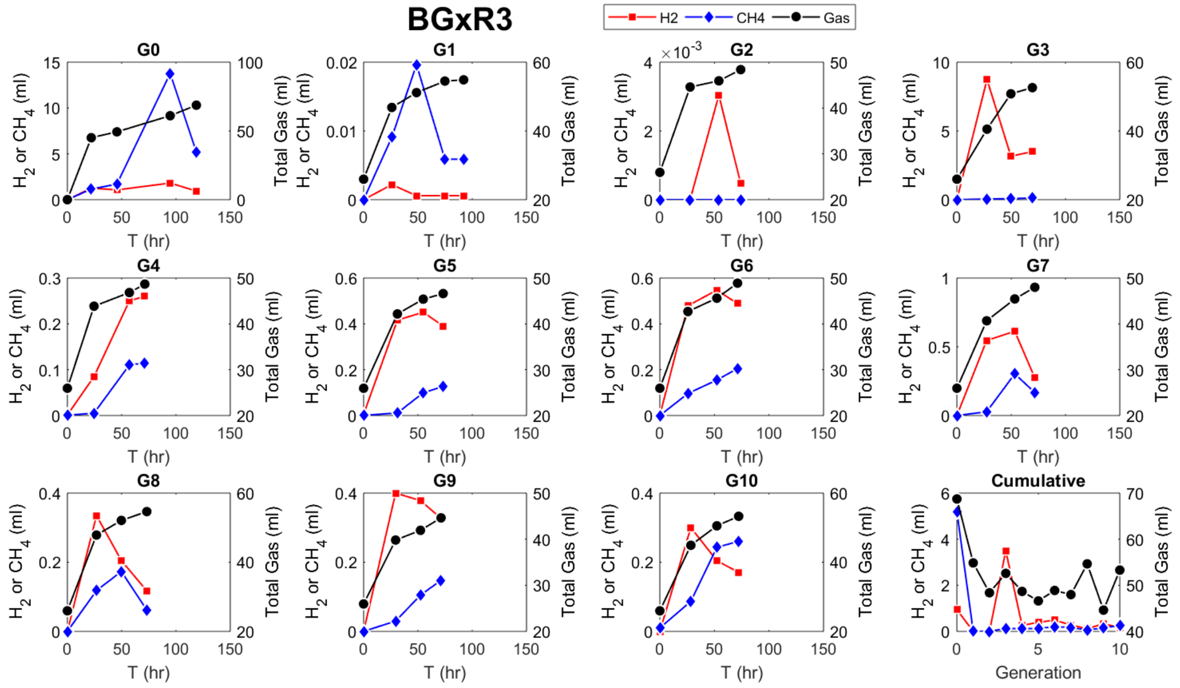

Supplementary Figure 6p. The cumulative production of total gas, hydrogen (H_2), and methane (CH_4) in the third biological replicate of the antibiotics-free consortia grown on bagasse (BGxR3) over the course of enrichment cultivation in hours (hr). The left y-axis is a scale for the volume of H_2 and CH_4 in milliliters and the right y-axis is a scale for the volume of total gas in milliliters. The bottom right sub-Supplementary Figure shows the cumulative production of total gas, H_2 and CH_4 at the end of cultivation of each batch. Red squares represent H_2, blue diamonds represent CH_4, and black circles represent total gas. The cumulative volume of H_2, CH_4, and total gas in ml was calculated with headspace pressure and gas chromatograph concentration measurements as detailed in **Methods**. The total volume of the culturing vessel was 73 ml and the initial volume of the enrichment culture was 50 ml.

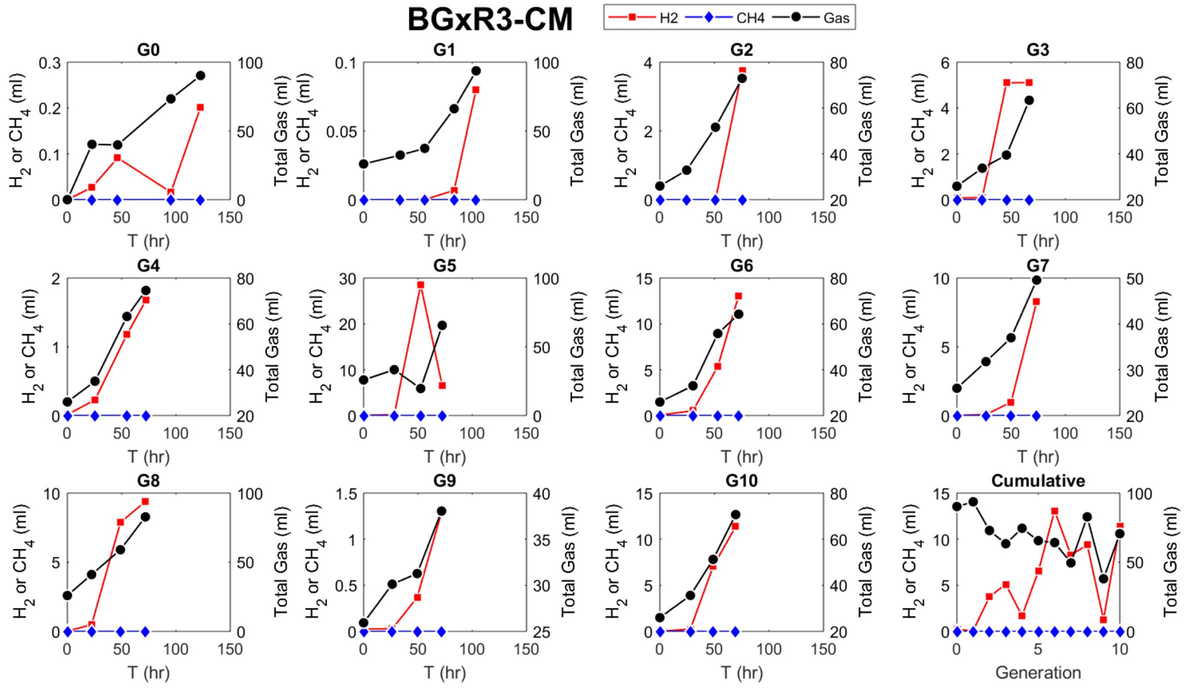

Supplementary Figure 6q. The cumulative production of total gas, hydrogen (H_2), and methane (CH_4) in the third biological replicate of the chloramphenicol-treated consortia grown on bagasse (BGxR3-CM) over the course of enrichment cultivation in hours (hr). The left y-axis is a scale for the volume of H_2 and CH_4 in milliliters and the right y-axis is a scale for the volume of total gas in milliliters. The bottom right sub-Supplementary Figure shows the cumulative production of total gas, H_2 and CH_4 at the end of cultivation of each batch. Red squares represent H_2, blue diamonds represent CH_4, and black circles represent total gas. The cumulative volume of H_2, CH_4, and total gas in ml was calculated with headspace pressure and gas chromatograph concentration measurements as detailed in **Methods**. The total volume of the culturing vessel was 73 ml and the initial volume of the enrichment culture was 50 ml.

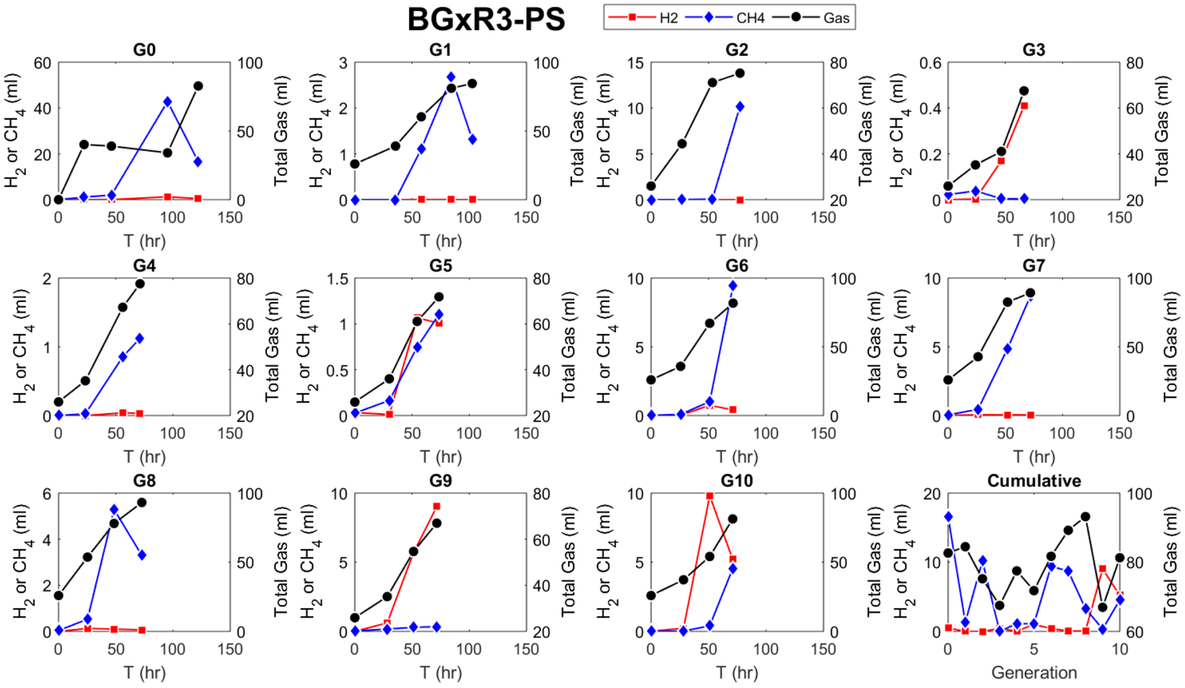

Supplementary Figure 6r. The cumulative production of total gas, hydrogen (H_2), and methane (CH_4) in the third biological replicate of the penicillin and streptomycin-treated consortia grown on bagasse (BGxR3-PS) over the course of enrichment cultivation in hours (hr). The left y-axis is a scale for the volume of H_2 and CH_4 in milliliters and the right y-axis is a scale for the volume of total gas in milliliters. The bottom right sub-Supplementary Figure shows the cumulative production of total gas, H_2 and CH_4 at the end of cultivation of each batch. Red squares represent H_2, blue diamonds represent CH_4, and black circles represent total gas. The cumulative volume of H_2, CH_4, and total gas in ml was calculated with headspace pressure and gas chromatograph concentration measurements as detailed in **Methods**. The total volume of the culturing vessel was 73 ml and the initial volume of the enrichment culture was 50 ml.

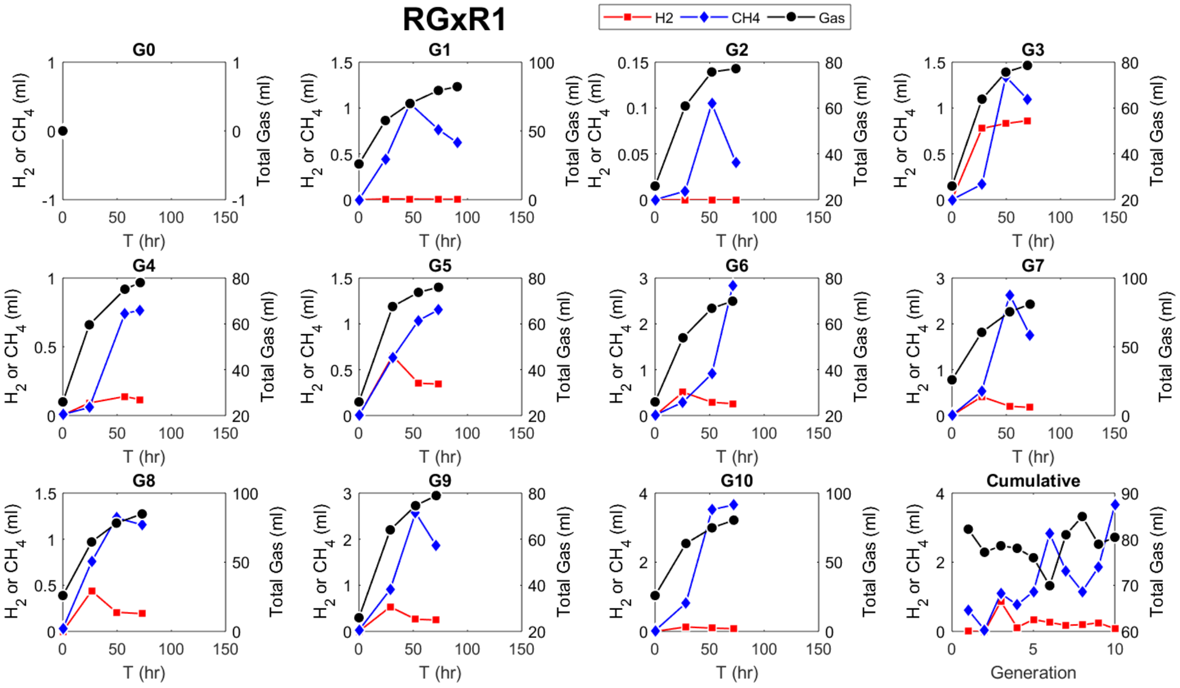

Supplementary Figure 6s. The cumulative production of total gas, hydrogen (H_2), and methane (CH_4) in the first biological replicate of the antibiotics-free consortia grown on reed canary grass (RGxR1) over the course of enrichment cultivation in hours (hr). The left y-axis is a scale for the volume of H_2 and CH_4 in milliliters and the right y-axis is a scale for the volume of total gas in milliliters. No measurements were taken for G0. The bottom right sub-Supplementary Figure shows the cumulative production of total gas, H_2 and CH_4 at the end of cultivation of each batch. Red squares represent H_2, blue diamonds represent CH_4, and black circles represent total gas. The cumulative volume of H_2, CH_4, and total gas in ml was calculated with headspace pressure and gas chromatograph concentration measurements as detailed in **Methods**. The total volume of the culturing vessel was 73 ml and the initial volume of the enrichment culture was 50 ml.

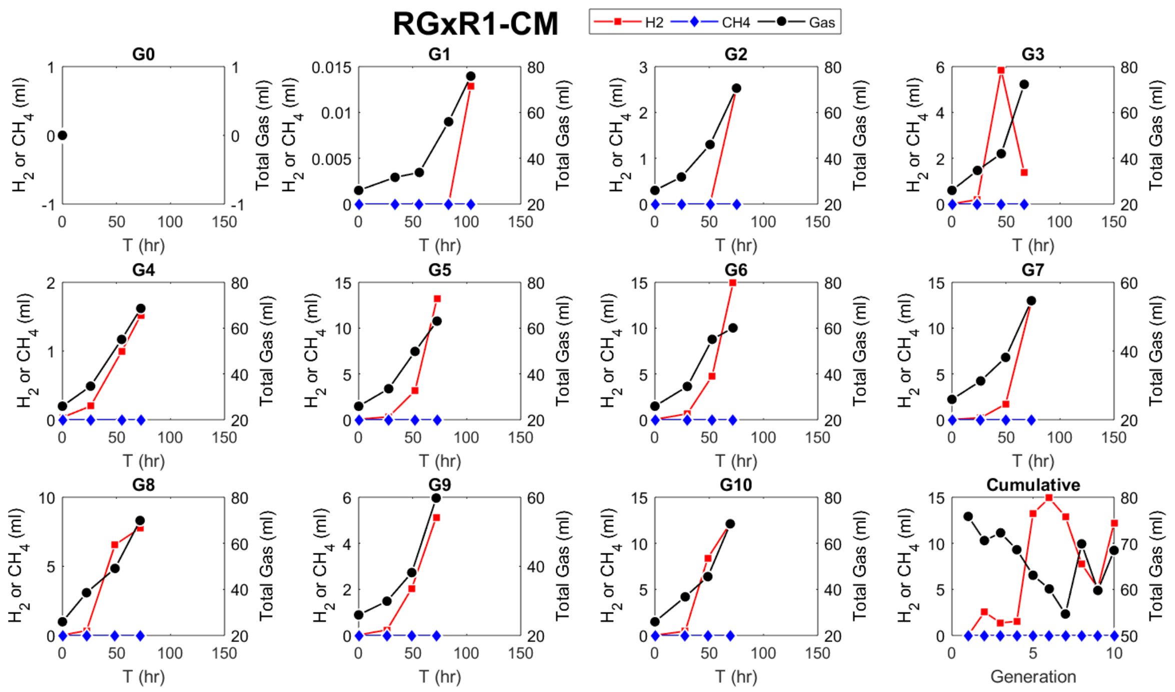

Supplementary Figure 6t. The cumulative production of total gas, hydrogen (H_2), and methane (CH_4) in the first biological replicate of the chloramphenicol-treated consortia grown on reed canary grass (RGxR1-CM) over the course of enrichment cultivation in hours (hr). The left y-axis is a scale for the volume of H_2 and CH_4 in milliliters and the right y-axis is a scale for the volume of total gas in milliliters. No measurements were taken for G0. The bottom right sub-Supplementary Figure shows the cumulative production of total gas, H_2 and CH_4 at the end of cultivation of each batch. Red squares represent H_2, blue diamonds represent CH_4, and black circles represent total gas. The cumulative volume of H_2, CH_4, and total gas in ml was calculated with headspace pressure and gas chromatograph concentration measurements as detailed in **Methods**. The total volume of the culturing vessel was 73 ml and the initial volume of the enrichment culture was 50 ml.

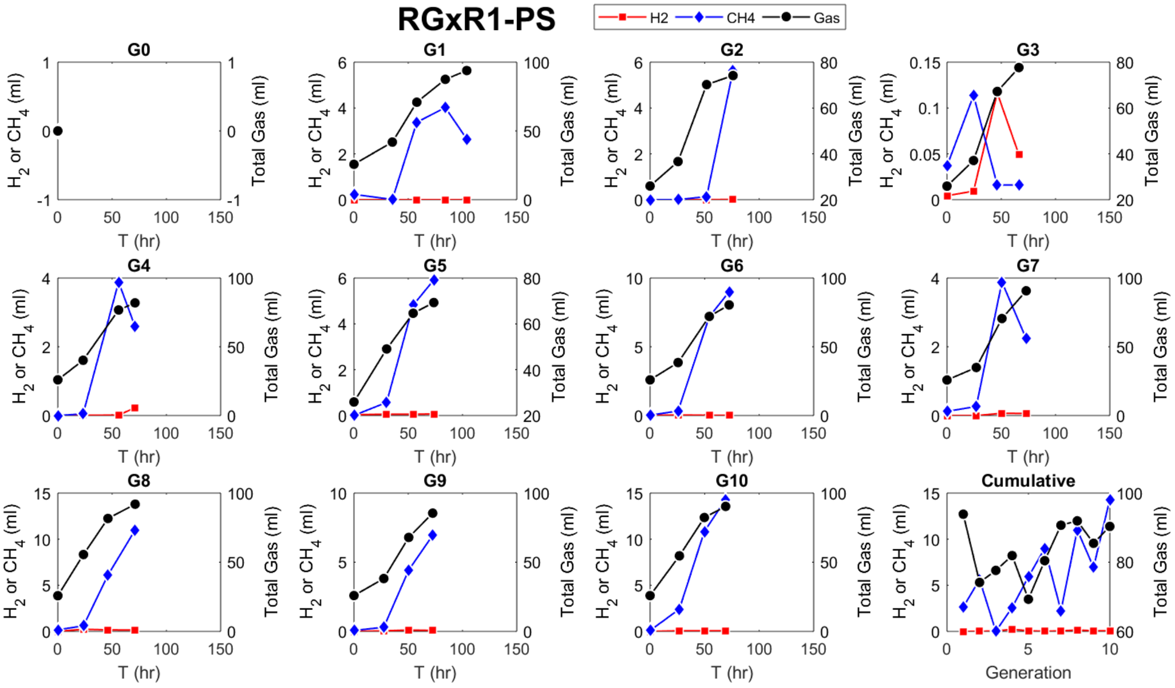

Supplementary Figure 6u. The cumulative production of total gas, hydrogen (H_2), and methane (CH_4) in the first biological replicate of the penicillin and streptomycin-treated consortia grown on reed canary grass (RGxR1-PS) over the course of enrichment cultivation in hours (hr). The left y-axis is a scale for the volume of H_2 and CH_4 in milliliters and the right y-axis is a scale for the volume of total gas in milliliters. No measurements were taken for G0. The bottom right sub-Supplementary Figure shows the cumulative production of total gas, H_2 and CH_4 at the end of cultivation of each batch. Red squares represent H_2, blue diamonds represent CH_4, and black circles represent total gas. The cumulative volume of H_2, CH_4, and total gas in ml was calculated with headspace pressure and gas chromatograph concentration measurements as detailed in **Methods**. The total volume of the culturing vessel was 73 ml and the initial volume of the enrichment culture was 50 ml.

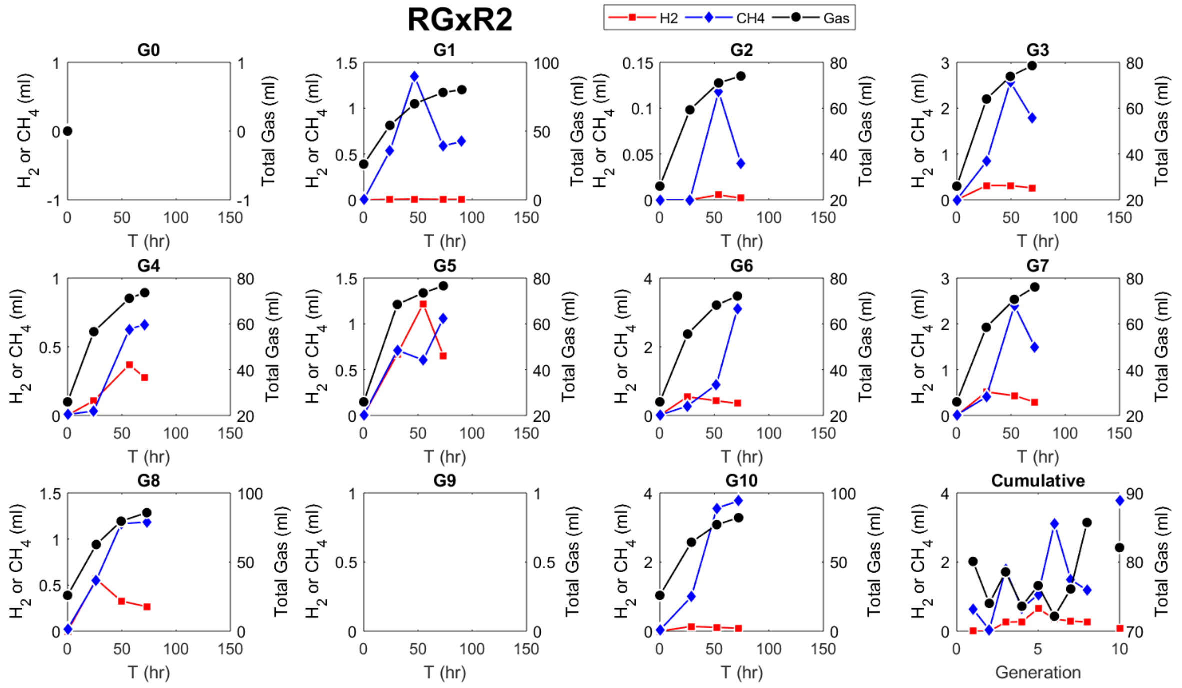

Supplementary Figure 6v. The cumulative production of total gas, hydrogen (H_2), and methane (CH_4) in the second biological replicate of the antibiotics-free consortia grown on reed canary grass (RGxR2) over the course of enrichment cultivation in hours (hr). The left y-axis is a scale for the volume of H_2 and CH_4 in milliliters and the right y-axis is a scale for the volume of total gas in milliliters. No measurements were taken for G0. The bottom right sub-Supplementary Figure shows the cumulative production of total gas, H_2 and CH_4 at the end of cultivation of each batch. Red squares represent H_2, blue diamonds represent CH_4, and black circles represent total gas. There was no record for G9 because that sample bottle was accidentally broken during the experiment. The cumulative volume of H_2, CH_4, and total gas in ml was calculated with headspace pressure and gas chromatograph concentration measurements as detailed in **Methods**. The total volume of the culturing vessel was 73 ml and the initial volume of the enrichment culture was 50 ml.

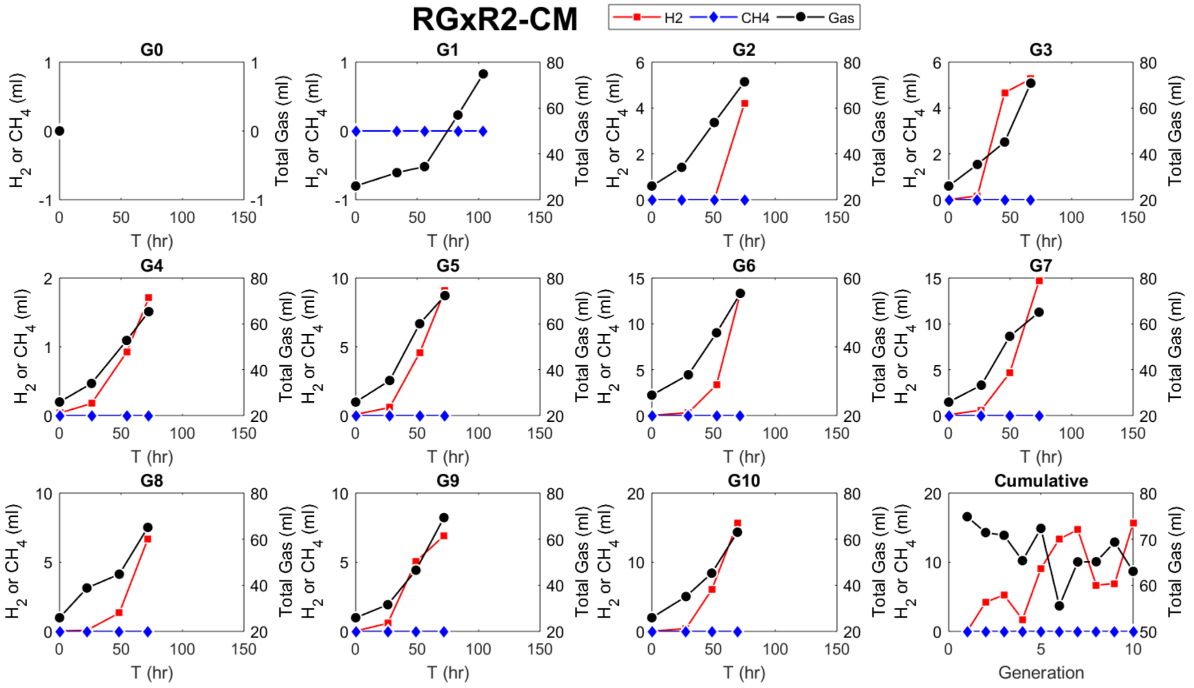

Supplementary Figure 6w. The cumulative production of total gas, hydrogen (H_2), and methane (CH_4) in the second biological replicate of the chloramphenicol-treated consortia grown on reed canary grass (RGxR2-CM) over the course of enrichment cultivation in hours (hr). The left y-axis is a scale for the volume of H_2 and CH_4 in milliliters and the right y-axis is a scale for the volume of total gas in milliliters. No measurements were taken for G0. The bottom right sub-Supplementary Figure shows the cumulative production of total gas, H_2 and CH_4 at the end of cultivation of each batch. Red squares represent H_2, blue diamonds represent CH_4, and black circles represent total gas. The cumulative volume of H_2, CH_4, and total gas in ml was calculated with headspace pressure and gas chromatograph concentration measurements as detailed in **Methods**. The total volume of the culturing vessel was 73 ml and the initial volume of the enrichment culture was 50 ml.

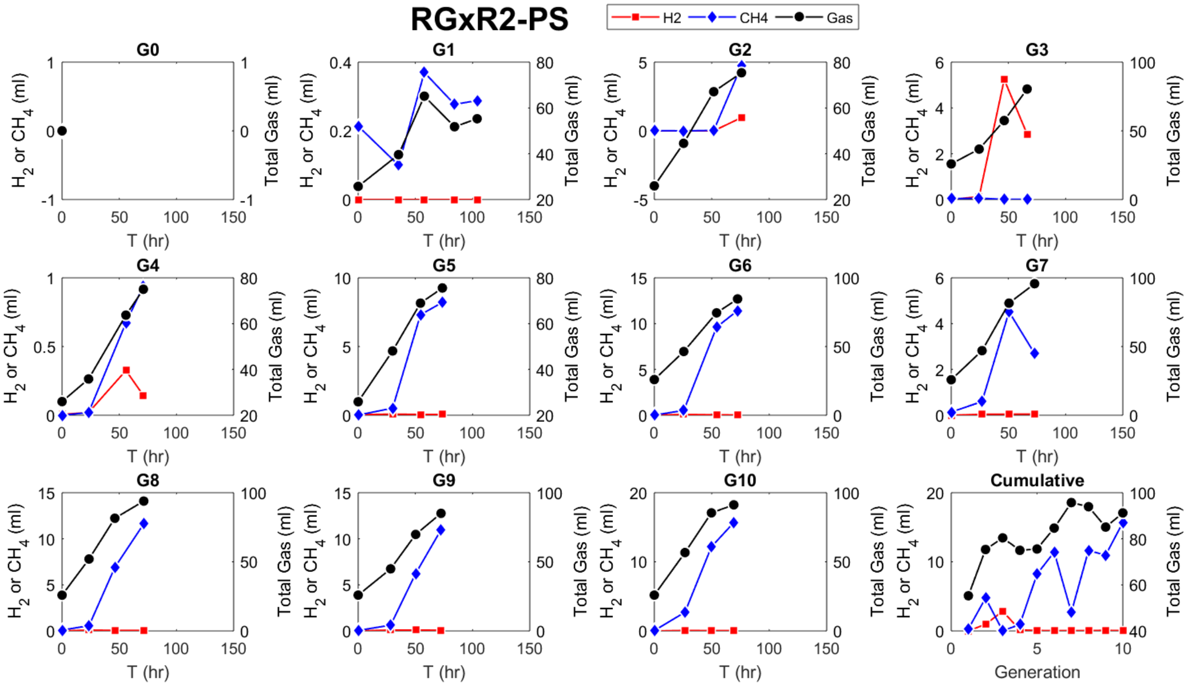

Supplementary Figure 6x. The cumulative production of total gas, hydrogen (H_2), and methane (CH_4) in the second biological replicate of the penicillin and streptomycin-treated consortia grown on reed canary grass (RGxR2-PS) over the course of enrichment cultivation in hours (hr). The left y-axis is a scale for the volume of H_2 and CH_4 in milliliters and the right y-axis is a scale for the volume of total gas in milliliters. No measurements were taken for G0. The bottom right sub-Supplementary Figure shows the cumulative production of total gas, H_2 and CH_4 at the end of cultivation of each batch. Red squares represent H_2, blue diamonds represent CH_4, and black circles represent total gas. The cumulative volume of H_2, CH_4, and total gas in ml was calculated with headspace pressure and gas chromatograph concentration measurements as detailed in **Methods**. The total volume of the culturing vessel was 73 ml and the initial volume of the enrichment culture was 50 ml.

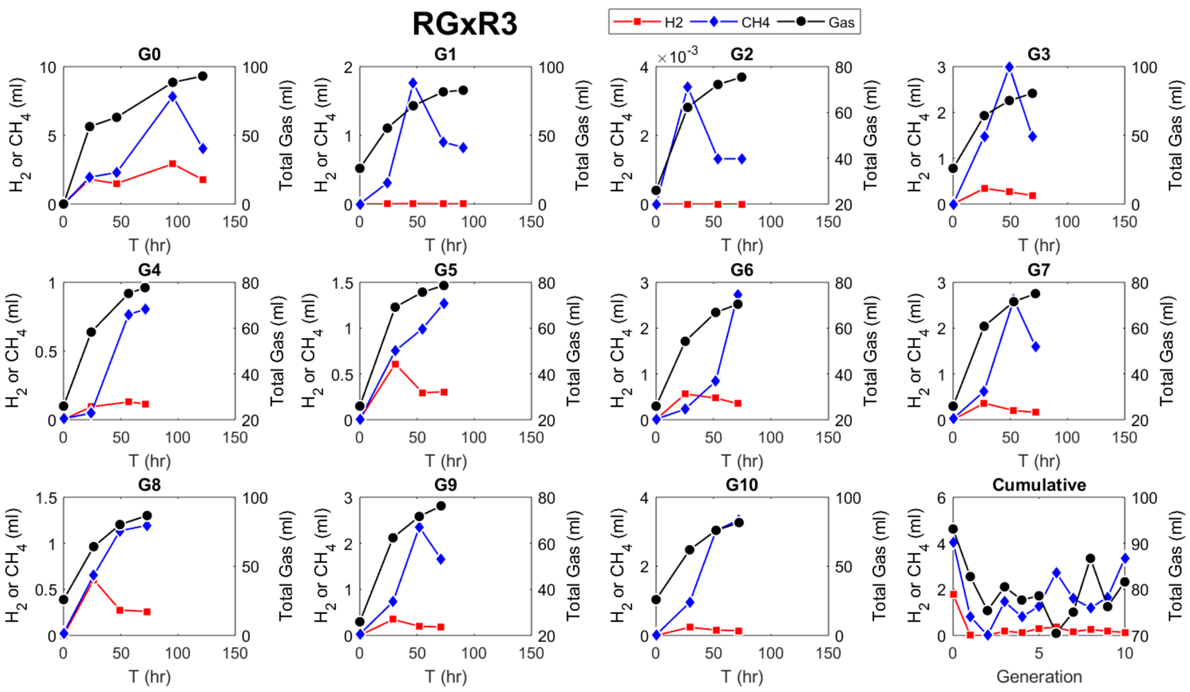

Supplementary Figure 6y. The cumulative production of total gas, hydrogen (H_2), and methane (CH_4) in the third biological replicate of the antibiotics-free consortia grown on reed canary grass (RGxR3) over the course of enrichment cultivation in hours (hr). The left y-axis is a scale for the volume of H_2 and CH_4 in milliliters and the right y-axis is a scale for the volume of total gas in milliliters. The bottom right sub-Supplementary Figure shows the cumulative production of total gas, H_2 and CH_4 at the end of cultivation of each batch. Red squares represent H_2, blue diamonds represent CH_4, and black circles represent total gas. The cumulative volume of H_2, CH_4, and total gas in ml was calculated with headspace pressure and gas chromatograph concentration measurements as detailed in **Methods**. The total volume of the culturing vessel was 73 ml and the initial volume of the enrichment culture was 50 ml.

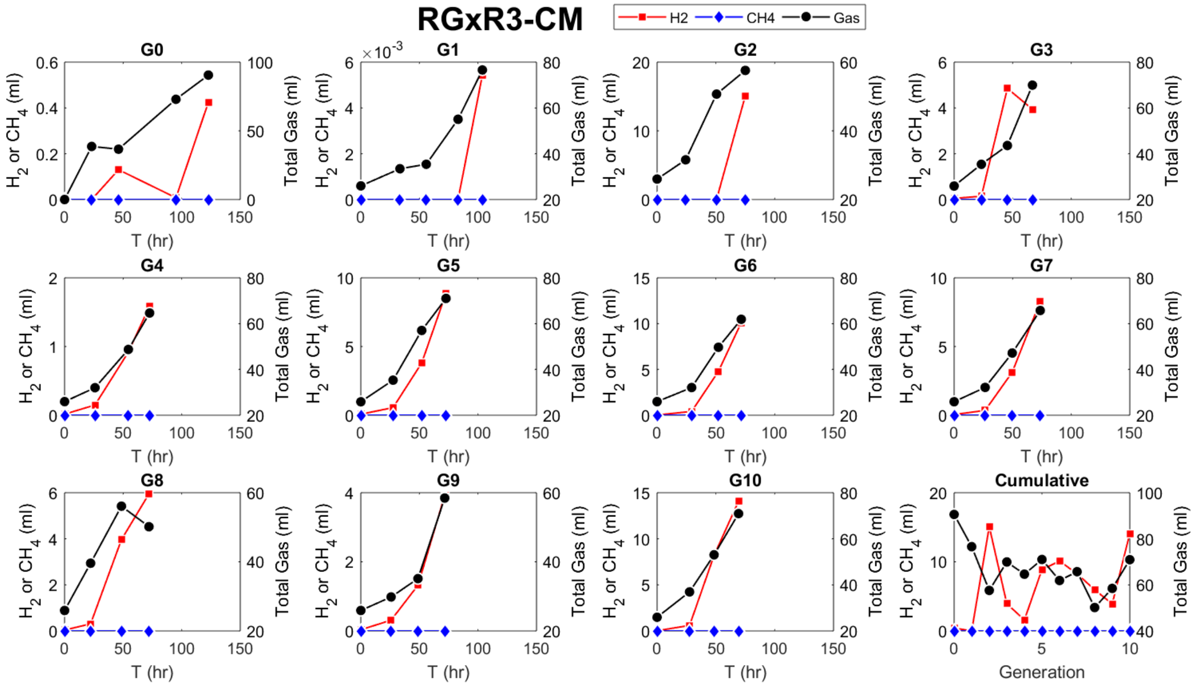

Supplementary Figure 6z. The cumulative production of total gas, hydrogen (H_2), and methane (CH_4) in the third biological replicate of the chloramphenicol-treated consortia grown on reed canary grass (RGxR3-CM) over the course of enrichment cultivation in hours (hr). The left y-axis is a scale for the volume of H_2 and CH_4 in milliliters and the right y-axis is a scale for the volume of total gas in milliliters. The bottom right sub-Supplementary Figure shows the cumulative production of total gas, H_2 and CH_4 at the end of cultivation of each batch. Red squares represent H_2, blue diamonds represent CH_4, and black circles represent total gas. The cumulative volume of H_2, CH_4, and total gas in ml was calculated with headspace pressure and gas chromatograph concentration measurements as detailed in **Methods**. The total volume of the culturing vessel was 73 ml and the initial volume of the enrichment culture was 50 ml.

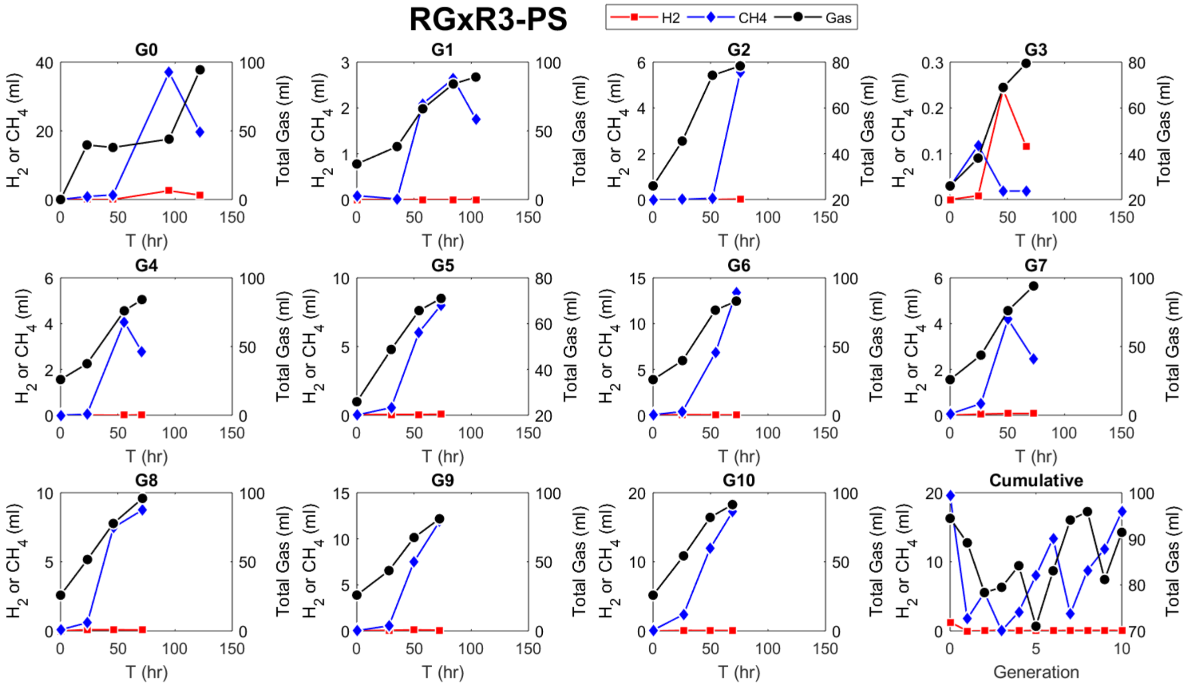

Supplementary Figure 6aa. The cumulative production of total gas, hydrogen (H_2), and methane (CH_4) in the third biological replicate of the penicillin and streptomycin-treated consortia grown on reed canary grass (RGxR3-PS) over the course of enrichment cultivation in hours (hr). The left y-axis is a scale for the volume of H_2 and CH_4 in milliliters and the right y-axis is a scale for the volume of total gas in milliliters. The bottom right sub-Supplementary Figure shows the cumulative production of total gas, H_2 and CH_4 at the end of cultivation of each batch. Red squares represent H_2, blue diamonds represent CH_4, and black circles represent total gas. The cumulative volume of H_2, CH_4, and total gas in ml was calculated with headspace pressure and gas chromatograph concentration measurements as detailed in **Methods**. The total volume of the culturing vessel was 73 ml and the initial volume of the enrichment culture was 50 ml.

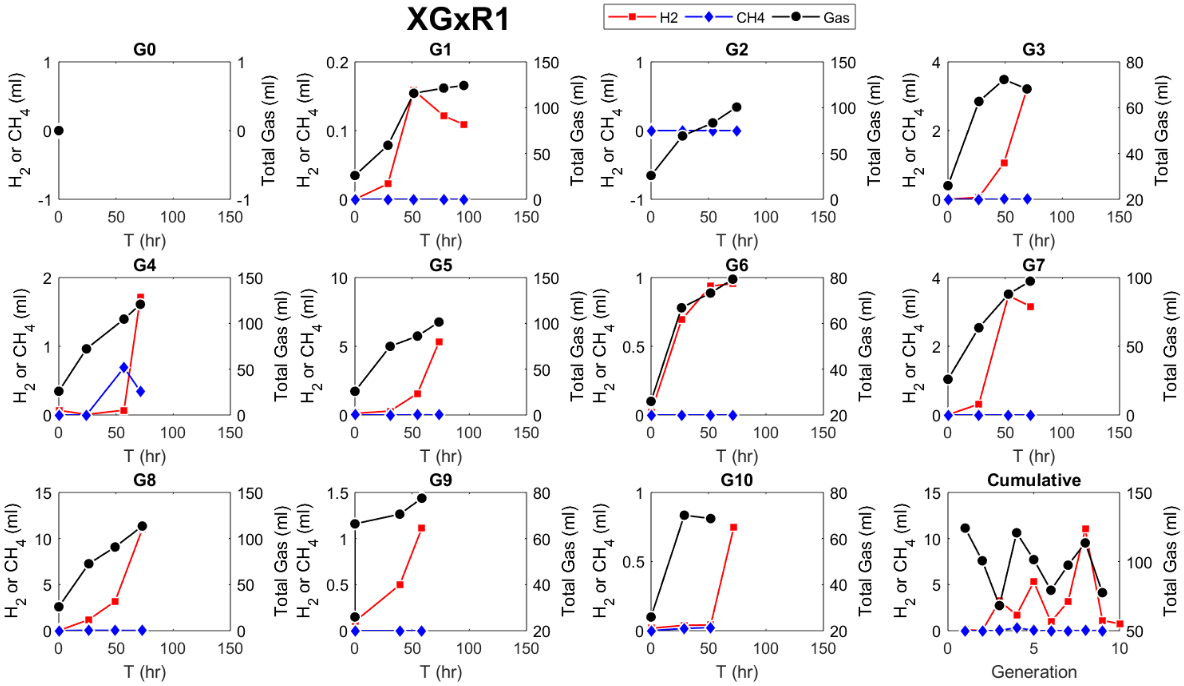

Supplementary Figure 6ab. The cumulative production of total gas, hydrogen (H_2), and methane (CH_4) in the first biological replicate of the antibiotics-free consortia grown on xylan (XGxR1) over the course of enrichment cultivation in hours (hr). The left y-axis is a scale for the volume of H_2 and CH_4 in milliliters and the right y-axis is a scale for the volume of total gas in milliliters. No measurements were taken for G0. The bottom right sub-Supplementary Figure shows the cumulative production of total gas, H_2 and CH_4 at the end of cultivation of each batch. Red squares represent H_2, blue diamonds represent CH_4, and black circles represent total gas. The cumulative volume of H_2, CH_4, and total gas in ml was calculated with headspace pressure and gas chromatograph concentration measurements as detailed in **Methods**. The total volume of the culturing vessel was 73 ml and the initial volume of the enrichment culture was 50 ml.

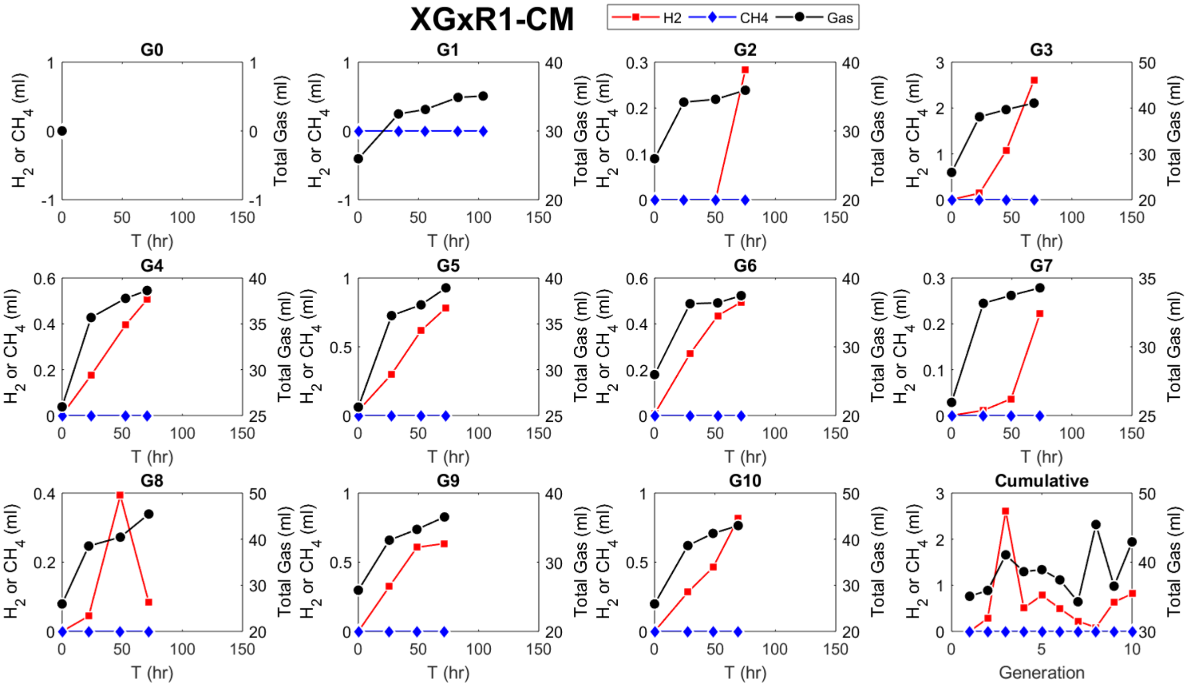

Supplementary Figure 6ac. The cumulative production of total gas, hydrogen (H_2), and methane (CH_4) in the first biological replicate of the chloramphenicol-treated consortia grown on xylan (XGxR1-CM) over the course of enrichment cultivation in hours (hr). The left y-axis is a scale for the volume of H_2 and CH_4 in milliliters and the right y-axis is a scale for the volume of total gas in milliliters. No measurements were taken for G0. The bottom right sub-Supplementary Figure shows the cumulative production of total gas, H_2 and CH_4 at the end of cultivation of each batch. Red squares represent H_2, blue diamonds represent CH_4, and black circles represent total gas. The cumulative volume of H_2, CH_4, and total gas in ml was calculated with headspace pressure and gas chromatograph concentration measurements as detailed in **Methods**. The total volume of the culturing vessel was 73 ml and the initial volume of the enrichment culture was 50 ml.

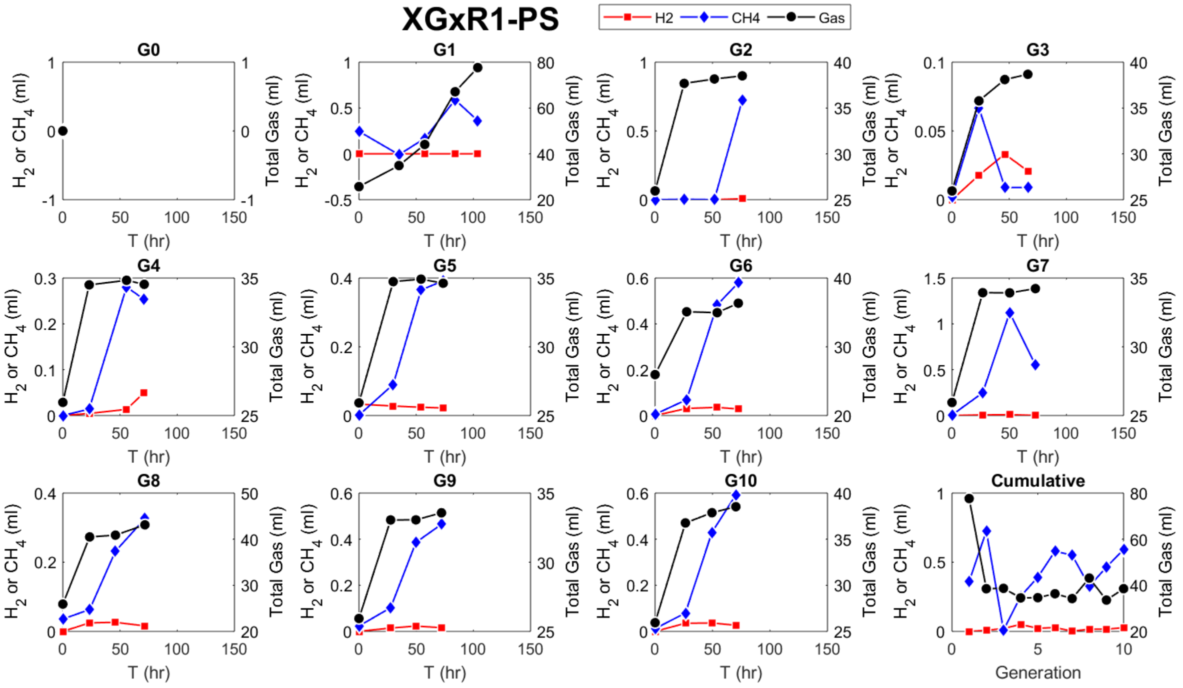

Supplementary Figure 6ad. The cumulative production of total gas, hydrogen (H_2), and methane (CH_4) in the first biological replicate of the penicillin and streptomycin-treated consortia grown on xylan (XGxR1-PS) over the course of enrichment cultivation in hours (hr). The left y-axis is a scale for the volume of H_2 and CH_4 in milliliters and the right y-axis is a scale for the volume of total gas in milliliters. No measurements were taken for G0. The bottom right sub-Supplementary Figure shows the cumulative production of total gas, H_2 and CH_4 at the end of cultivation of each batch. Red squares represent H_2, blue diamonds represent CH_4, and black circles represent total gas. The cumulative volume of H_2, CH_4, and total gas in ml was calculated with headspace pressure and gas chromatograph concentration measurements as detailed in **Methods**. The total volume of the culturing vessel was 73 ml and the initial volume of the enrichment culture was 50 ml.

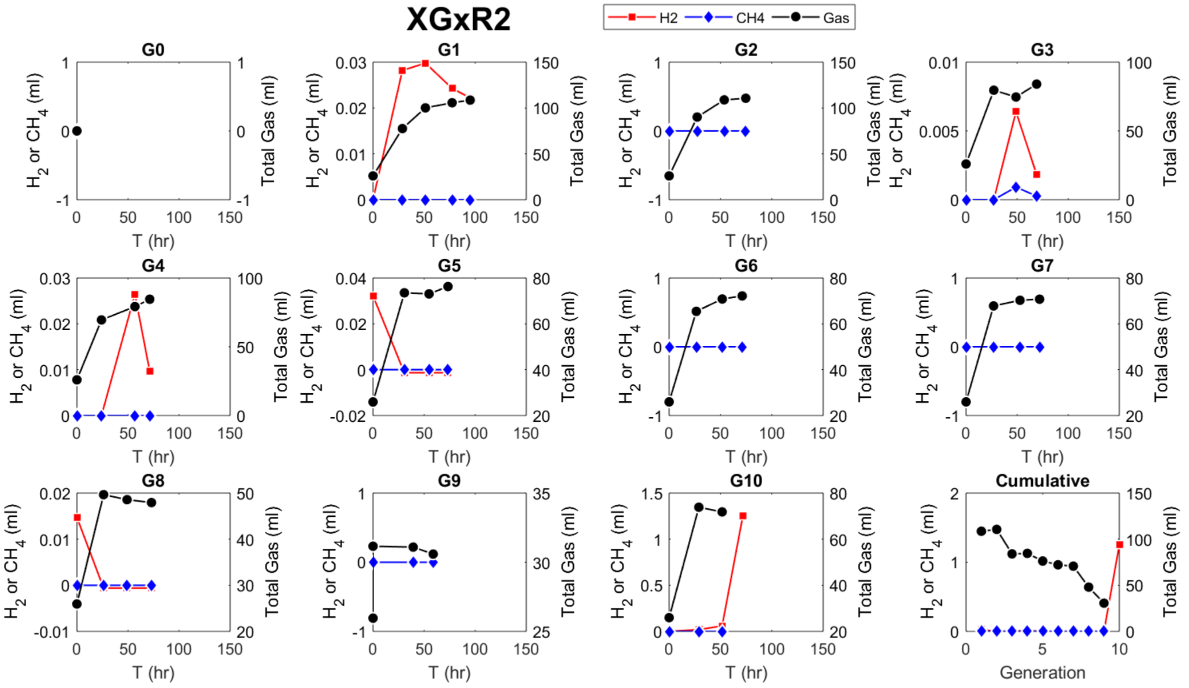

Supplementary Figure 6ae. The cumulative production of total gas, hydrogen (H_2), and methane (CH_4) in the second biological replicate of the antibiotics-free consortia grown on xylan (XGxR2) over the course of enrichment cultivation in hours (hr). The left y-axis is a scale for the volume of H_2 and CH_4 in milliliters and the right y-axis is a scale for the volume of total gas in milliliters. No measurements were taken for G0. The bottom right sub-Supplementary Figure shows the cumulative production of total gas, H_2 and CH_4 at the end of cultivation of each batch. Red squares represent H_2, blue diamonds represent CH_4, and black circles represent total gas. The cumulative volume of H_2, CH_4, and total gas in ml was calculated with headspace pressure and gas chromatograph concentration measurements as detailed in **Methods**. The total volume of the culturing vessel was 73 ml and the initial volume of the enrichment culture was 50 ml.

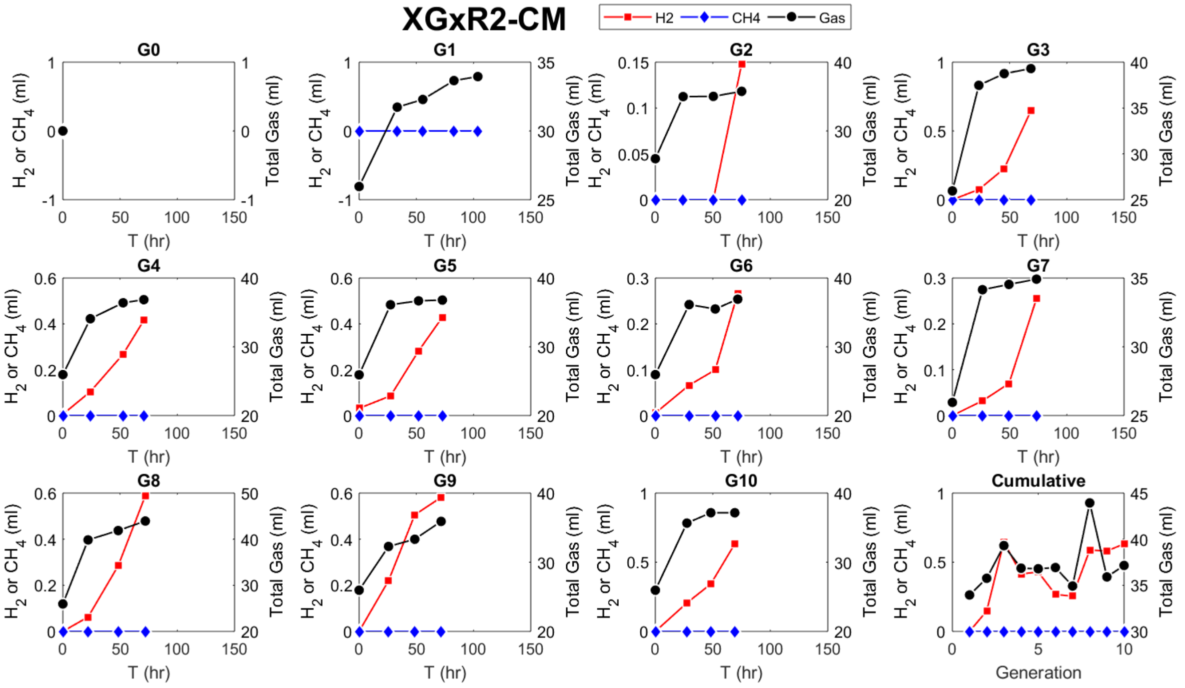

Supplementary Figure 6af. The cumulative production of total gas, hydrogen (H_2), and methane (CH_4) in the second biological replicate of the chloramphenicol-treated consortia grown on xylan (XGxR2-CM) over the course of enrichment cultivation in hours (hr). The left y-axis is a scale for the volume of H_2 and CH_4 in milliliters and the right y-axis is a scale for the volume of total gas in milliliters. No measurements were taken for G0. The bottom right sub-Supplementary Figure shows the cumulative production of total gas, H_2 and CH_4 at the end of cultivation of each batch. Red squares represent H_2, blue diamonds represent CH_4, and black circles represent total gas. The cumulative volume of H_2, CH_4, and total gas in ml was calculated with headspace pressure and gas chromatograph concentration measurements as detailed in **Methods**. The total volume of the culturing vessel was 73 ml and the initial volume of the enrichment culture was 50 ml.

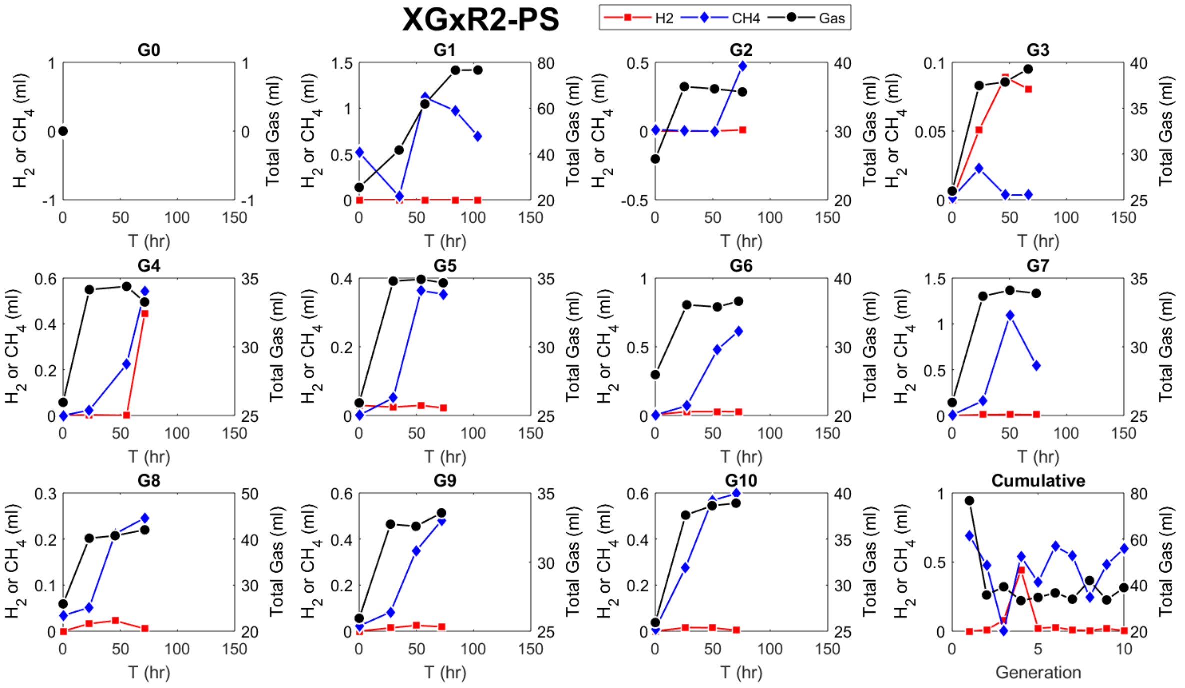

Supplementary Figure 6ag. The cumulative production of total gas, hydrogen (H_2), and methane (CH_4) in the second biological replicate of the penicillin and streptomycin-treated consortia grown on xylan (XGxR2-PS) over the course of enrichment cultivation in hours (hr). The left y-axis is a scale for the volume of H_2 and CH_4 in milliliters and the right y-axis is a scale for the volume of total gas in milliliters. No measurements were taken for G0. The bottom right sub-Supplementary Figure shows the cumulative production of total gas, H_2 and CH_4 at the end of cultivation of each batch. Red squares represent H_2, blue diamonds represent CH_4, and black circles represent total gas. The cumulative volume of H_2, CH_4, and total gas in ml was calculated with headspace pressure and gas chromatograph concentration measurements as detailed in **Methods**. The total volume of the culturing vessel was 73 ml and the initial volume of the enrichment culture was 50 ml.

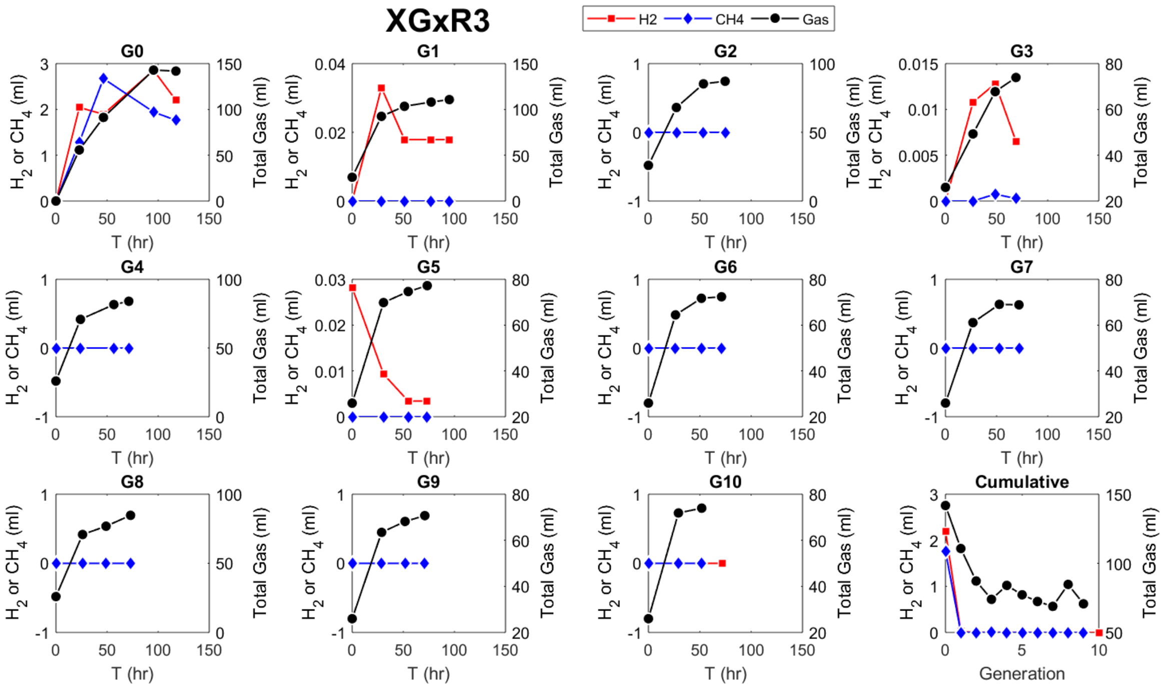

Supplementary Figure 6ah. The cumulative production of total gas, hydrogen (H_2), and methane (CH_4) in the third biological replicate of the antibiotics-free consortia grown on xylan (XGxR3) over the course of enrichment cultivation in hours (hr). The left y-axis is a scale for the volume of H_2 and CH_4 in milliliters and the right y-axis is a scale for the volume of total gas in milliliters. The bottom right sub-Supplementary Figure shows the cumulative production of total gas, H_2 and CH_4 at the end of cultivation of each batch. Red squares represent H_2, blue diamonds represent CH_4, and black circles represent total gas. The cumulative volume of H_2, CH_4, and total gas in ml was calculated with headspace pressure and gas chromatograph concentration measurements as detailed in **Methods**. The total volume of the culturing vessel was 73 ml and the initial volume of the enrichment culture was 50 ml.

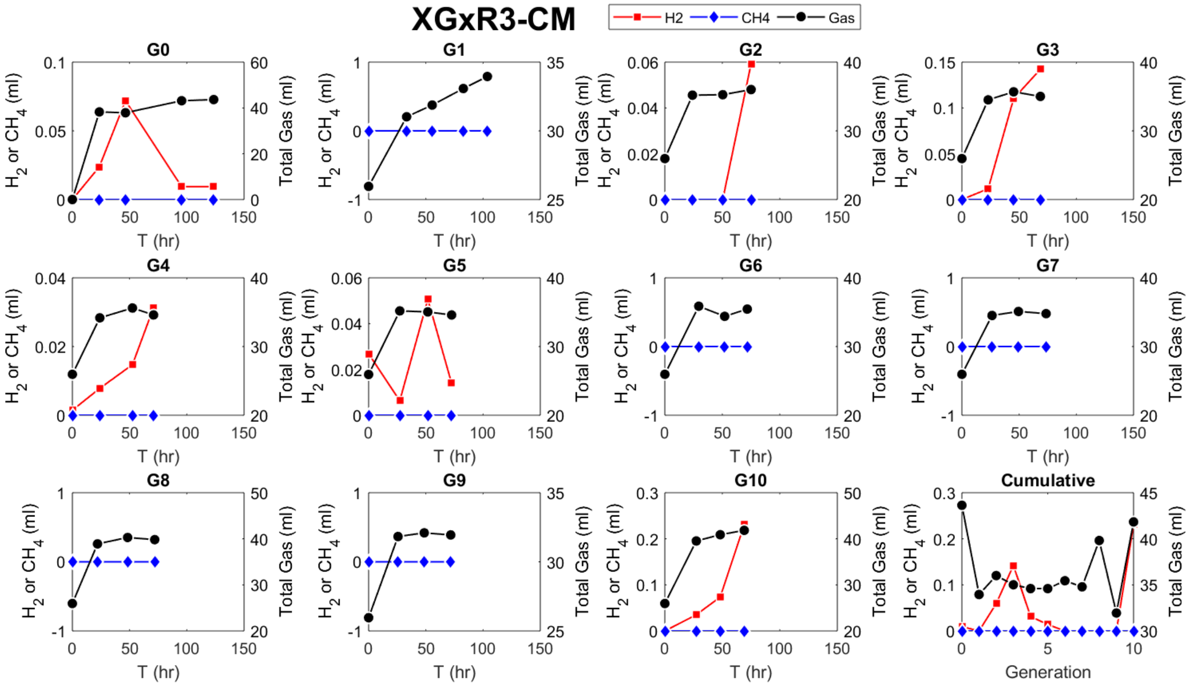

Supplementary Figure 6ai. The cumulative production of total gas, hydrogen (H_2), and methane (CH_4) in the third biological replicate of the chloramphenicol-treated consortia grown on xylan (XGxR3-CM) over the course of enrichment cultivation in hours (hr). The left y-axis is a scale for the volume of H_2 and CH_4 in milliliters and the right y-axis is a scale for the volume of total gas in milliliters. The bottom right sub-Supplementary Figure shows the cumulative production of total gas, H_2 and CH_4 at the end of cultivation of each batch. Red squares represent H_2, blue diamonds represent CH_4, and black circles represent total gas. The cumulative volume of H_2, CH_4, and total gas in ml was calculated with headspace pressure and gas chromatograph concentration measurements as detailed in **Methods**. The total volume of the culturing vessel was 73 ml and the initial volume of the enrichment culture was 50 ml.

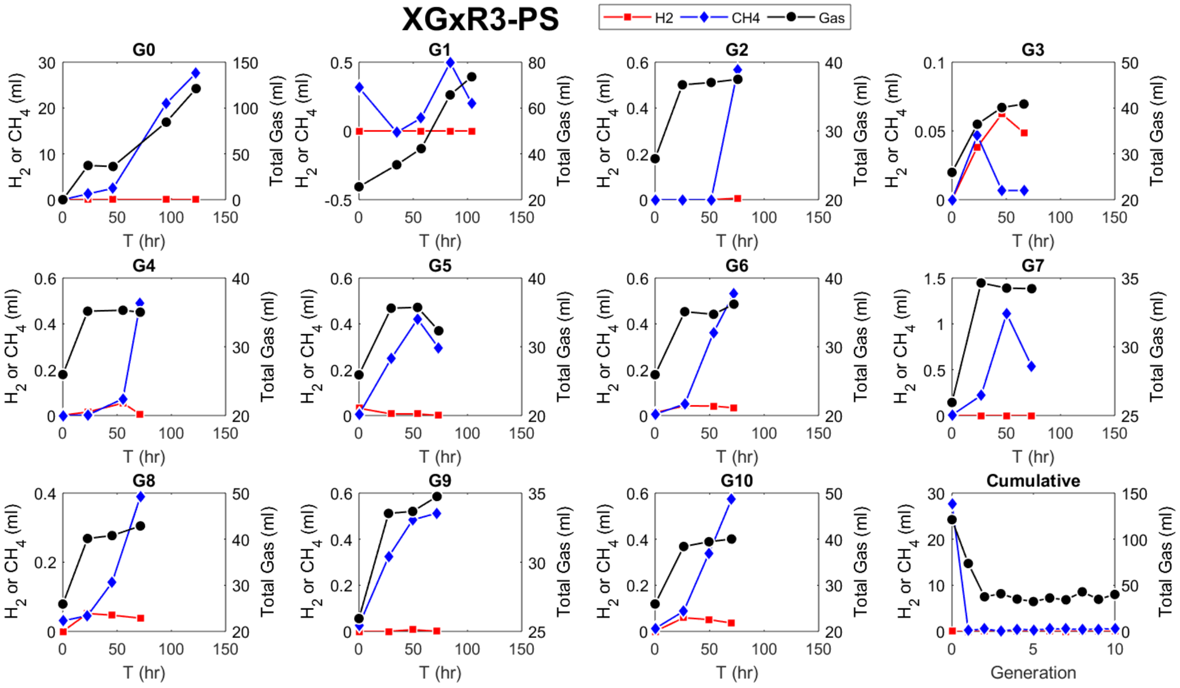

Supplementary Figure 6aj. The cumulative production of total gas, hydrogen (H_2), and methane (CH_4) in the third biological replicate of the penicillin and streptomycin-treated consortia grown on xylan (XGxR3-PS) over the course of enrichment cultivation in hours (hr). The left y-axis is a scale for the volume of H_2 and CH_4 in milliliters and the right y-axis is a scale for the volume of total gas in milliliters. The bottom right sub-Supplementary Figure shows the cumulative production of total gas, H_2 and CH_4 at the end of cultivation of each batch. Red squares represent H_2, blue diamonds represent CH_4, and black circles represent total gas. The cumulative volume of H_2, CH_4, and total gas in ml was calculated with headspace pressure and gas chromatograph concentration measurements as detailed in **Methods**. The total volume of the culturing vessel was 73 ml and the initial volume of the enrichment culture was 50 ml.

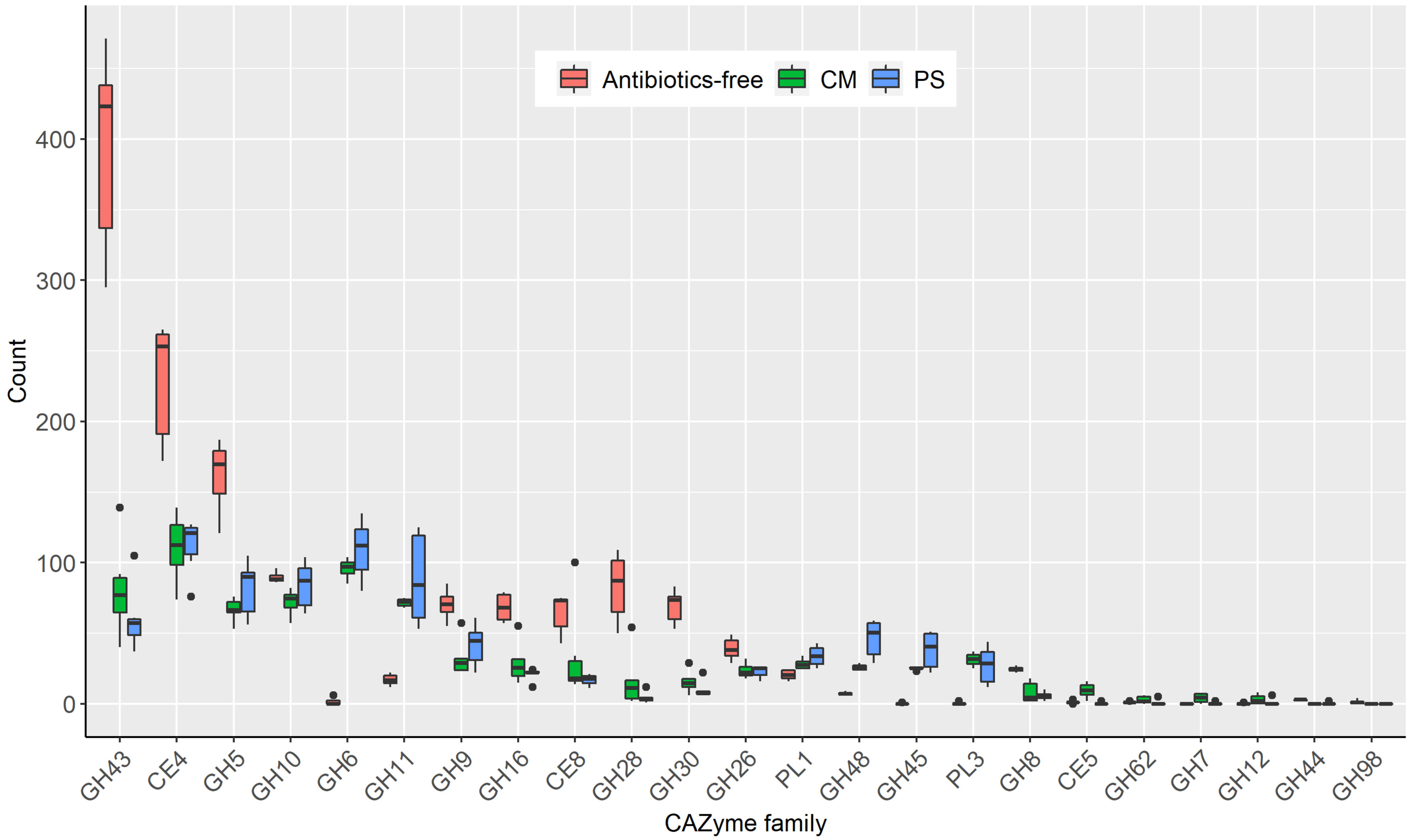

Supplementary Figure 7. Boxplot of the count of different carbohydrate-active enzyme (CAZyme) families identified in all assembled contigs generated from each consortium grown on plant substrates (alfalfa, bagasse, and reed canary grass) at generation 5 and 10. Antibiotics-free consortia were in red, chloramphenicol-treated (CM) consortia in green, and penicillin and streptomycin-treated (PS) consortia in blue. The upper limit of the boxes corresponds to the third quartile, the lower limit of the boxes corresponds to the first quartile, and the line between them corresponds to the median. The end of the upper whisker marks the smaller value of the maximum count and the third quartile plus 1.5 times the inter-quartile range (IQR). The end of the lower whisker marks the greater value of the minimum count and the first quartile minus 1.5 times the IQR.

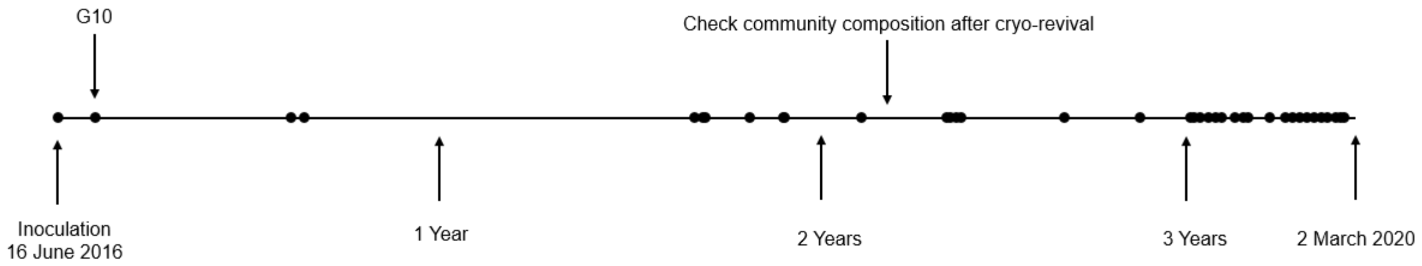

Supplementary Figure 8. Record of methane production from the "Alfalfa-PS" consortium over several years sub-cultivation. Each filled circle is a time point when methane production (> 1% in the headspace of sample vials) was verified by gas chromatography (GC) measurements.

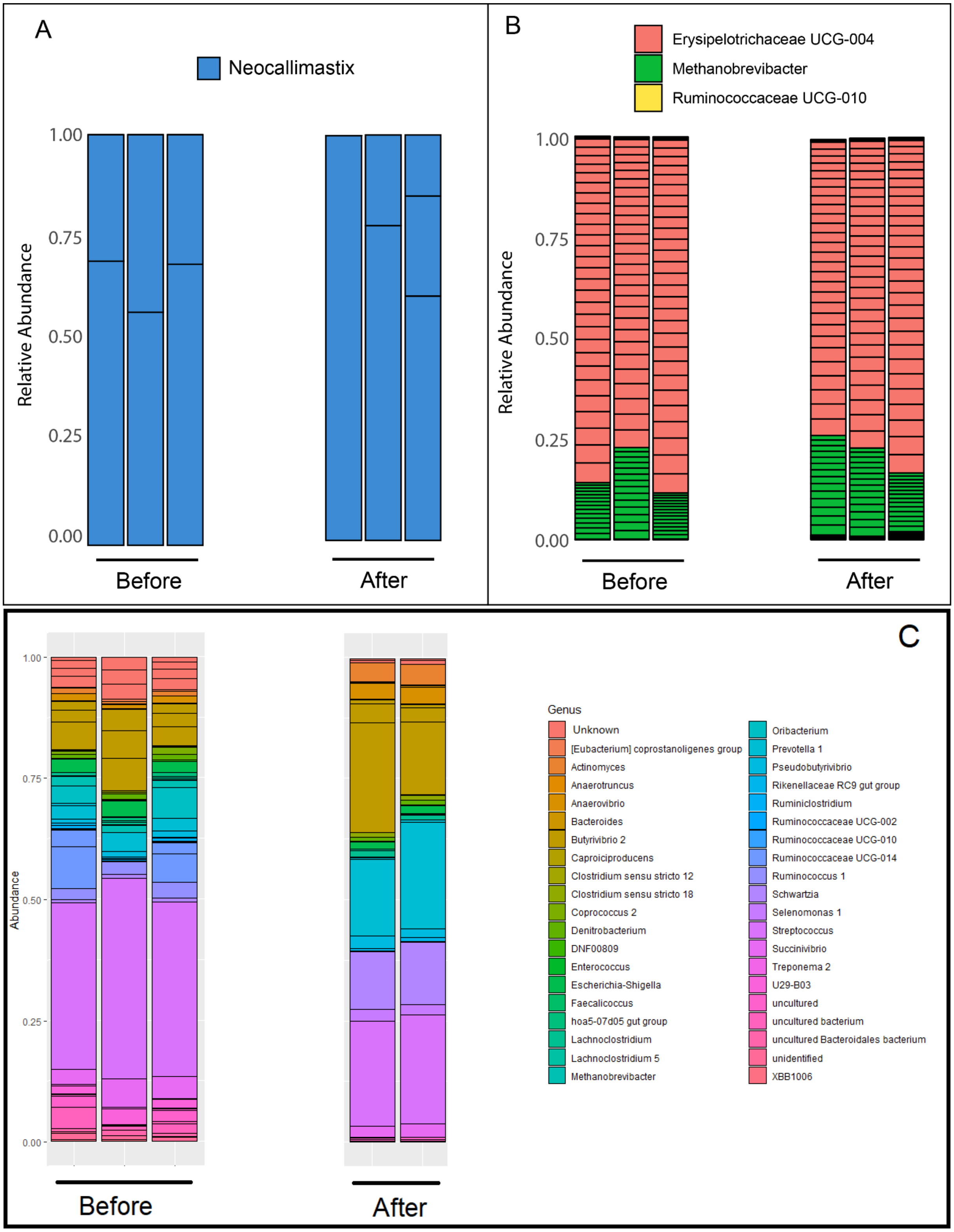

Supplementary Figure 9. Microbial community composition of consortia grown on alfalfa stems before and after cryopreservation at -80°C. Panel A shows the eukaryotic community composition in PS consortia evaluated by the internal transcribed spacer region 2 (ITS2); panel B shows the prokaryotic community composition in PS consortia evaluated by the V4 region of the 16S rRNA gene; panel C shows the prokaryotic community composition in antibiotics-free consortia evaluated by the V4 region of the 16S rRNA gene. The three bars adjacent to each other represent three biological replicates. Amplicon sequencing reads were processed in R using the package DADA2 version 1.8.0 and the figure was generated in R with the package phyloseq version 1.26.1.

Supplementary Table 1. Summary of the number of dereplicated metagenome-assembled genomes (MAGs) and eukaryotic MAGs (eukMAGs) by phylum in the source microbiome and the enrichment cultures (G5 and G10). Bacterial samples were untreated with antibiotics. PS samples are enrichment cultures treated with penicillin and streptomycin. CM samples are enrichment cultures treated with chloramphenicol.

Phylum	No. of MAGs	No. Detected in Pellets and G0	No. Enriched in Bacterial Samples	No. Enriched in PS Samples	No. Enriched in CM Samples
Firmicutes	531	506	101	15	4
Bacteroidetes	85	77	19	0	0
Euryarchaeota	25	21	10	11	0
Proteobacteria	23	22	1	1	0
Lentisphaerae	21	21	0	0	0
Actinobacteria	10	10	10	0	0
Verrucomicrobia	8	8	0	0	0
Cyanobacteria	7	7	0	0	0
Spirochaetes	6	6	1	0	0
Planctomycetes	2	2	0	0	0
Elusimicrobia	1	1	0	0	0
Total	719	681	142	27	4
eukMAGs	18	9	0	3	6

Supplementary Table 2. Summary statistics of the CheckM-assessed completeness and redundancy of genomes and metagenome-assembled genomes from this study and other sources that were used as reference genomes.

			CheckM Completeness			CheckM Redundancy		
**Reference**	**Source**	**Number of genomes/MAGs**	**Average**	**Min**	**Max**	**Average**	**Min**	**Max**
This study	Goat fecal pellets	719	91.8%	80%	100%	1.40%	0%	9.74%
Seshadri et al. 2018	Hungate Collection	493	99.3%	87.30%	100%	0.93%	0%	9.82%
Stewart et al. 2019	Cow rumen	4941	90.1%	80%	100%	2.03%	0%	10%
Mukherjee et al. 2017	GEBA	1003	99.4%	78.28%	100%	0.57%	0%	13.79%
Zou et al. 2019	Human gut	1520	96.8%	95.06%	100%	0.75%	0%	4.64%
Other genomes	NCBI RefSeq	221	98.5%	80.54%	100%	1.04%	0%	10%
	Total, excluding this study	8178						

Supplementary Table 3. Definition of cellulase, hemicellulase, pectinase, and esterase based on their catalytic domains. This table was curated based on the Carbohydrate-Active enZYmes Database (http://www.cazy.org/ accessed on 28 April, 2019). Note that enzymes containing glycoside hydrolases 5, 8, 44, and 51 are defined as both cellulases and hemicellulases.

Enyzme Class	Enzyme Name	Glycoside Hydrolase (GH)	Polysaccharide Lyase (PL)	Carbohydrate Esterase (CE)
Cellulase				
EC:3.2.1.4	1,4-Beta glucan hydrolysis	5,6,7,8,9,12,44,45,48,51,74,124		
EC:3.2.1.6	1,4-Beta/Alpha glucan hydrolysis	9		
Hemicellulase				
EC:3.2.1.8	1,4-Beta xylanase	5,8,10,11,16,26,30,43,44,51,62,98,141		
EC:3.2.1.32	1,3 beta xylanase	8,11,26		
Pectinase				
EC:3.2.1.15	Endo-polygalacturonase	28		
EC:4.2.2.2	Pectate lyase		1,2,3,9,10	
EC:4.2.2.9	Pectate disaccharide lyase		1,2,9	
Esterase				
EC:3.1.1.72	Acetyl Xylan esterase			1,2,3,4,5,6,7,12,15
EC:3.1.1.-	Pectin acetylesterase			13
EC:3.1.1.11	Pectin esterase			8

Supplementary Table 4. Results of Dunn's Test comparing the mean value of cellulase, hemicellulase, and pectinase/esterase counts between the taxonomic groups shown in Supplementary Figure 6. Groups sharing a letter are not significantly different (alpha = 0.05).

Group in Supp. Figure 6	Cellulase	Hemicellulase	Pectinase/Esterase
Actinobacteria	abc	abcdef	abcd
Bacteroidales_other	d	ag	ef
Butyrivibrio	ef	h	eghij
Clostridiales_other	ab	bcd	egh
Clostridium	abdgh	abdeg	i
Cyanobacteria	abc	cf	abc
Erysipelotrichaceae	adg	bcd	abd
Euryarchaeota	c	f	c
Firmicutes_other	abc	bcf	adf
Lachnospiraceae	eh	gi	eh
Lentisphaerae	dh	adeg	i
Others	abcdegh	abcdefg	abcdefghij
Paenibacillaceae	ef	hi	i
Planctomycetes	defgh	aeghi	abcdefghij
Prevotella	ef	h	gij
Proteobacteria	bc	f	bc
Ruminococcaceae	ag	de	gh
Ruminococcus	f	h	ij
Spirochaetes	adegh	abcdegi	abcd
Verrucomicrobia	abcdg	bcdf	defghj

Supplementary Table 5. The percentage of microorganisms in the source microbiome (fecal pellets diluted in blank media) that were present in batches G0, G1, G3, G5, G8, and G10.

Batch	G0			G1			G3			G5			G8			G10		
Replicate	R1	R2	R3	R1	R2	R3	R1	R2	R3	R1	R2	R3	R1	R2	R3	R1	R2	R3
**Alfalfa**	0.01%	0.03%	3.29%	0.60%	0.06%		0.04%		0.60%	0.59%	0.01%	0.59%	0.06%	0.58%	0.22%	0.59%	0.05%	0.03%
**Bagasse**	4.88%	0.24%	0.24%	2.24%	2.29%	2.24%	2.27%	2.29%	0	0.05%	2.29%	2.25%	0.05%	2.28%	2.24%	0.05%	0.05%	2.24%
**RCG**	0.05%	0.05%	0.06%	0.55%	2.84%	0.60%	0.03%	0.03%	0.58%	2.79%	2.84%	0	2.27%	2.27%	0.55%	0.58%	2.29%	2.79%
**Xylan**	4.46%	0	3.23%	0	0	0	0	0	0	0	0	0	0	0	0	0	0	0

Supplementary Table 6a. The average (n = 3) relative abundance of the top 20 most abundant 16S-V4 amplicon sequence variants (ASV) Clusters and their taxonomy in antibiotics-free consortia grown on alfalfa stems at generation 10. None of the ASV Clusters were detected in the fecal pellets.

ASV Cluster ID	Phylum	Class	Order	Family	Genus	Relative Abundance	Comparison to Pellets
5	Firmicutes	Bacilli	Lactobacillales	Streptococcaceae	Streptococcus	29.0%	Not present
2	Firmicutes	Clostridia	Clostridiales	Lachnospiraceae	Butyrivibrio	10.6%	Not present
56	Firmicutes	Clostridia	Clostridiales	Ruminococcaceae	Group UCG-014	8.0%	Not present
77	Proteobacteria	γ-Proteobacteria	Aeromonadales	Succinivibrionaceae	Succinivibrio	4.7%	Not present
20	Firmicutes	Clostridia	Clostridiales	Lachnospiraceae	Oribacterium	4.3%	Not present
10	Bacteroidetes	Bacteroidia	Bacteroidales	Prevotellaceae	Prevotella	3.5%	Not present
42	Firmicutes	Clostridia	Clostridiales	Lachnospiraceae	Unidentified	3.1%	Not present
13	Firmicutes	Clostridia	Clostridiales	Lachnospiraceae	Unidentified	3.0%	Not present
11	Proteobacteria	γ-Proteobacteria	Enterobacteriales	Enterobacteriaceae	Escherichia/Shigella	3.0%	Not present
45	Bacteroidetes	Bacteroidia	Bacteroidales	Unidentified	Unidentified	2.7%	Not present
31	Bacteroidetes	Bacteroidia	Bacteroidales	Rikenellaceae	Group U29-B03	2.6%	Not present
116	Bacteroidetes	Bacteroidia	Bacteroidales	p-251-o5	Unidentified	2.5%	Not present
16	Firmicutes	Clostridia	Clostridiales	Ruminococcaceae	Ruminococcus	2.1%	Not present
58	Firmicutes	Negativicutes	Selenomonadales	Veillonellaceae	Anaerovibrio	2.1%	Not present
41	Firmicutes	Clostridia	Clostridiales	Lachnospiraceae	Butyrivibrio	2.0%	Not present
64	Firmicutes	Clostridia	Clostridiales	Clostridiaceae_1	Clostridium	1.8%	Not present
34	Firmicutes	Clostridia	Clostridiales	Lachnospiraceae	Group AC2044	1.6%	Not present
136	Firmicutes	Bacilli	Lactobacillales	Streptococcaceae	Streptococcus	1.4%	Not present
348	Actinobacteria	Coriobacteriia	Coriobacteriales	Eggerthellaceae	Denitrobacterium	1.1%	Not present
122	Firmicutes	Clostridia	Clostridiales	Lachnospiraceae	Butyrivibrio	1.1%	Not present
					Sum	90.0%	

Supplementary Table 6b. The average (n = 3) relative abundance of the top 20 most abundant 16S-V4 amplicon sequence variants (ASV) Clusters and their taxonomy in antibiotics-free consortia grown on <u>bagasse</u> at generation 10. When the ASV Cluster is present in fecal pellets, the "Enrichment compared to pellets" represents the ratio of the relative abundance in the consortia to that in pellets.

ASV Cluster ID	Phylum	Class	Order	Family	Genus	Relative Abundance	Enrichment compared to Pellets
34	Firmicutes	Clostridia	Clostridiales	Lachnospiraceae	Group AC2044	17.3%	Not present
2	Firmicutes	Clostridia	Clostridiales	Lachnospiraceae	Butyrivibrio	14.8%	Not present
3	Firmicutes	Clostridia	Clostridiales	Lachnospiraceae	Pseudobutyrivibrio	14.4%	Not present
10	Bacteroidetes	Bacteroidia	Bacteroidales	Prevotellaceae	Prevotella	11.0%	Not present
11	Proteobacteria	γ-Proteobacteria	Enterobacteriales	Enterobacteriaceae	Escherichia/Shigella	5.3%	Not present
5	Firmicutes	Bacilli	Lactobacillales	Streptococcaceae	Streptococcus	5.0%	Not present
16	Firmicutes	Clostridia	Clostridiales	Ruminococcaceae	Ruminococcus	4.4%	Not present
20	Firmicutes	Clostridia	Clostridiales	Lachnospiraceae	Oribacterium	3.9%	Not present
13	Firmicutes	Clostridia	Clostridiales	Lachnospiraceae	Unidentified	3.1%	Not present
30	Firmicutes	Clostridia	Unidentified	Unidentified	Unidentified	2.3%	Not present
104	Tenericutes	Mollicutes	Mollicutes_RF39	Unidentified	Unidentified	1.9%	Not present
64	Firmicutes	Clostridia	Clostridiales	Clostridiaceae_1	Clostridium	1.5%	Not present
70	Bacteroidetes	Bacteroidia	Bacteroidales	p-251-o5	Unidentified	1.5%	56x
336	Firmicutes	Clostridia	Clostridiales	Ruminococcaceae	Ruminococcus	1.5%	Not present
207	Proteobacteria	δ-Proteobacteria	Desulfovibrionales	Desulfovibrionaceae	Desulfovibrio	1.4%	Not present
41	Firmicutes	Clostridia	Clostridiales	Lachnospiraceae	Butyrivibrio	1.1%	Not present
27	Firmicutes	Clostridia	Clostridiales	Ruminococcaceae	Ruminococcus	1.0%	0.5x
39	Firmicutes	Clostridia	Clostridiales	Clostridiaceae_1	Clostridium	0.9%	Not present
228	Firmicutes	Negativicutes	Selenomonadales	Veillonellaceae	Schwartzia	0.9%	Not present
348	Actinobacteria	Coriobacteriia	Coriobacteriales	Eggerthellaceae	Denitrobacterium	0.9%	Not present
					Sum	94.1%	

Supplementary Table 6c. The average (n = 3) relative abundance of the top 20 most abundant 16S-V4 amplicon sequence variants (ASV) Clusters and their taxonomy in antibiotics-free consortia grown on reed canary grass at generation 10. When the ASV Cluster is present in fecal pellets, the "Enrichment compared to pellets" represents the ratio of the relative abundance in the consortia to that in pellets.

ASV Cluster ID	Phylum	Class	Order	Family	Genus	Relative Abundance	Enrichment compared to Pellets
5	Firmicutes	Bacilli	Lactobacillales	Streptococcaceae	Streptococcus	28.2%	Not present
13	Firmicutes	Clostridia	Clostridiales	Lachnospiraceae	Unidentified	10.7%	Not present
2	Firmicutes	Clostridia	Clostridiales	Lachnospiraceae	Butyrivibrio	10.6%	Not present
16	Firmicutes	Clostridia	Clostridiales	Ruminococcaceae	Ruminococcus	4.5%	Not present
64	Firmicutes	Clostridia	Clostridiales	Clostridiaceae	Clostridium	4.4%	Not present
136	Firmicutes	Bacilli	Lactobacillales	Streptococcaceae	Streptococcus	4.4%	Not present
10	Bacteroidetes	Bacteroidia	Bacteroidales	Prevotellaceae	Prevotella	4.2%	Not present
3	Firmicutes	Clostridia	Clostridiales	Lachnospiraceae	Pseudobutyrivibrio	3.3%	Not present
42	Firmicutes	Clostridia	Clostridiales	Lachnospiraceae	Unidentified	3.3%	Not present
11	Proteobacteria	γ-Proteobacteria	Enterobacteriales	Enterobacteriaceae	Escherichia/Shigella	2.7%	Not present
12	Euryarchaeota	Methanobacteria	Methanobacteriales	Methanobacteriaceae	Methanobrevibacter	1.9%	4.5
171	Firmicutes	Clostridia	Clostridiales	Lachnospiraceae	Butyrivibrio	1.9%	Not present
215	Firmicutes	Clostridia	Clostridiales	Lachnospiraceae	Group XPB1014	1.6%	Not present
77	Proteobacteria	γ-Proteobacteria	Aeromonadales	Succinivibrionaceae	Succinivibrio	1.6%	Not present
27	Firmicutes	Clostridia	Clostridiales	Ruminococcaceae	Ruminococcus	1.6%	0.9
203	Firmicutes	Negativicutes	Selenomonadales	Veillonellaceae	Anaerovibrio	1.4%	Not present
20	Firmicutes	Clostridia	Clostridiales	Lachnospiraceae	Oribacterium	1.3%	Not present
49	Bacteroidetes	Bacteroidia	Bacteroidales	Rikenellaceae	Gut group RC9	1.1%	Not present
34	Firmicutes	Clostridia	Clostridiales	Lachnospiraceae	Group AC2044	1.1%	Not present
228	Firmicutes	Negativicutes	Selenomonadales	Veillonellaceae	Schwartzia	1.1%	Not present
					Sum	90.9%	

Supplementary Table 6d. The average (n = 3) relative abundance of the top 20 most abundant 16S-V4 amplicon sequence variants (ASV) Clusters and their taxonomy in antibiotics-free consortia grown on xylan at generation 10. None of the ASV Clusters were detected in the fecal pellets.

ASV Cluster ID	Phylum	Class	Order	Family	Genus	Relative Abundance	Pellets
71	Firmicutes	Negativicutes	Selenomonadales	Veillonellaceae	Selenomonas	70.7%	Not present
17	Firmicutes	Bacilli	Lactobacillales	Enterococcaceae	Enterococcus	14.8%	Not present
374	Firmicutes	Negativicutes	Selenomonadales	Veillonellaceae	Selenomonas	5.9%	Not present
14	Firmicutes	Clostridia	Clostridiales	Clostridiaceae	Clostridium	5.2%	Not present
28	Firmicutes	Clostridia	Clostridiales	Ruminococcaceae	Caproiciproducens	2.6%	Not present
5	Firmicutes	Bacilli	Lactobacillales	Streptococcaceae	Streptococcus	0.7%	Not present
					Sum	100.0%	

Supplementary Table 6e. The average (n = 3) relative abundance of all 16S-V4 amplicon sequence variants (ASV) Clusters and their taxonomy in penicillin and streptomycin-treated consortia at generation 10. When the ASV Cluster is present in fecal pellets, the "Enrichment compared to pellets" represents the ratio of the relative abundance in the consortia to that in pellets.

ASV Cluster ID	Phylum	Class	Order	Family	Genus	Relative Abundance	Enrichment compared to Pellets
**Consortia enriched on alfalfa stems, treated with penicillin and streptomycin**							
11	Proteobacteria	γ-Proteobacteria	Enterobacteriales	Enterobacteriaceae	Escherichia/Shigella	31.8%	Not Present
12	Euryarchaeota	Methanobacteria	Methanobacteriales	Methanobacteriaceae	Methanobrevibacter	30.0%	69x
81	Firmicutes	Erysipelotrichia	Erysipelotrichales	Erysipelotrichaceae	Group UCG-004	24.8%	Not Present
18	Firmicutes	Erysipelotrichia	Erysipelotrichales	Erysipelotrichaceae	Group UCG-004	5.2%	Not Present
23	Firmicutes	Clostridia	Clostridiales	Ruminococcaceae	Group UCG-010	3.5%	Not Present
206	Euryarchaeota	Methanobacteria	Methanobacteriales	Methanobacteriaceae	Methanobrevibacter	1.6%	Not Present
166	Euryarchaeota	Methanobacteria	Methanobacteriales	Methanobacteriaceae	Methanosphaera	1.6%	Not Present
146	Euryarchaeota	Methanobacteria	Methanobacteriales	Methanobacteriaceae	Methanobrevibacter	1.2%	Not Present
82	Euryarchaeota	Thermoplasmata	Methanomassiliicoccales	Methanomethylophilaceae	Unidentified	0.2%	Not Present
109	Euryarchaeota	Thermoplasmata	Methanomassiliicoccales	Methanomethylophilaceae	Unidentified	0.1%	Not Present
538	Euryarchaeota	Thermoplasmata	Methanomassiliicoccales	Methanomethylophilaceae	Unidentified	0.1%	Not Present
**Consortia enriched on bagasse, treated with penicillin and streptomycin**							
12	Euryarchaeota	Methanobacteria	Methanobacteriales	Methanobacteriaceae	Methanobrevibacter	76%	177x
18	Firmicutes	Erysipelotrichia	Erysipelotrichales	Erysipelotrichaceae	Group UCG-004	15%	Not Present
206	Euryarchaeota	Methanobacteria	Methanobacteriales	Methanobacteriaceae	Methanobrevibacter	9%	Not Present
**Consortia enriched on reed canary grass, treated with penicillin and streptomycin**							
18	Firmicutes	Erysipelotrichia	Erysipelotrichales	Erysipelotrichaceae	Group UCG-004	39.0%	Not Present
12	Euryarchaeota	Methanobacteria	Methanobacteriales	Methanobacteriaceae	Methanobrevibacter	37.8%	87x
81	Firmicutes	Erysipelotrichia	Erysipelotrichales	Erysipelotrichaceae	Group UCG-004	21.5%	Not Present
22	Firmicutes	Erysipelotrichia	Erysipelotrichales	Erysipelotrichaceae	Group UCG-004	0.6%	Not Present
206	Euryarchaeota	Methanobacteria	Methanobacteriales	Methanobacteriaceae	Methanobrevibacter	0.6%	Not Present
52	Euryarchaeota	Methanobacteria	Methanobacteriales	Methanobacteriaceae	Methanosphaera	0.5%	Not Present
**Consortia enriched on xylan, treated with penicillin and streptomycin**							
68	Firmicutes	Clostridia	Clostridiales	Ruminococcaceae	Group UCG-010	46.8%	Not Present
18	Firmicutes	Erysipelotrichia	Erysipelotrichales	Erysipelotrichaceae	Group UCG-004	21.2%	Not Present
12	Euryarchaeota	Methanobacteria	Methanobacteriales	Methanobacteriaceae	Methanobrevibacter	20.2%	47x
206	Euryarchaeota	Methanobacteria	Methanobacteriales	Methanobacteriaceae	Methanobrevibacter	10.1%	Not Present
52	Euryarchaeota	Methanobacteria	Methanobacteriales	Methanobacteriaceae	Methanosphaera	1.0%	117x
689	Firmicutes	Clostridia	Clostridiales	Ruminococcaceae	Group UCG-010	0.3%	Not Present
23	Firmicutes	Clostridia	Clostridiales	Ruminococcaceae	Group UCG-010	0.3%	Not Present

Supplementary Table 6f. The average (n = 3) relative abundance of all 16S-V4 amplicon sequence variants (ASV) Clusters and their taxonomy in chloramphenicol-treated consortia at generation 10. None of the ASV Clusters were detected in the fecal pellets. Note that no 16S amplicon libraries were successfully constructed from chloramphenicol-treated consortia grown on alfalfa stems and xylan at generation 10, indicating the lack of prokaryotic community members.

ASV Cluster ID	Phylum	Class	Order	Family	Genus	Relative Abundance
**Consortia enriched on bagasse, treated with penicillin and streptomycin**						
8	Actinobacteria	Actinobacteria	Streptomycetales	Streptomycetaceae	Streptomyces	55.2%
6	Firmicutes	Bacilli	Bacillales	Bacillaceae	Unidentified	44.8%
**Consortia enriched on bagasse, treated with penicillin and streptomycin**						
26	Firmicutes	Bacilli	Bacillales	Staphylococcaceae	Staphylococcus	62.6%
9	Firmicutes	Bacilli	Lactobacillales	Leuconostocaceae	Weissella	18.2%
147	Actinobacteria	Actinobacteria	Micrococcales	Microbacteriaceae	Leucobacter	7.4%
50	Actinobacteria	Actinobacteria	Micrococcales	Microbacteriaceae	Frigoribacterium	5.3%
191	Actinobacteria	Actinobacteria	Micrococcales	Sanguibacteraceae	Sanguibacter	2.1%
155	Actinobacteria	Actinobacteria	Corynebacteriales	Nocardiaceae	Rhodococcus	1.8%

Supplementary Table 7. The relative abundance of the eleven most abundant internal transcribed spacer 2 (ITS2) amplicon sequence variant (ASV) Clusters found in the source microbiota (goat fecal pellets) and the 15 metagenome samples from which at least one high-quality eukaryotic metagenome-assembled genome (eukMAG) were reconstructed. The ASV clusters belong to the anaerobic fungi genera *Neocallimastix*, *Piromyces*, and *Caecomyces*, the Ascomycetous genus *Saccharomyces*, and the Basidiomycetous genus *Ustilago*. The eukMAGs and the putative corresponding ASV Cluster were highlighted by green for *Neocallimastix*, blue for *Piromyces*, and orange for *Caecomyces*.

ASV Cluster ID	Genus	Pellets	AG0R1 - PS	AG0R2 - CM	AG5R2 - CM	AG10R1 - CM	BG0R3 - PS	BG5R3 - PS	BG10R3 - PS	BG5R2 - CM	BG10R3 - CM	RG0R3 - PS	RG10R3 - PS	RG0R2 - CM	RG5R2 - CM	RG10R2 - CM	XG0R3 - PS
1	Neocallimastix	5.7%	18.0%	3.3%	40.9%	18.0%	2.3%	34.9%	32.5%	69.7%	13.5%	16.7%	33.4%	26.0%	36.1%	36.1%	5.8%
2	Neocallimastix	4.7%	18.5%	4.5%	49.7%	76.0%	8.7%	56.2%	61.9%	20.2%	86.2%	35.0%	59.4%	53.8%	62.1%	62.1%	5.1%
7	Neocallimastix	1.7%	2.7%	1.1%	9.4%	5.3%	1.6%	5.6%	5.7%	10.1%	0.0%	0.4%	0.4%	2.4%	1.8%	1.8%	1.4%
Total Neocallimastix		12.1%	39.3%	8.8%	100%	99.3%	12.6%	96.7%	100.0%	99.9%	99.8%	52.1%	93.2%	82.2%	100%	100%	12.2%
4	Piromyces	31.7%	45.7%	65.7%	0.0%	0.0%	27.8%	3.1%	0.0%	0.0%	0.0%	38.9%	3.1%	13.0%	0.0%	0.0%	0.1%
6	Piromyces	9.1%	14.7%	24.9%	0.0%	0.0%	15.8%	0.2%	0.0%	0.0%	0.0%	5.9%	3.7%	4.1%	0.0%	0.0%	0.0%
Total Piromyces		40.9%	60.3%	90.6%	0.0%	0.0%	43.6%	3.3%	0.0%	0.0%	0.0%	44.8%	6.8%	17.1%	0.0%	0.0%	0.1%
3	Caecomyces	25.7%	0.4%	0.5%	0.0%	0.0%	37.4%	0.0%	0.0%	0.0%	0.0%	2.3%	0.0%	0.6%	0.0%	0.0%	87.5%
9	Caecomyces	3.0%	0.0%	0.0%	0.0%	0.0%	0.3%	0.0%	0.0%	0.0%	0.0%	0.1%	0.0%	0.0%	0.0%	0.0%	0.2%
10	Caecomyces	1.0%	0.0%	0.0%	0.0%	0.0%	0.0%	0.0%	0.0%	0.0%	0.0%	0.0%	0.0%	0.0%	0.0%	0.0%	0.1%
14	Caecomyces	0.5%	0.0%	0.0%	0.0%	0.0%	0.1%	0.0%	0.0%	0.0%	0.0%	0.7%	0.0%	0.0%	0.0%	0.0%	0.0%
Total Caecomyces		30.1%	0.4%	0.5%	0.0%	0.0%	37.9%	0.0%	0.0%	0.0%	0.0%	3.1%	0.0%	0.6%	0.0%	0.0%	87.7%
5	Saccharomyces	12.3%	0.0%	0.0%	0.0%	0.0%	0.0%	0.0%	0.0%	0.0%	0.0%	0.0%	0.0%	0.0%	0.0%	0.0%	0.0%
12	Ustilago	1.9%	0.0%	0.00%	0.0%	0.0%	0.0%	0.0%	0.0%	0.0%	0.0%	0.0%	0.0%	0.0%	0.0%	0.0%	0.0%
Neocallimastix eukMAG			bin.53		bin.1	bin.2		bin.9	bin.3	bin.2	bin.4	bin.10_13	bin.3	bin.24	bin.2	bin.4	
Piromyces eukMAG			bin.50	bin.1_171			bin.50					bin.6		bin.197			
Caecomyces eukMAG																	bin.26

Supplementary Table 8. Major biopolymers that compose the four substrates used for enrichment in this study. The most abundant component in each type of substrate is shown in bold font.

Component	Alfalfa Stem	Bagasse	Reed Canary Grass	Xylan
Cellulose	29-39%	42-46%	**28-43%**	0
Hemicellulose	8-10%	25-33%	22-28%	**100%**
Lignin	8-13%	**20-24%**	6-10%	0
Pectin	**8-15%**	~0	~0	0
Ashes	~2%	3-5%	~3%	0

Supplementary Table 9. Functional redundancy represented by the number of MAGs under each metabolic category in antibiotics-free consortia at G10 grown on alfalfa stems ("AG10R3"), bagasse ("BG10R2"), reed canary grass ("RG10R3"), and xylan ("XG10R1"). In the parenthesis are the number of MAGs that are > 1% in relative abundance as shown in Figure 3. MAGs are considered to possess cellulase, hemicellulase, pectinase/esterasae only if there are at least two corresponding carbohydrate-active enzymes present in the genome bin. The other pathways have to be at least 75% complete to be counted as present in a MAG.

	**AG10R3**	**BG10R2**	**RG10R3**	**XG10R1**
**Total number of MAGs**	81 (11)	51 (12)	77 (16)	4 (2)
**Hydrolysis**				
Cellulase	37 (5)	32 (9)	44 (10)	0 (0)
Hemicellulase	52 (7)	37 (10)	51 (11)	3 (1)
Pectinase/Esterase	57 (7)	40 (11)	58 (11)	2 (1)
Starch Degradation	66 (10)	43 (12)	64 (14)	3 (1)
**Utilization of Sugars (Fermentation)**				
Hexoses				
Glucose	78 (11)	51 (12)	76 (16)	3 (1)
Galactose	61 (10)	37 (10)	58 (13)	3 (1)
Mannose	54 (8)	27 (6)	46 (9)	4 (2)
Pentoses				
Xylose	68 (8)	46 (12)	63 (12)	2 (1)
Arabinose	8 (2)	2 (2)	5 (1)	2 (1)
Uronic Acids				
Galacturonate	30 (3)	20 (6)	35 (6)	2 (1)
Glucuronate	0 (0)	0 (0)	0 (0)	0 (0)
Deoxyhexoses				
Fucose	10 (3)	10 (4)	18 (3)	0 (0)
Rhamnose	27 (3)	16 (4)	25 (2)	1 (0)
**Fermentation Products**				
H_2	37 (3)	24 (6)	38 (6)	1 (1)
Formate	60 (8)	34 (10)	52 (13)	2 (1)
Acetate	64 (9)	44 (11)	64 (13)	2 (0)
Lactate	66 (10)	38 (11)	60 (14)	4 (2)
Propionate	11 (1)	6 (1)	12 (2)	1 (1)
Butyrate	28 (4)	23 (4)	33 (8)	1 (0)
Ethanol	23 (5)	12 (4)	17 (5)	1 (0)
**Methanogenesis**				
CO_2 Methanogenesis	4 (0)	1 (0)	3 (1)	0 (0)
Acetate Methanogenesis	6 (1)	1 (0)	4 (1)	0 (0)
Methanol Methanogenesis	3 (1)	1 (0)	2 (0)	0 (0)
Amine Methanogenesis	0 (0)	0 (0)	0 (0)	0 (0)

Supplementary Table 10. Statistical test results comparing the average number of each type of carbohydrate-active enzyme (CAZyme) between antibiotics-free, chloramphenicol-treated (CM), and penicillin & streptomycin-treated (PS) consortia grown on plant substrates from generations 5 and 10. The null hypothesis for analysis of variance (ANOVA) is that there is no difference in the number of CAZyme between the three antibiotics treatments. Tukey's Honestly Significant Difference (HSD) test was performed between each of the three possible pairs. P-values lower than 0.05 are italicized.

	p-value			
		Tukey's HSD		
CAZyme family	ANOVA	Antibiotics-free vs. CM	Antibiotics-free vs. PS	CM vs. PS
GH5	*7.65E-07*	*1.17E-06*	*9.92E-06*	3.82E-01
GH6	*4.34E-10*	*5.65E-09*	*8.66E-10*	1.97E-01
GH7	*5.60E-03*	*8.80E-03*	9.55E-01	*1.57E-02*
GH8	*9.03E-06*	*7.44E-05*	*1.52E-05*	6.42E-01
GH9	*3.31E-04*	*3.30E-04*	*4.27E-03*	4.15E-01
GH12	1.46E-01	1.37E-01	8.22E-01	3.47E-01
GH44	*2.24E-06*	*4.20E-06*	*1.83E-05*	6.48E-01
GH45	*1.95E-06*	*1.81E-04*	*1.49E-06*	*3.01E-02*
GH48	*1.43E-06*	*2.23E-03*	*8.99E-07*	*1.16E-03*
GH10	*4.74E-02*	*4.31E-02*	6.99E-01	1.84E-01
GH11	*3.66E-05*	*6.02E-04*	*3.94E-05*	3.31E-01
GH16	*2.31E-06*	*2.84E-05*	*3.41E-06*	4.23E-01
GH26	*3.59E-04*	*1.16E-03*	*7.96E-04*	9.80E-01
GH30	*9.79E-09*	*8.78E-08*	*2.28E-08*	5.29E-01
GH43	*4.62E-09*	*3.19E-08*	*1.34E-08*	7.44E-01
GH62	2.54E-01	4.00E-01	9.53E-01	2.64E-01
GH98	*2.59E-02*	*4.47E-02*	*4.47E-02*	1.00E+00
GH28	*3.22E-06*	*3.44E-05*	*5.03E-06*	4.98E-01
PL1	*1.70E-03*	5.29E-02	*1.24E-03*	1.74E-01
PL3	*1.57E-05*	*2.65E-05*	*1.19E-04*	6.83E-01
CE4	*8.45E-06*	*2.67E-05*	*3.08E-05*	9.96E-01
CE5	*1.85E-04*	*8.40E-04*	8.88E-01	*3.42E-04*
CE8	*4.64E-03*	5.45E-02	*3.80E-03*	3.90E-01

Supplementary Table 11. Summary statistics of the number of CAZyme gene clusters (CGC) and polysaccharide utilization loci (PUL) among the metagenome-assembled genomes (MAG) present in antibiotics-free enrichment cultures at generation 10.

	**AG10R3**	**BG10R2**	**RG10R3**	**XG10R1**
Total number of MAGs	81	51	77	4
Number of MAGs with CGC	80	50	76	4
Average number of CGC	7.6	7.6	7.4	8.5
Minimum number of CGC	0	0	0	4
Maximum number of CGC	22	22	31	12
Number of MAGs with PUL	11	6	11	0
Average number of PUL	18.9	22.5	17	0
Minimum number of PUL	0	0	0	0
Maximum number of PUL	70	70	70	0

Supplementary Table 12. Sum of the average coverage of the 719 MAGs in each metagenome sample. G0, G5, and G10 represent the batch number. Chloramphenicol-treated enrichment cultures grown on xylan did not yield enough DNA for metagenome library construction ("NA").

	Antibiotics-free			+ Penicillin & Streptomycin			+ Chloramphenicol		
	G0	G5	G10	G0	G5	G10	G0	G5	G10
Alfalfa Stem	10407	7536	10407	9087	7505	5834	2550	7	14
Bagasse	7672	6800	6015	6080	7831	6569	2781	11	24
Reed Canary Grass	12414	8672	6969	9953	9212	10424	2367	30	60
Xylan	7787	9534	9299	4530	6718	10960	NA	NA	NA
Average	9570	8136	8173	7413	7817	8447	2566	16	33
Pellets	9653 ± 755								

Supplementary References

1. Segata, N., Börnigen, D., Morgan, X. C. & Huttenhower, C. PhyloPhlAn is a new method for improved phylogenetic and taxonomic placement of microbes. *Nat. Commun.* **4**, 2304 (2013).
2. Letunic, I. & Bork, P. Interactive tree of life (iTOL) v3: an online tool for the display and annotation of phylogenetic and other trees. *Nucleic Acids Res.* **44**, W242–W245 (2016).
3. Seshadri, R. *et al.* Cultivation and sequencing of rumen microbiome members from the Hungate1000 Collection. *Nat. Biotechnol.* **36**, 359–367 (2018).
4. Stewart, R. D. *et al.* Assembly of 913 microbial genomes from metagenomic sequencing of the cow rumen. *Nat. Commun.* **9**, 870 (2018).
5. Zou, Y. *et al.* 1,520 reference genomes from cultivated human gut bacteria enable functional microbiome analyses. *Nat. Biotechnol.* **37**, 179 (2019).
6. Mukherjee, S. *et al.* 1,003 reference genomes of bacterial and archaeal isolates expand coverage of the tree of life. *Nat. Biotechnol.* **35**, 676–683 (2017).
7. O'Leary, N. A. *et al.* Reference sequence (RefSeq) database at NCBI: current status, taxonomic expansion, and functional annotation. *Nucleic Acids Res.* **44**, D733–D745 (2016).
8. Stewart, R. D. *et al.* Compendium of 4,941 rumen metagenome-assembled genomes for rumen microbiome biology and enzyme discovery. *Nat. Biotechnol.* **37**, 953–961 (2019).
9. Simão, F. A., Waterhouse, R. M., Ioannidis, P., Kriventseva, E. V. & Zdobnov, E. M. BUSCO: assessing genome assembly and annotation completeness with single-copy orthologs. *Bioinformatics* **31**, 3210–3212 (2015).
10. Zdobnov, E. M. *et al.* OrthoDB v9.1: cataloging evolutionary and functional annotations for animal, fungal, plant, archaeal, bacterial and viral orthologs. *Nucleic Acids Res.* **45**, D744–D749 (2017).
11. Price, M. N., Dehal, P. S. & Arkin, A. P. FastTree 2 – Approximately Maximum-Likelihood Trees for Large Alignments. *PLOS ONE* **5**, e9490 (2010).
12. Ondov, B. D. *et al.* Mash: fast genome and metagenome distance estimation using MinHash. *Genome Biol.* **17**, 132 (2016).
13. Kurtz, S. *et al.* Versatile and open software for comparing large genomes. *Genome Biol.* **5**, R12 (2004).
14. Olm, M. R., Brown, C. T., Brooks, B. & Banfield, J. F. dRep: a tool for fast and accurate genomic comparisons that enables improved genome recovery from metagenomes through de-replication. *ISME J.* **11**, 2864–2868 (2017).
15. Anderson, M. J. A new method for non-parametric multivariate analysis of variance. *Austral Ecol.* **26**, 32–46 (2001).
16. Oksanen, J. *et al. vegan: Community Ecology Package*. (2019).
17. Huang, L. *et al.* dbCAN-seq: a database of carbohydrate-active enzyme (CAZyme) sequence and annotation. *Nucleic Acids Res.* **46**, D516–D521 (2018).
18. Eddy, S. R. Accelerated Profile HMM Searches. *PLOS Comput. Biol.* **7**, e1002195 (2011).
19. Stewart, R. D., Auffret, M. D., Roehe, R. & Watson, M. Open prediction of polysaccharide utilisation loci (PUL) in 5414 public Bacteroidetes genomes using PULpy. *bioRxiv* 421024 (2018) doi:10.1101/421024.
