# Supplementary material for: Genomic and functional analyses of fungal and bacterial consortia that enable lignocellulose breakdown in goat gut microbiomes
Source: Nat Microbiol. 2021 Feb 1;6(4):499–511. doi: 10.1038/s41564-020-00861-0 (PMC8007473; doi:10.1038/s41564-020-00861-0)
Supplement: Supplementary file 2 — Reporting Summary [file 41564_2020_861_MOESM2_ESM.pdf]

nature research

Corresponding author(s): Michelle A. O'Malley

Last updated by author(s): 10/20/2020

Reporting Summary

Nature Research wishes to improve the reproducibility of the work that we publish. This form provides structure for consistency and transparency in reporting. For further information on Nature Research policies, see Authors & Referees and the Editorial Policy Checklist.

Statistics

For all statistical analyses, confirm that the following items are present in the figure legend, table legend, main text, or Methods section.

n/a	Confirmed	
☐	☒	The exact sample size (*n*) for each experimental group/condition, given as a discrete number and unit of measurement
☐	☒	A statement on whether measurements were taken from distinct samples or whether the same sample was measured repeatedly
☐	☒	The statistical test(s) used AND whether they are one- or two-sided<br>*Only common tests should be described solely by name; describe more complex techniques in the Methods section.*
☒	☐	A description of all covariates tested
☒	☐	A description of any assumptions or corrections, such as tests of normality and adjustment for multiple comparisons
☐	☒	A full description of the statistical parameters including central tendency (e.g. means) or other basic estimates (e.g. regression coefficient) AND variation (e.g. standard deviation) or associated estimates of uncertainty (e.g. confidence intervals)
☐	☒	For null hypothesis testing, the test statistic (e.g. *F*, *t*, *r*) with confidence intervals, effect sizes, degrees of freedom and *P* value noted<br>*Give P values as exact values whenever suitable.*
☒	☐	For Bayesian analysis, information on the choice of priors and Markov chain Monte Carlo settings
☒	☐	For hierarchical and complex designs, identification of the appropriate level for tests and full reporting of outcomes
☒	☐	Estimates of effect sizes (e.g. Cohen's *d*, Pearson's *r*), indicating how they were calculated

Our web collection on statistics for biologists contains articles on many of the points above.

Software and code

Policy information about availability of computer code

Data collection	Chromeleon Chromatography Data System 7, OpenLab CDS ChemStation Edition C.01.02.
Data analysis	USEARCH v11, DADA2 v1.8.0, blast v2.7.1, bowtie2 v2.3..4..1, bbmap v38.00, Trimmomatic v0.36, SPAdes 3.11.1, MetaBat2 v2.12.1, CheckM v1.0.7, dRep v2.2.2, BUSCO v3, PhyloPhlAn v1.10, iTOL 4.4.2, IMG/M v5.0.

For manuscripts utilizing custom algorithms or software that are central to the research but not yet described in published literature, software must be made available to editors/reviewers. We strongly encourage code deposition in a community repository (e.g. GitHub). See the Nature Research guidelines for submitting code & software for further information.

Data

Policy information about availability of data

All manuscripts must include a data availability statement. This statement should provide the following information, where applicable:

- Accession codes, unique identifiers, or web links for publicly available datasets
- A list of figures that have associated raw data
- A description of any restrictions on data availability

The metagenome sequencing reads can be accessed at the Joint Genome Institute under JGI Project ID's and at the NCBI Sequence Read Archive under the SRA ID's listed in Supplementary Data 13. Contigs for each MAG are available at NCBI's Whole Genome Shotgun database under accession numbers SAMN11294286 - SAMN11295004 (Supplementary Table 3) and project number PRJNA530070 (https://www.ncbi.nlm.nih.gov/bioproject/?term=prjna530070).

Field-specific reporting

Please select the one below that is the best fit for your research. If you are not sure, read the appropriate sections before making your selection.

☒ Life sciences ☐ Behavioural & social sciences ☐ Ecological, evolutionary & environmental sciences

For a reference copy of the document with all sections, see nature.com/documents/nr-reporting-summary-flat.pdf

Life sciences study design

All studies must disclose on these points even when the disclosure is negative.

Sample size	In our experiment, we included enrichment cultures based on four different types of substrates in order to investigate the influence of substrate composition on the membership of microbial consortia. We included three antibiotics treatments to separate the fungal and bacterial parts of the source microbiome. We chose to sample from eleven culture generations because previous enrichment cultures in the lab indicate that should be sufficient to allow the community composition of the enrichment cultures to stabilize.
Data exclusions	No data were excluded.
Replication	This study is a large-scale enrichment experiment and it is not feasible for us to perform an experiment that is exactly the same. However, the experimental design of this study itself provides evidence of reproducibility, because the similar types of microbial memberships were enriched in different types of antibiotics treatment, regardless of the type of carbon substrate used in the enrichment culture. Bacteria from phylum Firmicutes dominated antibiotics-free cultures; anaerobic fungi and methanogenic archaea dominated penicillin & streptomycin-treated cultures; anaerobic fungi alone dominated chloramphenicol-treated cultures.
Randomization	This is not relevant to our study because the initial inoculum used for all enrichment cultures was generated by homogenizing multiple fecal pellets before inoculation.
Blinding	Blinding was not relevant to our study because we intended to study the fecal microbiome of an individual goat.

Reporting for specific materials, systems and methods

We require information from authors about some types of materials, experimental systems and methods used in many studies. Here, indicate whether each material, system or method listed is relevant to your study. If you are not sure if a list item applies to your research, read the appropriate section before selecting a response.

Materials & experimental systems

n/a	Involved in the study	
☒	☐	Antibodies
☒	☐	Eukaryotic cell lines
☒	☐	Palaeontology
☒	☐	Animals and other organisms
☒	☐	Human research participants
☒	☐	Clinical data

Methods

n/a	Involved in the study	
☒	☐	ChIP-seq
☒	☐	Flow cytometry
☒	☐	MRI-based neuroimaging
